# Numbers of fish species, higher taxa, and phylogenetic similarity decrease with latitude and depth, and deep-sea assemblages are unique

**DOI:** 10.7717/peerj.16116

**Published:** 2023-09-26

**Authors:** Han-Yang Lin, Shane Wright, Mark John Costello

**Affiliations:** 1 Institute of Marine Science, University of Auckland, Auckland, New Zealand; 2 Faculty of Biosciences and Aquaculture, Nord University, Bodo, Norway

**Keywords:** Latitudinal diversity gradient, Species richness, Phylogenetic indices, Marine fish, Depth zones

## Abstract

Species richness has been found to increase from the poles to the tropics but with a small dip near the equator over all marine fishes. Phylogenetic diversity measures offer an alternative perspective on biodiversity linked to evolutionary history. If phylogenetic diversity is standardized for species richness, then it may indicate places with relatively high genetic diversity. Latitudes and depths with both high species and phylogenetic diversity would be a priority for conservation. We compared latitudinal and depth gradients of species richness, and three measures of phylogenetic diversity, namely average phylogenetic diversity (AvPD), the sum of the higher taxonomic levels (STL) and the sum of the higher taxonomic levels divided by the number of species (STL/spp) for modelled ranges of 5,619 marine fish species. We distinguished all, bony and cartilaginous fish groups and four depth zones namely: whole water column; 0 –200 m; 201–1,000 m; and 1,001–6,000 m; at 5°  latitudinal intervals from 75°S to 75°N, and at 100 m depth intervals from 0 m to 3,500 m. Species richness and higher taxonomic richness (STL) were higher in the tropics and subtropics with a small dip at the equator, and were significantly correlated among fish groups and depth zones. Species assemblages had closer phylogenetic relationships (lower AvPD and STL/spp) in warmer (low latitudes and shallow water) than colder environments (high latitudes and deep sea). This supports the hypothesis that warmer shallow latitudes and depths have had higher rates of evolution across a range of higher taxa. We also found distinct assemblages of species in different depth zones such that deeper sea species are not simply a subset of shallow assemblages. Thus, conservation needs to be representative of all latitudes and depth zones to encompass global biodiversity.

## Introduction

The latitudinal diversity gradient (LDG) has interested ecologists for a long time since it generalizes over local and regional patterns, and thus helps us to understand where species have evolved and survived on both ecological and evolutionary time scales (Ekman, 1953; Gaston, 2000; Willig, Kaufman & Stevens, 2003; Pontarp et al., 2019). Dozens of mechanisms have been proposed to explain the formation of LDG, and many of them are intercorrelated (Gaston, 2000; Jørgensen et al., 2007). Temperature directly and indirectly influences physiology, environmental conditions and ecological interactions which may affect the LDG (Rohde, 1992; Davies et al., 2004; Allen et al., 2006; Wright, Keeling & Gillman, 2006; Wright et al., 2011; Gillman et al., 2010; Brown, 2014). The general LDG of species richness decreasing from the equator to poles has been attributed to the high speciation and low extinction rates in the tropics, with glaciations triggering extirpations and cold temperatures slowing speciation rates in high latitudes (Brown, 2014; Costello & Chaudhary, 2017).

Until recently, the literature presented the typical LDG to be a decrease in species richness from the equator to the poles. However, present LDGs of marine species are now recognized to be bimodal with a dip at or near the equator (Powell, Beresford & Colaianne, 2012; Chaudhary, Saeedi & Costello, 2016; Chaudhary et al., 2021; Chaudhary & Costello, 2023; Arfianti & Costello, 2020; Lin et al., 2021). This dip was absent during the last glaciation (Yasuhara et al., 2020) and has been deepening with climate change since the 1950s (Chaudhary et al., 2021). This temporal variation in the LDG indicates sea temperature is now too high for some species at the equator and that it is the primary driver of the LDG (Chaudhary, 2019; Yasuhara et al., 2020; Chaudhary et al., 2021; Lin et al., 2021).

Species richness is the most common and simplest way to measure biodiversity. However, biodiversity comprises variation within species, between species and amongst ecosystems (Wilson, 1992; Purvis & Hector, 2000). Measures of phylogenetic diversity are based on the phylogenetic tree and provide a view of biodiversity related to evolutionary history (Faith, 1994; Purvis & Hector, 2000; Clarke & Warwick, 2001). Average phylogenetic diversity (AvPD) reflects the phylogenetic relationship of species within an assemblage, and has been applied to latitudinal studies on terrestrial (Fergnani & Ruggiero, 2017) and one study in the marine environment (Wu, Chen & Zhang, 2016). A higher AvPD indicates that species in the assemblage are more (phylogenetically) distantly related. In contrast, a lower AvPD indicates the species are more closely related. Wu, Chen & Zhang (2016) found that AvPD for marine nematodes increased with latitude on the coast of China, showing that nematodes had closer phylogenetic relationships at lower latitudes.

In this study, two additional phylogenetic indices were created to offer a simpler way to understand the higher taxonomic richness and phylogenetic relationship. One was the sum of the higher taxonomic levels (STL) that added the number from classes to genera as a measure of higher taxonomic richness. Because STL is dependent on the number of species present, this study also divided STL by the number of species present (STL/spp) in a given latitude and depth zone to standardize higher taxonomic (phylogenetic) richness for species richness. Thus, a given area with few species but lots of higher taxa will have higher STL/spp (distant phylogenetic relationship). In contrast, a given area with more species but a less or similar number of higher taxa will have lower STL/spp (closer phylogenetic relationship). The concept of STL/spp is similar to AvPD but by directly using the number of taxonomic levels, it is a simpler way to understand the phylogenetic relationship in a given area.

In the marine environment, species richness is also related to depth. Generally, species richness declines with depth, although it may peak at intermediate depths for some taxa in some places (Rex, 1981; McClain & Etter, 2005; Rosa et al., 2008; Costello & Chaudhary, 2017). Depth may also influence the LDG. The environment at the sea surface is generally more variable than near the seabed (Costello et al., 2018a). Variation in sea surface temperature is higher in mid-latitudes than low and polar latitudes (Basher & Costello, 2020). In contrast, the temperature in the deep sea is always low with no significant latitudinal gradient (Costello & Breyer, 2017; Sayre et al., 2017; Basher & Costello, 2020). Therefore, it might be expected that species richness varies across latitudes in the surface zone, but would be more constant in the relatively homogenous cold deep-sea environment. However, studies found that the LDG of brittle stars (Woolley et al., 2016) and marine fishes (Lin et al., 2021) were still bimodal in the deep sea. Therefore, it would be expected that phylogenetic indices would also have clear gradients in the surface water and deep sea with both latitude and depth.

How marine fish composition changes along latitudes in different depth zones, and along depth zones at the global scale has not been studied. A previous global-scale study showed that multiple marine taxa, including fishes, in 5-degree latitude bands could be divided into five assemblages: tropical (between 32.5°S and 27.5°N), two temperate groups, and two polar groups (Chaudhary, 2019). Therefore, it could be expected that the latitudinal distribution of fish species assemblages fits the boundaries of climate zones in the surface water. However, this may not be the case in the deep sea because of the consistently low temperature across latitudes. Furthermore, it could also be expected that species composition would change with depth because the environment in the surface sea is very different to that in the deep sea (Costello et al., 2018a). Marine species usually have geographic distributions determined by their thermal tolerance (Somero, 2010; Sunday, Bates & Dulvy, 2011). Anderson, Tolimieri & Millar (2013) found that latitudinal turnover of demersal fishes in the North-Eastern Pacific between 32.57°N and 48.52°N and between 51 m and 1,200 m depth was significant shallower than 200 m depth but not clear deeper than 800 m, and turnover was greatest at 43°N, 39°N, 35°N, and 31°N. For species composition along with depth, Zintzen et al. (2011) found marine fishes between 0 m and 2,000 m depth could be divided into five assemblages (0–300 m, 300–600 m, 600–900 m, 900–1,200 m, and >1,200 m) in the region of the Norfolk Ridge and Lord Howe Rise (Western Pacific). In the present context, species assemblage clustering (*i.e.,* turnover) may identify latitudes and depths where boundaries may separate assemblages differing in phylogenetic and/or species richness.

Here, we describe species richness and turnover, higher taxonomic richness (STL), phylogenetic relationships using conventional and novel indices (AvPD and STL/spp), and species assemblages of marine fishes across latitudes in the whole water column and three depth zones from 75°S to 75°N, and in depths from 0 m–3,500 m. We also describe gradients among all, bony and cartilaginous fishes to see the difference among fish groups and we illustrate the relationship between the phylogenetic indices and species richness. The hypotheses in this study are that higher taxonomic richness will be highly correlated to species richness, and there will be distinct fish assemblages with latitude and depth. If this is not the case, it would suggest some latitudes or depths have higher recent rates of speciation than others.

## Materials and Methods

### Data

The distribution ranges of all 5,619 fish species for which ranges were available were obtained from AquaMaps (Kaschner et al., 2019) (Table S1). They represent about one-third of all marine fish species (Froese & Pauly, 2019). Five taxonomic levels (class, order, family, genus, and species) were used for calculation of phylogenetic indices. The richness of classes, orders, families, genera, and species in the 5°  latitude band were derived from the taxonomy in FishBase (Froese & Pauly, 2019) as used in Lin et al. (2021). The Fish Tree of Life used 24 genes to generate a phylogenetic tree for 11,368 fish species (Rabosky et al., 2018). However, we found the latitudinal and depth gradients had no significant change when using the taxonomy from FishBase or the Fish Tree of Life (Figs. S1 & S2). Thus, we report results from the former here.

### Phylogenetic indices

Average phylogenetic diversity (AvPD) is a measure of the average phylogenetic distance (branch length) between any two chosen species within a given phylogenetic tree (Clarke & Warwick, 2001). It is calculated by summing the total branch lengths between each pair of species and dividing by the total number of species. When a dataset has species concentrated on a few branches, they will have lower AvPD compared to a dataset with species distributed over many branches. Therefore, a lower AvPD means species are more (phylogenetically) closely related, and a high AvPD means species are more distantly related. AvPD was calculated using PRIMER v6 software (Clarke & Gorley, 2006). Five taxonomic levels (class, order, family, genus, and species) of marine fishes were used based on the taxonomic classification in FishBase (Froese & Pauly, 2019) (Table S1). The assignment of weights (*ω*) to branch lengths adhered to the methodology outlined by Clarke & Warwick (1999). Therefore, the weighting between taxonomic levels is *ω* = 20 for different species in the same genus, *ω* = 40 for species in the same family but different genera, *ω* = 60 for species in the same order but different families, *ω* = 80 when species are in the same order but different classes, and *ω* = 100 when species are in different classes (Clarke & Warwick, 1999; Clarke & Gorley, 2006).

In addition, two new and simple methods of phylogenetic diversity based on the number of the five taxonomic levels in a given latitude band or depth band were applied. One was the sum of the higher taxonomic levels (STL). We used 5 for classes, 4 for orders, 3 for families, and 2 for genera as assignment of weights. Therefore, the equation of STL for each latitude band or depth band in this study was classes ×5 + orders ×4 + families ×3 + genera ×2. For example, the STL of an assemblage with one class, one order, two families and four genera would be 23 from [(1 × 5) + (1 × 4) + (2 × 3) + ( 4 × 2)]. The second simple measure was the sum of the higher taxonomic levels divided by the number of species (STL/spp). This measure was used to account for the number of species because where very few species occur then fewer higher taxa can occur.

### Data analysis

The latitudinal distribution range (northern and southern limits) and the preferred maximum depth were derived from the species geographic and depth ranges. We compared bony (Class Actinopterygii, 5,117 species) and cartilaginous (Class Elasmobranchii, 470 species) fishes. Overall, three groups including “All Fish”, “Bony Fish”, and “Cartilaginous Fish” were analysed for species richness and three phylogenetic indices in the whole water column and three depth zones. The number of taxa in the depth zones among the three fish groups are in Table S5.

The calculation of the species richness and three phylogenetic indices used a 5° latitude band between 75°S and 75°N and four depth zones (whole water column, surface (0–200 m), middle (201–1,000 m), and deep (1,001–6,000 m)) reflecting the photic, mesophotic and aphotic zones of light penetration (Costello & Breyer, 2017). We also calculated the species richness and three phylogenetic indices for every 100 m depth band from the surface to 3,500 m. The phylogenetic indices were only calculated when there were more than 10 species per latitude and depth band to avoid low sample size artefacts following Plazzi, Ferrucci & Passamonti (2010). The raw values of species richness, AvPD, STL, and STL/spp in 5° latitude bands among four depth zones and 100 m depth bands among all, bony, and cartilaginous fishes are shown in Tables S2–S5. A quadratic polynomial regression was used to prevent overfitting of the relationship between the indices AvPD, STL, STL/spp, and species richness with depth among all, bony, and cartilaginous fishes. It was a better fit to the data than linear and exponential regressions, indicating that these indices may not show a simple gradient with depth.

The Jaccard similarity coefficient (Jaccard, 1912) was used to estimate fish species assemblage similarities and turnover as it is the simplest and most widely used measure (Kreft & Jetz, 2010), including for marine fishes and other taxa (Angel et al., 2007; Anderson, Tolimieri & Millar, 2013; Navarrete, Lagos & Ojeda, 2014) and it produces the same patterns as alternative similarity coefficients at global scales (Costello et al., 2017; Costello et al., 2018b):

\begin{eqnarray*}{J}_{i,j}= \frac{a}{a+b+c} \end{eqnarray*}

where *a* is the number of species that are common in samples *i* and *j*, *b* is the number of species present in sample *i* but absent in sample *j*, and *c* is the number of species present in sample *j* and absent in sample *i* (Jaccard, 1912). The index was clustered using the unweighted pair group method with arithmetic mean (UPGMA), and the similarity profile routine (SIMPROF) test used to determine which latitude and depth bands had a significantly different species composition at the 95% level using PRIMER v6 (Clarke & Gorley, 2006).

## Results

### Species richness

**Figure 1:**
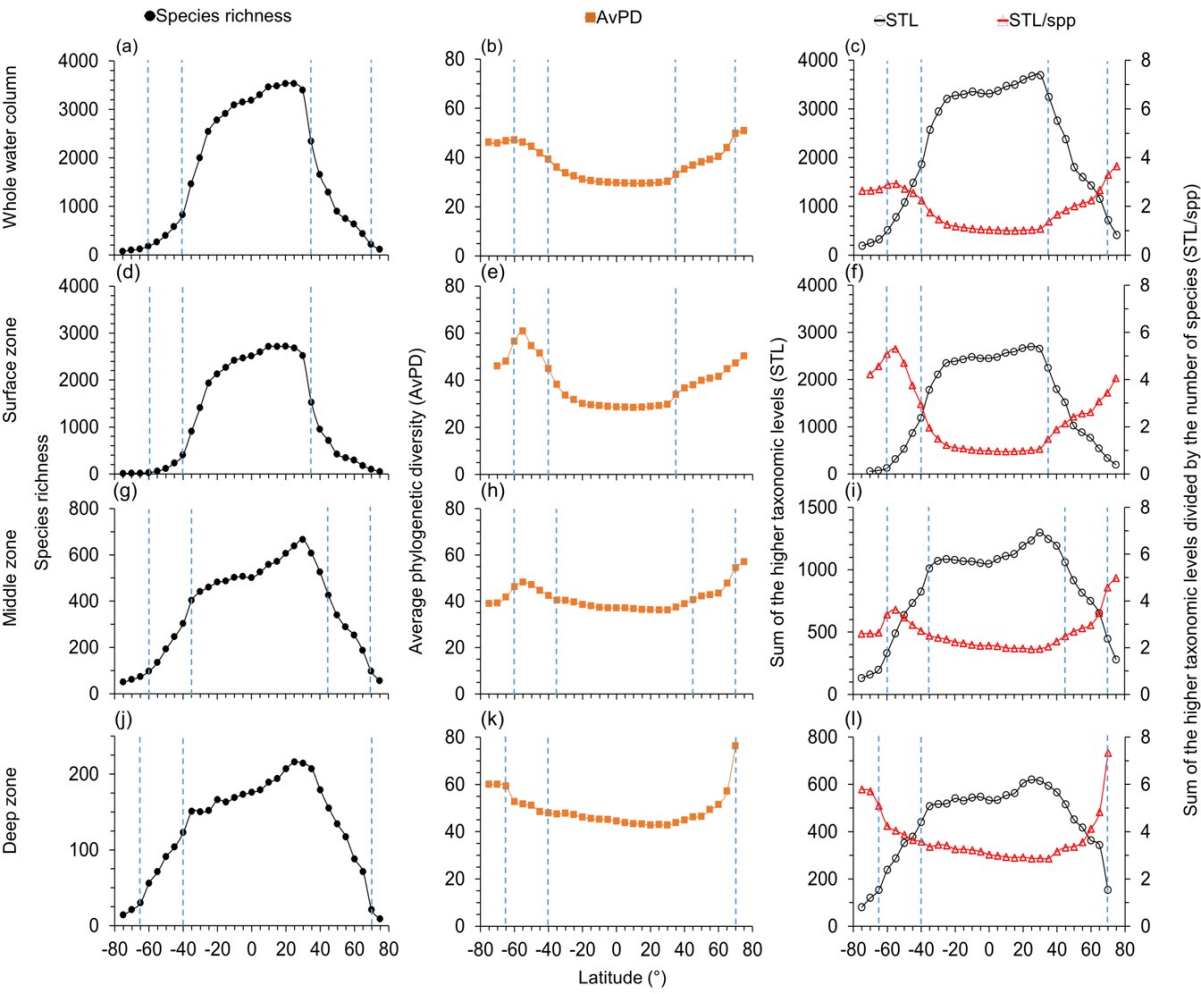
Latitudinal gradients for all species. For all fish, latitudinal gradients of species richness (A, D, G, J), average phylogenetic diversity (AvPD, B, E, H, K), the sum of the higher taxonomic levels (STL) and the sum of the higher taxonomic levels divided by the number of species (STL/spp) (C, F, I, L) between 75° S and 75° N in the whole water column, surface (0 m–200 m), middle (201 m–1,000 m), and deep (1,001 m–6,000 m) zone. The dashed lines indicate the distinct fish assemblages from clustering results in Fig. 7. Note axes scales vary.

Species richness of all and bony fish had similar latitudinal gradients in each depth zone (Fig. 1 & Fig. S3). Because the gradients of all and bony fish were very similar in the latitudes and depths, we moved the gradients of bony fish to the Supplemental Information to reduce the number of figures here.

**Figure 2:**
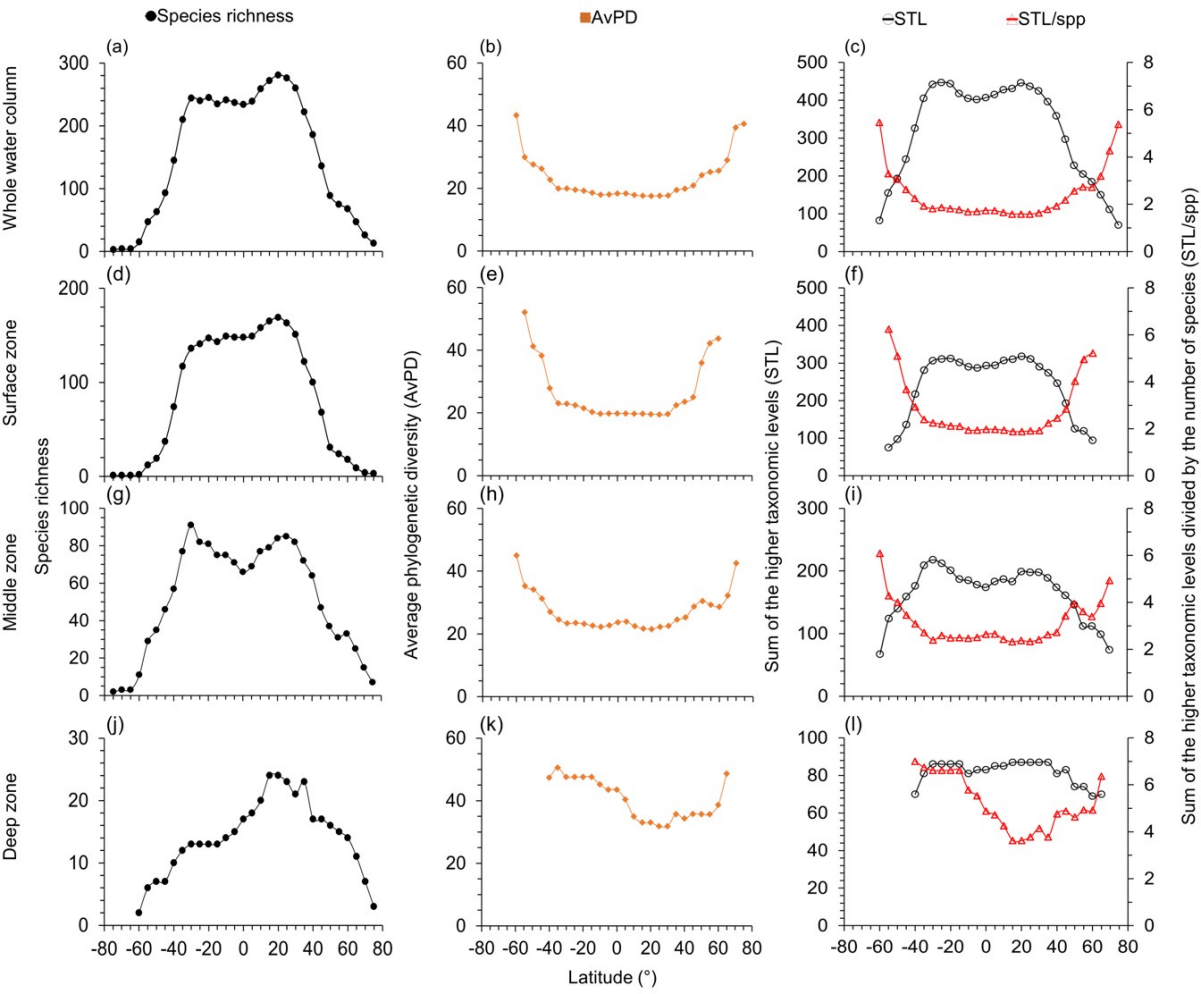
Latitudinal gradients for cartilagenous fish. For cartilaginous fish, latitudinal gradients of species richness (A, D, G, J), average phylogenetic diversity (AvPD; B, E, H, K), the sum of the higher taxonomic levels (STL) and the sum of the higher taxonomic levels divided by the number of species (STL/spp) (C, F, I, L) between 75° S and 75° N in the whole water column, surface (0 m–200 m), middle (201 m–1,000 m), and deep (1,001 m–6,000 m) zone. Note axes scales vary.

The highest species richness was at 10°N–15°N in the whole water column (Figs. 1A) and surface zones (Fig. 1D & Fig. S3D). Highest species richness was at 30°N in the middle (Fig. 1G & Fig. S3G) and deep zones (Fig. 1J & Fig. S3J). Similarly, species richness of cartilaginous fishes was highest at 20°N –25°N in each depth zone (Fig. 2). In addition, species richness of cartilaginous fishes was bimodal in the surface (Fig. 2D) and middle zones (Fig. 2G). Species richness among all, bony, and cartilaginous fishes all had a small dip at the equator, was higher at the tropics and subtropics, and decreased with latitude in all depth zones (Figs. 1 and 2, & Fig. S3).

### Phylogenetic indices in the whole water column

Both species richness and the sum of higher taxonomic levels (STL) were low in polar latitudes and high in the tropics and subtropics in all depth zones (Fig. 1). In contrast, when adjusted for species richness as the average phylogenetic diversity (AvPD) and the sum of the higher taxonomic levels divided by the number of species (STL/spp), phylogenetic diversity for all, bony, and cartilaginous fish groups were lower at tropical and subtropical areas (between 30°S and 30°N) but higher at high latitudes. At higher latitudes, AvPD and STL/spp increased and peaked at 55°S and 75°N, respectively (Figs. 1B, 1C, 2B, 2C & Figs. S3B, S3C). However, in the Southern Ocean, the AvPD and STL/spp of all and bony fishes slightly decreased indicating that species there were more phylogenetically closely related relative to species at other high latitude areas (Figs. 1B, 1C & Figs. S3B, S3C).

The STL among all, bony and cartilaginous fishes showed a similar gradient to species richness with a small dip at the equator (Figs. 1C, 2C, & Fig. S3C). The STL of all and bony fishes peaked at 25°N, then decreased poleward (Fig. 1C & Fig. S3C). The STL of cartilaginous fishes was bimodal, peaking at 30°S and 25°N, respectively (Fig. 2C). Thus, subtropical latitudes had slightly more species from more different higher taxonomic levels than at the equator.

### Phylogenetic indices in depth zones

The latitudinal gradients of AvPD, STL and STL/spp of all, bony and cartilaginous fish groups in the surface zone were similar to the gradients in the whole water column (Figs. 1 and 2 & Fig. S3). This is because about 73% of all fishes, 75% of bony fishes, and 58% of cartilaginous fishes were in the surface zone, and their distribution influenced the latitudinal gradients of the whole water column more than species in other depths. In addition, latitudinal gradients of AvPD, STL, and STL/spp for all and bony fishes were also similar in different depth zones because 91% of all fish species were bony fish in our dataset. The main differences were that the gradients became less pronounced with depth and more variable when there were fewer species.

AvPD and STL/spp of all, bony and cartilaginous fishes were all lower between 30°S and 30°N in the surface and middle zones and between 40°S and 40°N in the deep zone, and they all peaked at 55°S and 75°N (Figs. 1 and 2, & Fig. S3). In addition, AvPD and STL/spp of all and bony fishes decreased in the surface and middle zones of the Southern Ocean but increased in the deep zone of the Southern Ocean (Figs. 1 and 2 & Fig. S3). There were no phylogenetic data for cartilaginous fishes in the high southern latitudes because there were less than 10 species present (Fig. 2). This result showed that all bony and cartilaginous fish species had closer phylogenetic relationships in the tropics and subtropics than high latitudes in all depth zones.

The STL of all and bony fishes in the surface, middle and deep zones were all similar to species richness, being highest in the northern subtropical areas (between 25°N and 35°N), and they all had a small dip at the equator in the three depth zones (Fig. 1 & Fig. S3). For cartilaginous fishes, STL was also similar to species richness in the surface (Fig. 2F) and middle zones (Fig. 2I) but not in the deep zone (Fig. 2L). In the deep zone, the species richness of cartilaginous fishes was highest at 15°N–35°N (Fig. 2J). However, STL of cartilaginous fishes was flattened across the tropics and subtropics in the deep zone. Thus, cartilaginous fishes had similar higher taxonomic richness across the tropics and subtropics in the deep sea (Fig. 2L).

Overall, we found that the species were on average more phylogenetically closely related in the tropics and subtropics (between 30°S and 30°N) in all four depth zones. In contrast, the species assemblages in the southern temperate latitudes and Arctic Ocean were more phylogenetically distantly related in all depth zones. The species in the Southern Ocean were more closely phylogenetically related in the surface and middle zones, but not in the deep zone compared to the temperate latitudes (Figs. 1 and 2, & Fig. S3).

### Relationships of phylogenetic indices and species richness

**Figure 3:**
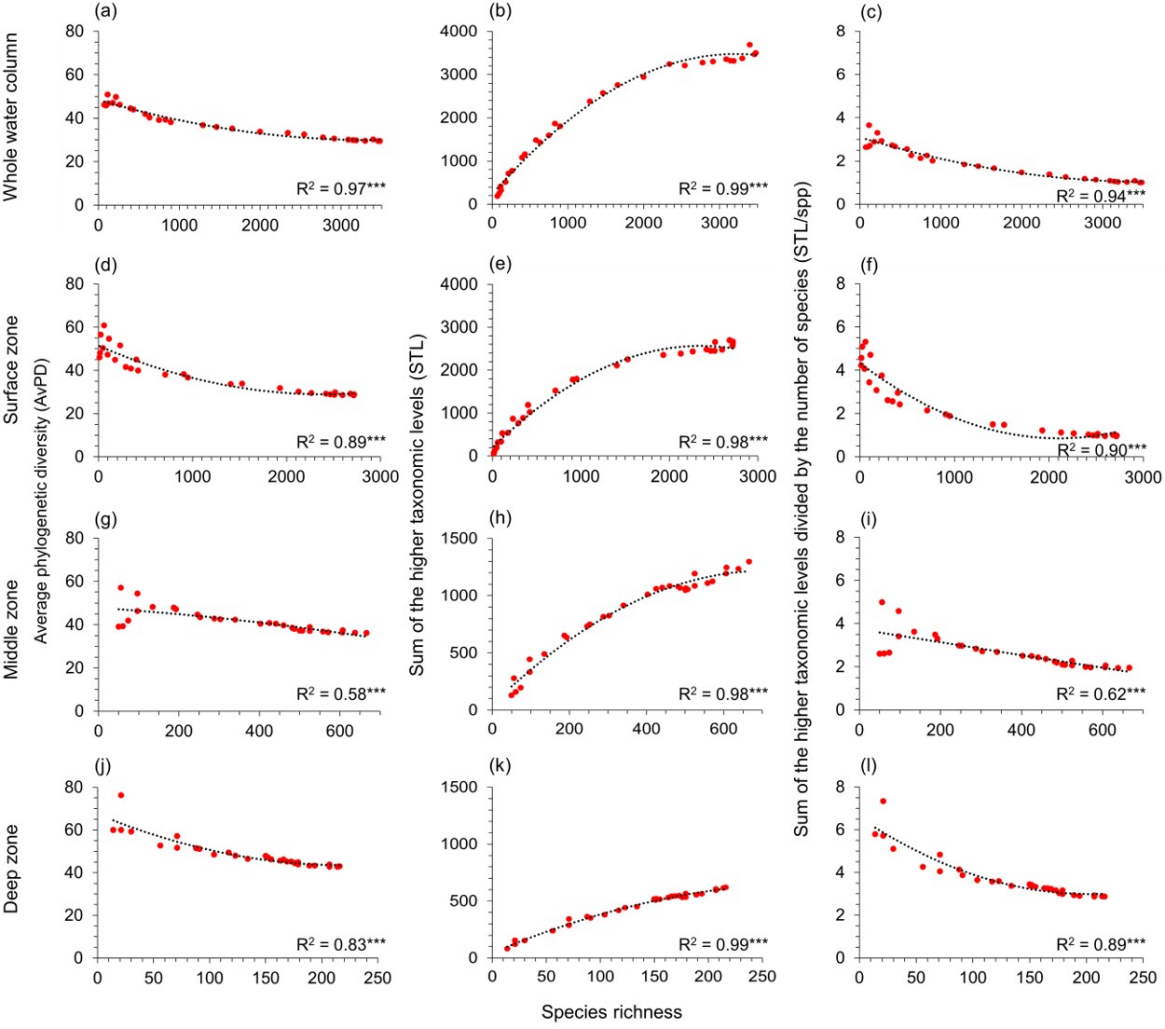
Gradients in depth zones for all fish. For all fish, polynomial regression of species richness, average phylogenetic diversity (AvPD; A, D, G, J), the sum of the higher taxonomic levels (STL; B, E, H, K)), and the sum of the higher taxonomic levels divided by the number of species (STL/spp; C, F, I, L) in 5-degree latitude bands in the whole water column, surface (0 m–200 m), middle (201 m–1,000 m), and deep (1,001 m–6,000 m) zone. Asterisks (***) indicate *p*-value < 0.001. Note axes scales vary.

**Figure 4:**
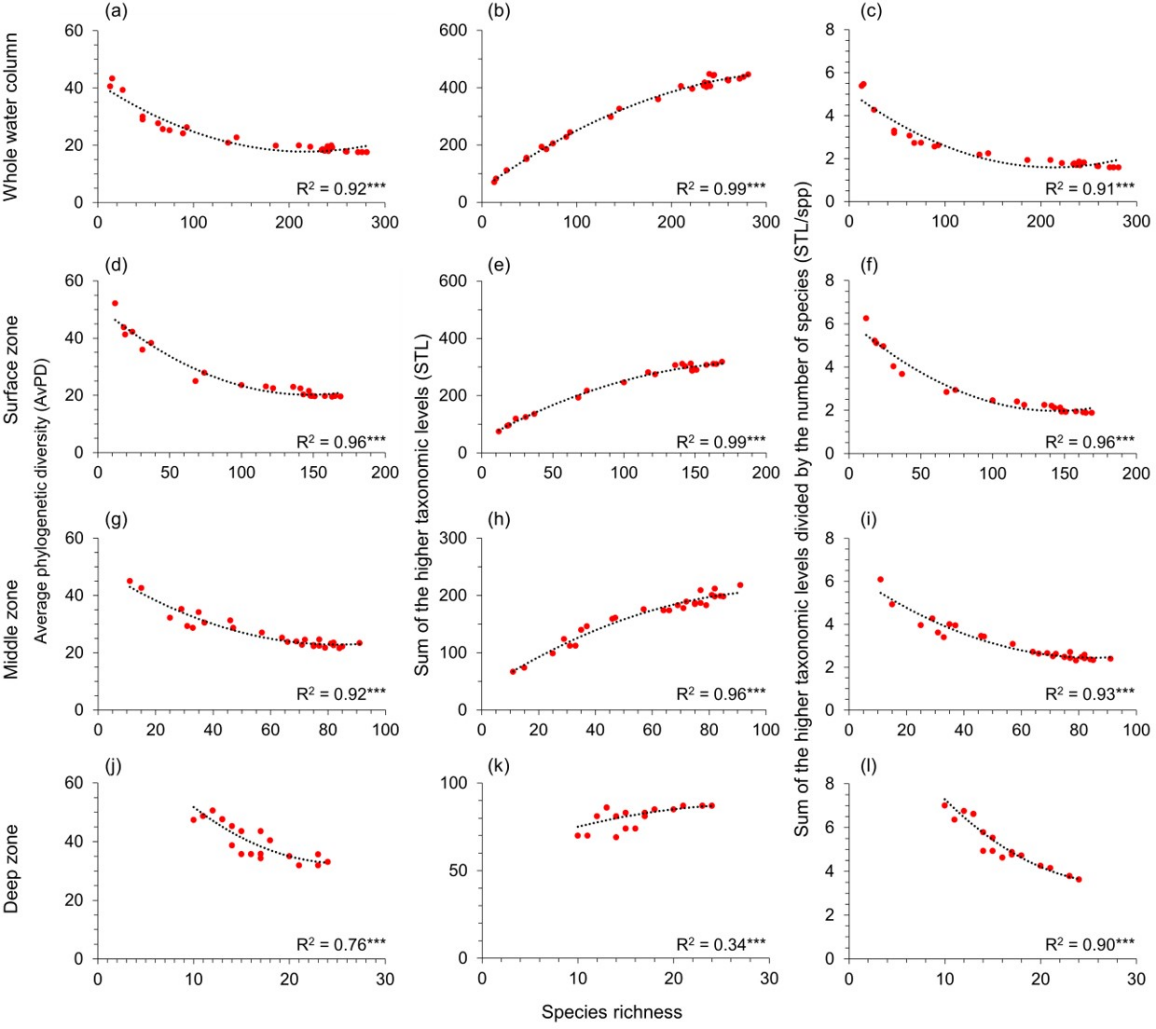
Gradients in depth zones for cartilagenous fish. For cartilaginous fish, polynomial regression of species richness, average phylogenetic diversity (AvPD; A, D, G, J), the sum of the higher taxonomic levels (STL; B, E, H, K), and the sum of the higher taxonomic levels divided by the number of species (STL/spp; C, F, I, L) in 5-degree latitude bands in the whole water column, surface (0 m–200 m), middle (201 m–1,000 m), and deep (1,001 m–6,000 m) zone. Asterisks (***) indicates *p*-value < 0.001. Note axes scales vary.

AvPD, STL, and STL/spp were all significantly correlated to species richness among all, bony, and cartilaginous fishes in the whole water column, surface, middle, and deep zones (*R*^2^ = 0.34–0.99, *p*-values <0.001) (Figs. 3 and 4, & Fig. S4). These relationships were not linear. Most latitudes with more species had lower AvPD and STL/spp for all, bony and cartilaginous fishes. However, both higher and lower indices of AvPD and STL/spp for all and bony fishes existed in the areas where species richness was low. This was because both the Arctic Ocean and the Southern Ocean had low species richness, but species were phylogenetically more distantly related (higher AvPD and STL/spp) in the Arctic than the Southern Ocean (Figs. 3 and 4, & Fig. S4).

That the STL of all, bony, and cartilaginous fishes increased with species richness among four depth zones reflected that areas with high species richness also had higher phylogenetic richness (Figs. 3 and 4, & Fig. S4). However, this pattern was weaker for cartilaginous fishes in the deep zone (Fig. 4K).

### Depth gradients

**Figure 5:**
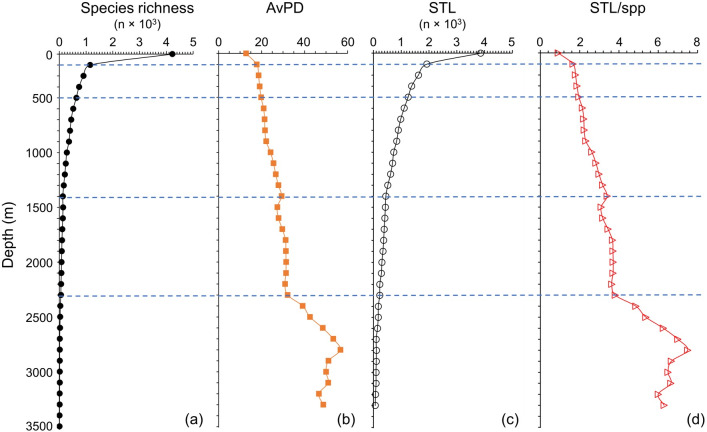
Depth gradients for all fish. For all fish, gradients of (A) species richness, (B) average phylogenetic diversity (AvPD), (C) the sum of the higher taxonomic levels (STL), and (D) the sum of the higher taxonomic levels divided by the number of species (STL/spp) in 100 m depth bands from 0 m to 3,500 m. The dashed lines indicate the distinct fish assemblages as in Fig. 8.

**Figure 6:**
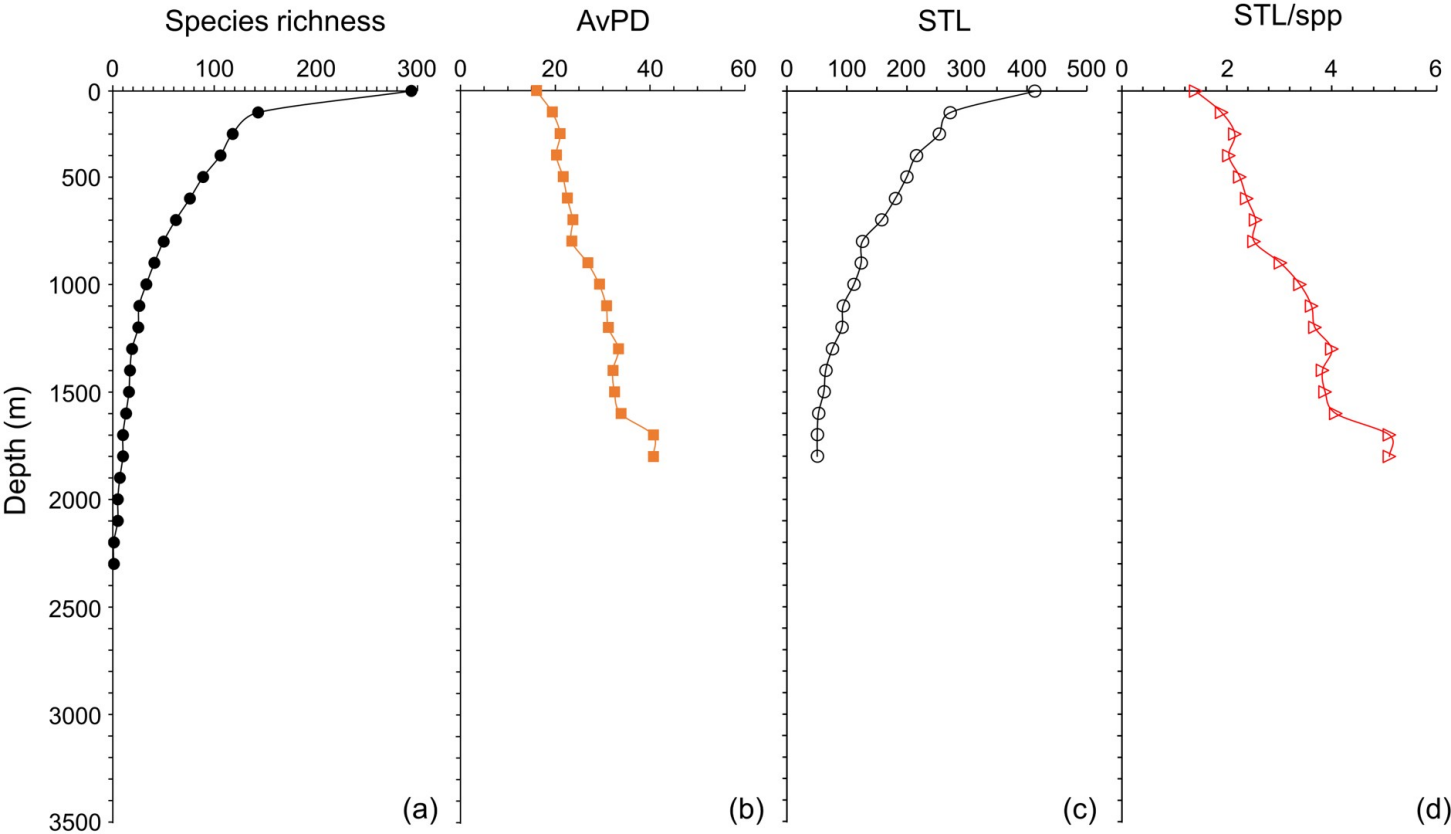
Depth gradients for cartilagenous fish. For cartilaginous fish, gradients of (A) species richness, (B) average phylogenetic diversity (AvPD), (C) the sum of the higher taxonomic levels (STL), and (D) the sum of the higher taxonomic levels divided by the number of species (STL/spp) in 100 m depth bands from 0 m to 3,500 m.

Species richness and STL among all, bony and cartilaginous fish groups were all highest in the shallow water (<100 m), then decreased with depth (Figs. 5 and 6 & Fig. S5). There were fewer than 10 species deeper than 3,300 m for all and bony fishes, and deeper than 1,800 m for cartilaginous fishes (Figs. 5 and 6, & Fig. S5). In our dataset, there were no cartilaginous fishes deeper than 2,300 m (Fig. 6A).

The AvPD and STL/spp were all lowest shallower than 100 m and they increased with depth among all, bony and cartilaginous fishes (Figs. 5 and 6, & Fig. S5). AvPD and STL/spp peaked at ∼2700 m for all and bony fishes (Figs. 5B, 5D & Figs. S5B, S5D), and at ∼1,700 m for cartilaginous fishes (Figs. 6B, 6D). Thus, both species richness and higher taxonomic richness (STL) decreased with depth, and with increased depth, the phylogenetic relationships (AvPD, STL/spp) of species became more distant (*i.e.,* less phylogenetically similar).

### Species assemblages by latitude

**Figure 7:**
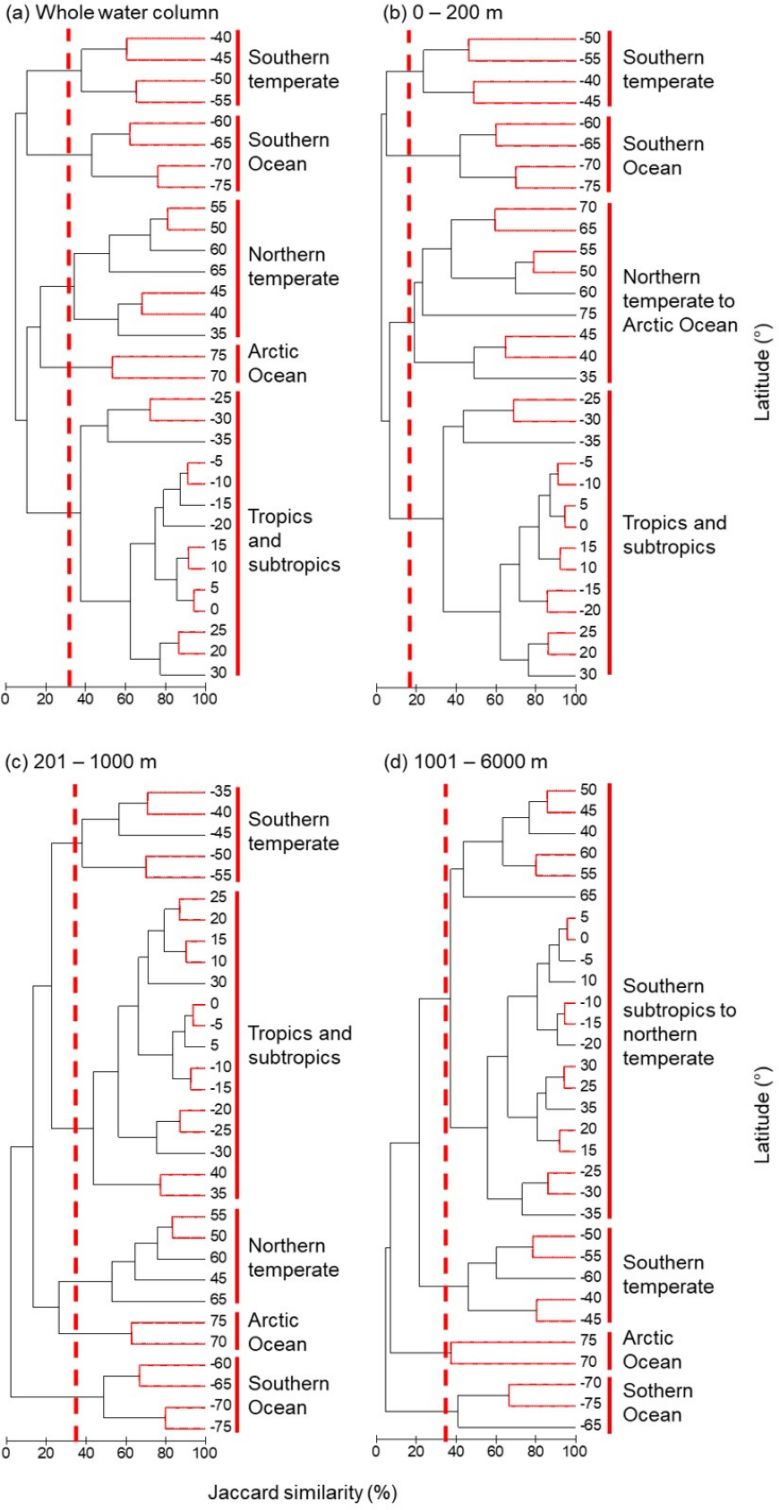
Smilarity clustering of all species by latitude. Clustering of species assemblages in 5-degree latitude bands from 75° S to 75° N in the (A) whole water column, (B) surface zone (0 m–200 m), (C) middle zone (201 m–1,000 m), and (D) deep zone (1,001 m–6,000 m). The dashed lines show the cut-offs, and the solid lines indicate the species assemblages. Red lines between latitudes indicate no significant difference between assemblages (SIMPROF test, *P* < 0.05).

Fish species assemblages formed spatially coherent clusters by latitudinal bands (Fig. 7). The SIMPROF test showed significant differences over scales of 15°  latitude but often not at 5°. Over all depth zones, and shallower than 200 m, there were distinct tropical and subtropical (± 30°  latitude), and southern and northern hemisphere groups (Figs. 7A, 7B). Within these groups, there were five assemblages: Arctic Ocean (>70°N), northern temperate (35°N–65°N), southern temperate (40°S–55° S), Southern Ocean (60°S–75°S), and tropical and sub-tropical assemblages (Fig. 7A).

In contrast to the surface zone, below 200 m depth the first division in the dendrogram separated out the Southern Ocean, indicating its fish fauna was dissimilar from all the rest (Figs. 7C, 7D). The second most distinct assemblage below 200 m was the Arctic Ocean. Thus, in the deep sea the polar assemblages are the least similar to the rest of the world fish fauna. In both the middle and deep depth zones, there were distinct tropical and subtropical groups between 35°S and ∼35°N, and northern and southern hemisphere groups. These results show the different latitudinal biogeographic distribution for the shallow and deep-sea fish assemblages, with the polar assemblages being most distinct below 200 m depth.

**Figure 8:**
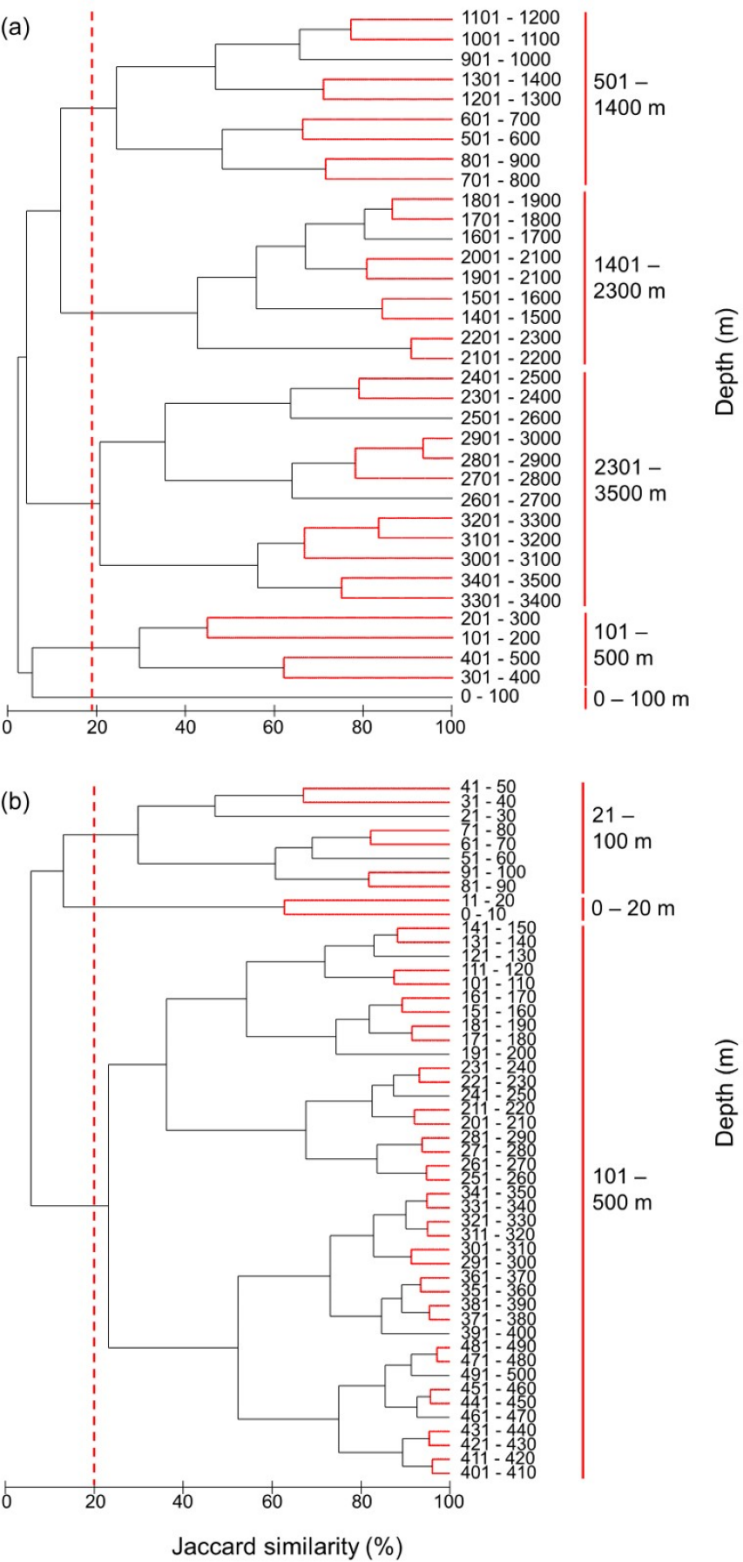
Clustering of all species by depth. Clustering of species assemblages along with depth in the (A) 100 m depth bands from 0 m to 3,500 m and (B) 10 m depth bands from 0 m to 500 m. The dashed lines show the cut-offs, and the solid lines indicate the species assemblages. Red lines between latitudes indicate no significant difference between assemblages (SIMPROF test, *P* < 0.05).

### Fish assemblages by depth

The most distinct fish assemblages separated at 500 m, closely followed by a shallower than 100 m, 101–500 m, 501–1,400 m, 1,401–2,300 m, and deeper than 2,301 m (Fig. 8A). Because most species live in the surface zone, we also clustered the species in 10 m depth bands from 0 m to 500 m. Although further subdivisions of depth zones were suggested by the SIMPROF test, we are cautious in discriminating these using the present modelled range data. Three fish assemblages, 0–20 m, 21–100 m, 101–500 m, were grouped at the same similarity level as in the 100 m band analysis (Fig. 8B). Therefore, there was increasing similarity and homogeneity in fish assemblages with depth.

## Discussion

We found higher species and taxonomic richness (STL) within the tropics and subtropics, with a slight dip at the equator, and similar correlations across fish groups and depth zones. Phylogenetic relationships of species assemblages demonstrated greater closeness (lower AvPD and STL/spp) in warmer environments, encompassing lower latitudes and shallower waters, compared to colder environments, which included higher latitudes and deeper seas. Additionally, distinct species assemblages were evident across various depth zones, indicating that deeper sea species are not mere subsets of shallow-water assemblages. Here, we discuss the possible mechanisms for our findings.

### Latitudinal gradients

In general, we found gradients of higher taxonomic richness (STL) were similar to gradients of species richness among fish groups and depth zones. The only difference was that the species richness of cartilaginous fishes in the deep zone peaked at 15°N–35°N (Fig. 2J), but the latitudinal gradient of STL was flattened across the tropics and subtropics (Fig. 2L). This was because there were only 21–24 cartilaginous fishes in the deep zone at 15°N–35°N, and four of the genera (*i.e., Apristurus*, *Bathyraja*, *Galeus*, *Rajella*) contained more species than other genera.

### Equatorial dip

Species richness and STL of all, bony, and cartilaginous fishes all peaked in the northern subtropics with a small dip near the equator. Using the same dataset as the present study, Lin et al. (2021) found that species richness of marine fishes decreased when the mean annual temperature was greater than 25 °C. Previous studies similarly indicated that some tropical species cannot tolerate present temperatures at the equator, and they move into subtropical latitudes in concert with global warming (Garciá Molinos et al., 2016; Chaudhary, 2019; Yasuhara et al., 2020; Chaudhary et al., 2021; Chaudhary & Costello, 2023). Furthermore, species richness across all taxa, including pelagic fish, declines above 20 °C, except for reef fish where it peaks at 25 °C (Chaudhary et al., 2021). The present analysis indicates that these latitudinal shifts also apply not only to fish species, but similarly to higher taxa.

### Phylogenetic gradients with latitude

Our results showed that the latitudinal gradients in species richness of all, bony and cartilaginous fishes were significantly correlated with three phylogenetic indices among all depth zones. Areas with more species, such as the tropics and subtropics (between 30°S and 30°N) had relatively lower phylogenetic similarity (low AvPD and STL/spp) despite higher taxonomic richness (high STL). That is, the tropics and subtropics not only had more species with a closer phylogenetic relationship but had more diverse higher taxonomic levels. In contrast, areas with low species richness, such as temperate areas, had higher AvPD and STL/spp but lower STL reflecting that species within assemblages at high latitudes were less phylogenetically related and from fewer higher taxonomic levels. In addition, the results showed that phylogenetic similarity (lower AvPD and STL/spp) was lower in the tropics and subtropics, but higher at 55°S and 75° N, respectively, from the surface to the deep depth zone. These results confirmed the initial expectation that phylogenetic indices would have clear latitudinal gradients because the species richness also had clear latitudinal gradients in all depth zones (Woolley et al., 2016; Lin et al., 2021).

Here, we suggest two reasons that may explain the findings that species and higher taxonomic richness were more diverse and species assemblage was more phylogenetically closely related in the tropics and subtropics than temperate latitudes.

First, temperature is the main driver affecting marine biodiversity at a global scale. The higher temperature in the tropics, resulting in shorter generation times, higher rates of metabolism, faster rates of mutation, and faster selection, which generate and maintain higher biodiversity (Rohde, 1992; Wright et al., 2011; Brown, 2014; Costello & Chaudhary, 2017). Empirical studies that analyzed large datasets on several different taxa from the terrestrial to the marine environment, such as angiosperm families (Davies et al., 2004), rain forest plants (Wright, Keeling & Gillman, 2006; Gillman et al., 2010), marine foraminifera (Allen et al., 2006), and marine fishes (Wright et al., 2011) also found that rates of evolution were greater in the tropics. Although there are claims indicated that marine fishes had higher speciation rates at both poles (Rabosky et al., 2018; Friedman & Muñoz, 2023), this was based on the interpretation of molecular data which is subject to many confounding variables and their use in inferring speciation or diversification is questionable (*e.g.*, Louca & Pennell, 2020). Further support for higher speciation and diversification in the tropics includes that the number of endemic freshwater fish species is highest in low latitude lakes when lake size and age are accounted for (Hanly, Mittelbach & Schemske, 2017), and the number of endemic marine fish is highest in the Coral Triangle near the equator (Asaad et al., 2018). Moreover, genetic diversity in marine fish shows a similar LDG to our findings for species richness, including a dip at the equator (Manel et al., 2020).

This pattern of higher tropical speciation and diversification is not limited to marine species and fish; a global analysis of the LDG in mammals found speciation and diversification rates higher, and extinction and dispersal rates lower, in the tropics to subtropics than temperate latitudes (Rolland et al, 2014). Similarly, warmer temperature shortens the duration of planktonic eggs and larvae in the tropics (O’Connor et al., 2007; Álvarez Noriega et al., 2020), resulting in reduced gene flow and higher rates of diversfication and speciation in the tropics and subtropics (O’Connor et al., 2007).

It has been suggested that the absence of glaciations (that would have extirpated polar fauna during ice ages) has allowed more time for speciation and lower extinction rates in the tropics (Pellissier et al., 2014; Leprieur et al., 2016; Miller et al., 2018). However, interglacials may have extirpated species in low latitudes due to excessive temperatures (Reddin et al., 2022), thereby limiting speciation there. While there is evidence of recent and past temperature-driven declines in species in low latitudes, including local extirpations, these species could have survived by shifting their range into higher latitudes as is already occurring due to anthropogenic climate change (Chaudhary et al., 2021; Reddin et al., 2022). Thus, rather than glacial extinction in polar regions being a cause of present species and taxonomic richness latitudinal gradients, it is more simply explained by temperature alone because species would have shifted their latitudinal distribution to maintain their preferred thermal niche.

Second, habitat complexity and niche diversity are higher in the tropics and subtropics. The tropics and subtropics have higher habitat complexity, notably coral reefs which contain 27% of marine fish species (Costello, 2015), that provide more ecological niches leading to higher species diversity (Rocha et al., 2005; Grosberg, Vermeij & Wainwright, 2012; Kovalenko, Thomaz & Warfe, 2012).

Together these reasons allow phylogenetic diversity and species to originate and accumulate, even in the deep sea of the tropics and subtropics because species’ may extend their distribution from the shallow water to the deep sea (Friedman & Muñoz, 2023). In contrast, the colder and seasonal environment at high latitudes limits growth and slows generations times (Meseguer & Condamine, 2020; Yasuhara et al., 2020). Therefore, fish assemblages at high latitudes are more phylogenetically distantly related than they are at low latitudes, reflecting the higher tropical and sub-tropical speciation rates.

### Southern Ocean

Species richness, AvPD, STL and STL/spp of all and bony fishes decreased in the Southern Ocean in the whole water column, surface, and middle zones. The Southern Ocean has been a relatively enclosed environment compared to adjacent southern temperate latitudinal areas since the opening of the Drake Passage 23–25 million years ago, the formation of the Antarctic Circumpolar Current, and the subsequent ocean cooling, resulting in the evolution of a unique fish fauna with a high rate of endemism (McGonigal & Woodworth, 2002; Duhamel et al., 2014), and over 45% of all its marine species are endemic (Costello et al., 2010). The fish assemblage is dominated by the Notothenioidei (notothenioids) which accounts for over 35% of fish species in the Southern Ocean (Eastman, 1997). Other major fish groups are the Zoarcidae (eelpouts) and the Scorpaeniform family Liparidae (snailfish) (Eastman & McCune, 2000). The adaptive radiation of Antarctic notothenioids is characterized by the presence of antifreeze glycoproteins, originating near the Oligocene-Miocene transition (mean 23.9 million years ago) during a major period of global cooling and ice-sheet extension. This allowed notothenioids to live and evolve in this cold environment (McGonigal & Woodworth, 2002; Duhamel et al., 2014). In contrast to this stability of the Southern Ocean developing an endemic fauna, the Arctic Ocean is an extension of the Pacific and Atlantic Ocean, and is a more environmentally variable environment (Lin & Costello, 2023). Thus, the species richness of the Arctic is higher than of the Antarctic and Southern Ocean fish fauna (Fig. 1, Lin & Costello, 2023).

### Depth gradients

Species and phylogenetic richness not only declined into higher, colder latitudes but also with depth in the ocean. Similarly, zooplankton genetic diversity declined with depth to 1,500 m in the North Pacific subtropical gyre (Sommer et al., 2017). Because temperature variation with depth is less in high latitude, there is greater phylogenetic similarity amongst fish with depth in higher latitudes, and thus lower overall phylogenetic diversity compared to the tropics, as noted by Friedman & Muñoz (2023).

Our results showed that species richness and STL among all, bony and cartilaginous fish groups were all highest in the shallowest waters and decreased with depth. This is consistent with the shallow depths being the driver for deep-sea species origins (Miller et al., 2022). In contrast, AvPD and STL/spp increased with depth. In shallower depths, phylogenetic relationships between species were closer reflecting higher rates of speciation due to greater productivity, generally warmer temperatures, and habitat complexity. Shallow water has the highest species richness and taxonomic richness (STL), and thus appears to be an “engine of speciation” for marine fishes as it is for marine species overall (Costello & Chaudhary, 2017; Eme et al., 2020; Miller et al., 2022).

Deeper than 2,300 m, there was only one class (*i.e.,* Actinopterygii) and most of the species were from different families. At a depth of 2,701–2,800 m, 3 of 14 species were from the family Macrouridae, and the other 11 species were all from different families. Therefore, the fishes at 2,700–2,800 m were far less closely related than in shallower depths.

We found that, as expected, the extent of marine fishes’ depth ranges, as indicated by species assemblages occupying larger depth intervals, declined with depth. Zooplankton genetic diversity shows a similar pattern with depth (Sommer et al., 2017). There were three distinct assemblages shallower than 500 m (Fig. 8B), and only three assemblages deeper than 500 m (Fig. 8A). This supports the hypothesis that species richness declines with depth because of decreasing environmental heterogeneity, productivity and temperature (Costello & Chaudhary, 2017; Costello et al., 2018a). Thus, deeper assemblages occupy larger depth intervals and spatial area, with lower species turnover and biogeographic distinctiveness.

## Conclusions

The analyses in this study found that species richness and higher taxonomic richness (STL) were higher in the tropics and subtropics than in high latitudes, with a small dip at the equator in all fish groups and depth zones. In addition, species assemblages had closer phylogenetic relationships (lower AvPD and STL/spp) in warmer (low latitudes and shallow water) than colder environments (high latitudes and deep sea). This result was significantly related to species richness and different fish groups among latitudes and depth zones. The results in this study support the hypothesis that a warmer temperature environment fosters speciation and thus generates higher biodiversity and a closer phylogenetic relationship. However, the cold environment in the Southern Ocean was dominated by endemic notothenioids, so it had a closer phylogenetic relationship than other temperate latitudes because of its unique isolated environment.

##  Supplemental Information

10.7717/peerj.16116/supp-1 Supplemental Information 1 Supplemental Figures and Tables

## Additional Information and Declarations

### Data Availability

The following information was supplied regarding data availability: The list of species analysed and the distribution ranges of all 5,619 fish species for which ranges were available are available as a Supplementary File.
