# Supplementary material for: Numbers of fish species, higher taxa, and phylogenetic similarity decrease with latitude and depth, and deep-sea assemblages are unique
Source: PeerJ. 2023 Sep 26;11:e16116. doi: 10.7717/peerj.16116 (PMC10541023; doi:10.7717/peerj.16116)
Supplement: Supplemental Information 1 [file peerj-11-16116-s001.docx]

Supporting Information for

**Numbers of fish species, higher taxa, and phylogenetic similarity decrease with latitude and depth, and deep-sea assemblages are unique**

**Table S1:** Taxonomic levels of the 5,619 marine fish used in this analysis.

| **Actinopterygii** | ***Gorgasia*** | *Gymnothorax marshallensis* | *Apterichtus equatorialis* |
| --- | --- | --- | --- |
| **Acipenseriformes** | *Gorgasia punctata* | *Gymnothorax melatremus* | *Apterichtus kendalli* |
| **Acipenseridae** | ***Heteroconger*** | *Gymnothorax meleagris* | ***Bascanichthys*** |
| ***Acipenser*** | *Heteroconger digueti* | *Gymnothorax miliaris* | *Bascanichthys bascanium* |
| *Acipenser brevirostrum* | *Heteroconger hassi* | *Gymnothorax monostigma* | ***Brachysomophis*** |
| *Acipenser gueldenstaedtii* | ***Japonoconger*** | *Gymnothorax mordax* | *Brachysomophis cirrocheilos* |
| *Acipenser medirostris* | *Japonoconger africanus* | *Gymnothorax moringa* | ***Callechelys*** |
| *Acipenser mikadoi* | ***Paraconger*** | *Gymnothorax nigromarginatus* | *Callechelys catostoma* |
| *Acipenser naccarii* | *Paraconger californiensis* | *Gymnothorax nudivomer* | *Callechelys cliffi* |
| *Acipenser nudiventris* | *Paraconger notialis* | *Gymnothorax panamensis* | *Callechelys eristigma* |
| *Acipenser oxyrinchus* | ***Pseudophichthys*** | *Gymnothorax pictus* | *Callechelys marmorata* |
| *Acipenser sinensis* | *Pseudophichthys splendens* | *Gymnothorax polyuranodon* | ***Dalophis*** |
| *Acipenser stellatus* | ***Rhynchoconger*** | *Gymnothorax richardsonii* | *Dalophis boulengeri* |
| *Acipenser sturio* | *Rhynchoconger flavus* | *Gymnothorax rueppelliae* | *Dalophis imberbis* |
| *Acipenser transmontanus* | *Rhynchoconger guppyi* | *Gymnothorax saxicola* | ***Echelus*** |
| **Huso** | *Rhynchoconger nitens* | *Gymnothorax steindachneri* | *Echelus myrus* |
| *Huso huso* | **Moringuidae** | *Gymnothorax thyrsoideus* | *Echelus uropterus* |
| **Albuliformes** | ***Moringua*** | *Gymnothorax undulatus* | ***Echiophis*** |
| **Albulidae** | *Moringua ferruginea* | *Gymnothorax unicolor* | *Echiophis brunneus* |
| ***Albula*** | *Moringua javanica* | *Gymnothorax zonipectis* | *Echiophis intertinctus* |
| *Albula glossodonta* | *Moringua microchir* | ***Muraena*** | ***Hemerorhinus*** |
| *Albula vulpes* | **Muraenesocidae** | *Muraena argus* | *Hemerorhinus opici* |
| **Anguilliformes** | ***Congresox*** | *Muraena augusti* | ***Ichthyapus*** |
| **Anguillidae** | *Congresox talabon* | *Muraena clepsydra* | *Ichthyapus selachops* |
| ***Anguilla*** | *Congresox talabonoides* | *Muraena helena* | *Ichthyapus vulturis* |
| *Anguilla anguilla* | ***Cynoponticus*** | *Muraena melanotis* | ***Leiuranus*** |
| *Anguilla australis* | *Cynoponticus coniceps* | *Muraena retifera* | *Leiuranus semicinctus* |
| *Anguilla bengalensis* | *Cynoponticus ferox* | *Muraena robusta* | ***Letharchus*** |
| *Anguilla bicolor* | ***Muraenesox*** | ***Pseudechidna*** | *Letharchus rosenblatti* |
| *Anguilla celebesensis* | *Muraenesox cinereus* | *Pseudechidna brummeri* | ***Muraenichthys*** |
| *Anguilla dieffenbachii* | **Muraenidae** | ***Rhinomuraena*** | *Muraenichthys schultzei* |
| *Anguilla japonica* | ***Anarchias*** | *Rhinomuraena quaesita* | *Muraenichthys sibogae* |
| *Anguilla marmorata* | *Anarchias allardicei* | ***Scuticaria*** | ***Myrichthys*** |
| *Anguilla megastoma* | *Anarchias cantonensis* | *Scuticaria tigrina* | *Myrichthys aspetocheiros* |
| *Anguilla mossambica* | *Anarchias galapagensis* | ***Strophidon*** | *Myrichthys breviceps* |
| *Anguilla obscura* | *Anarchias similis* | *Strophidon sathete* | *Myrichthys colubrinus* |
| *Anguilla reinhardtii* | ***Channomuraena*** | ***Uropterygius*** | *Myrichthys maculosus* |
| *Anguilla rostrata* | *Channomuraena vittata* | *Uropterygius concolor* | *Myrichthys ocellatus* |
| **Chlopsidae** | ***Echidna*** | *Uropterygius fuscoguttatus* | *Myrichthys pardalis* |
| ***Chlopsis*** | *Echidna catenata* | *Uropterygius kamar* | *Myrichthys tigrinus* |
| *Chlopsis olokun* | *Echidna leucotaenia* | *Uropterygius macrocephalus* | ***Ophichthus*** |
| ***Kaupichthys*** | *Echidna nebulosa* | *Uropterygius marmoratus* | *Ophichthus apicalis* |
| *Kaupichthys atronasus* | *Echidna nocturna* | *Uropterygius micropterus* | *Ophichthus cephalozona* |
| *Kaupichthys brachychirus* | *Echidna peli* | *Uropterygius nagoensis* | *Ophichthus cruentifer* |
| *Kaupichthys hyoproroides* | *Echidna polyzona* | *Uropterygius polyspilus* | *Ophichthus frontalis* |
| **Colocongridae** | *Echidna unicolor* | *Uropterygius polystictus* | *Ophichthus gomesii* |
| ***Coloconger*** | ***Enchelycore*** | *Uropterygius supraforatus* | *Ophichthus macrochir* |
| Coloconger cadenati | *Enchelycore anatina* | *Uropterygius versutus* | *Ophichthus puncticeps* |
| **Congridae** | *Enchelycore bayeri* | *Uropterygius xanthopterus* | *Ophichthus remiger* |
| **Ariosoma** | *Enchelycore octaviana* | **Nemichthyidae** | *Ophichthus rufus* |
| *Ariosoma balearicum* | *Enchelycore schismatorhynchus* | ***Avocettina*** | ***Ophisurus*** |
| *Ariosoma fasciatum* | ***Enchelynassa*** | *Avocettina infans* | *Ophisurus serpens* |
| *Ariosoma gilberti* | *Enchelynassa canina* | ***Labichthys*** | ***Phaenomonas*** |
| *Ariosoma prorigerum* | ***Gymnomuraena*** | *Labichthys carinatus* | *Phaenomonas cooperae* |
| *Ariosoma scheelei* | *Gymnomuraena zebra* | ***Nemichthys*** | *Phaenomonas pinnata* |
| ***Bathycongrus*** | ***Gymnothorax*** | *Nemichthys curvirostris* | ***Phyllophichthus*** |
| *Bathycongrus bullisi* | *Gymnothorax buroensis* | *Nemichthys larseni* | *Phyllophichthus xenodontus* |
| *Bathycongrus macrurus* | *Gymnothorax castaneus* | *Nemichthys scolopaceus* | ***Pisodonophis*** |
| *Bathycongrus wallacei* | *Gymnothorax castlei* | **Nettastomatidae** | *Pisodonophis semicinctus* |
| ***Bathyuroconger*** | *Gymnothorax conspersus* | ***Facciolella*** | ***Pseudomyrophis*** |
| *Bathyuroconger vicinus* | *Gymnothorax dovii* | *Facciolella oxyrhyncha* | *Pseudomyrophis atlanticus* |
| **Conger** | *Gymnothorax enigmaticus* | ***Nettastoma*** | *Pseudomyrophis micropinna* |
| *Conger cinereus* | *Gymnothorax equatorialis* | *Nettastoma melanurum* | ***Quassiremus*** |
| *Conger conger* | *Gymnothorax fimbriatus* | ***Venefica*** | *Quassiremus nothochir* |
| *Conger myriaster* | *Gymnothorax flavimarginatus* | *Venefica tentaculata* | ***Schismorhynchus*** |
| *Conger oceanicus* | *Gymnothorax funebris* | **Ophichthidae** | *Schismorhynchus labialis* |
| *Conger orbignianus* | *Gymnothorax fuscomaculatus* | ***Aprognathodon*** | ***Schultzidia*** |
| **Diploconger** | *Gymnothorax gracilicauda* | *Aprognathodon platyventris* | *Schultzidia johnstonensis* |
| *Diploconger polystigmatus* | *Gymnothorax hepaticus* | ***Apterichtus*** | *Schultzidia retropinnis* |
| **Gnathophis** | *Gymnothorax javanicus* | *Apterichtus anguiformis* | ***Scolecenchelys*** |
| *Gnathophis cinctus* | *Gymnothorax mareei* | *Apterichtus ansp* | *Scolecenchelys gymnota* |
| *Gnathophis mystax* | *Gymnothorax margaritophorus* | *Apterichtus caecus* | *Scolecenchelys laticaudata* |

**Table S1 (continued).**

| *Scolecenchelys macroptera* | ***Leuresthes*** | *Scopelosaurus hoedti* | **Batrachoidiformes** |
| --- | --- | --- | --- |
| **Serrivomeridae** | *Leuresthes tenuis* | *Scopelosaurus lepidus* | **Batrachoididae** |
| ***Serrivomer*** | ***Membras*** | *Scopelosaurus meadi* | ***Amphichthys*** |
| *Serrivomer beanii* | *Membras martinica* | *Scopelosaurus smithii* | *Amphichthys cryptocentrus* |
| **Synaphobranchidae** | ***Menidia*** | **Omosudidae** | ***Batrachoides*** |
| ***Diastobranchus*** | *Menidia beryllina* | ***Omosudis*** | *Batrachoides liberiensis* |
| *Diastobranchus capensis* | *Menidia menidia* | *Omosudis lowii* | *Batrachoides surinamensis* |
| ***Haptenchelys*** | ***Odontesthes*** | **Paralepididae** | ***Batrachomoeus*** |
| *Haptenchelys texis* | *Odontesthes argentinensis* | ***Arctozenus*** | *Batrachomoeus dubius* |
| ***Histiobranchus*** | *Odontesthes regia* | *Arctozenus risso* | *Batrachomoeus occidentalis* |
| *Histiobranchus bathybius* | **Isonidae** | ***Lestidiops*** | *Batrachomoeus trispinosus* |
| *Histiobranchus bruuni* | ***Iso*** | *Lestidiops affinis* | ***Daector*** |
| ***Ilyophis*** | *Iso hawaiiensis* | *Lestidiops pseudosphyraenoides* | *Daector dowi* |
| *Ilyophis arx* | *Iso natalensis* | *Lestidiops ringens* | *Daector schmitti* |
| *Ilyophis blachei* | **Pseudomugilidae** | *Lestidiops similis* | ***Halobatrachus*** |
| *Ilyophis brunneus* | ***Pseudomugil*** | *Lestidiops sphyrenoides* | *Halobatrachus didactylus* |
| *Ilyophis nigeli* | *Pseudomugil inconspicuus* | ***Lestidium*** | ***Opsanus*** |
| ***Meadia*** | **Aulopiformes** | *Lestidium atlanticum* | *Opsanus beta* |
| *Meadia abyssalis* | **Alepisauridae** | *Lestidium nudum* | *Opsanus pardus* |
| ***Simenchelys*** | ***Alepisaurus*** | ***Lestrolepis*** | *Opsanus tau* |
| *Simenchelys parasitica* | *Alepisaurus brevirostris* | *Lestrolepis intermedia* | ***Perulibatrachus*** |
| ***Synaphobranchus*** | *Alepisaurus ferox* | ***Magnisudis*** | *Perulibatrachus elminensis* |
| *Synaphobranchus affinis* | **Anotopteridae** | *Magnisudis atlantica* | ***Porichthys*** |
| *Synaphobranchus brevidorsalis* | ***Anotopterus*** | *Magnisudis prionosa* | *Porichthys analis* |
| *Synaphobranchus calvus* | *Anotopterus pharao* | ***Notolepis*** | *Porichthys ephippiatus* |
| *Synaphobranchus kaupii* | **Aulopidae** | *Notolepis annulata* | *Porichthys greenei* |
| *Synaphobranchus oregoni* | ***Aulopus*** | *Notolepis coatsi* | *Porichthys mimeticus* |
| **Ateleopodiformes** | *Aulopus bajacali* | ***Paralepis*** | *Porichthys myriaster* |
| **Ateleopodidae** | *Aulopus filamentosus* | *Paralepis coregonoides* | *Porichthys notatus* |
| ***Guentherus*** | **Bathysauridae** | ***Stemonosudis*** | *Porichthys plectrodon* |
| *Guentherus altivela* | ***Bathysaurus*** | *Stemonosudis elegans* | *Porichthys porosissimus* |
| ***Ijimaia*** | *Bathysaurus ferox* | ***Sudis*** | **Beloniformes** |
| *Ijimaia loppei* | *Bathysaurus mollis* | *Sudis atrox* | **Belonidae** |
| **Atheriniformes** | **Chlorophthalmidae** | *Sudis hyalina* | ***Ablennes*** |
| **Atherinidae** | ***Chlorophthalmus*** | ***Uncisudis*** | *Ablennes hians* |
| ***Atherina*** | *Chlorophthalmus agassizi* | *Uncisudis advena* | ***Belone*** |
| *Atherina boyeri* | ***Parasudis*** | **Scopelarchidae** | *Belone belone* |
| *Atherina breviceps* | *Parasudis fraserbrunneri* | ***Benthalbella*** | ***Platybelone*** |
| *Atherina hepsetus* | *Parasudis truculenta* | *Benthalbella dentata* | *Platybelone argalus argalus* |
| *Atherina presbyter* | **Evermannellidae** | *Benthalbella elongata* | *Platybelone argalus platyura* |
| ***Atherinason*** | ***Evermannella*** | *Benthalbella macropinna* | ***Strongylura*** |
| *Atherinason hepsetoides* | *Evermannella balbo* | ***Scopelarchoides*** | *Strongylura exilis* |
| ***Atherinomorus*** | **Giganturidae** | *Scopelarchoides nicholsi* | *Strongylura incisa* |
| *Atherinomorus duodecimalis* | ***Gigantura*** | *Scopelarchoides signifer* | *Strongylura leiura* |
| *Atherinomorus endrachtensis* | *Gigantura chuni* | ***Scopelarchus*** | *Strongylura marina* |
| *Atherinomorus insularum* | **Ipnopidae** | *Scopelarchus michaelsarsi* | *Strongylura notata notata* |
| *Atherinomorus lacunosus* | ***Bathymicrops*** | **Synodontidae** | *Strongylura senegalensis* |
| ***Atherinosoma*** | *Bathymicrops regis* | ***Harpadon*** | ***Tylosurus*** |
| *Atherinosoma elongata* | ***Bathypterois*** | *Harpadon nehereus* | *Tylosurus acus acus* |
| *Atherinosoma microstoma* | *Bathypterois atricolor* | ***Saurida*** | *Tylosurus acus melanotus* |
| ***Atherion*** | *Bathypterois dubius* | *Saurida brasiliensis* | *Tylosurus crocodilus* |
| *Atherion elymus* | *Bathypterois grallator* | *Saurida gracilis* | *Tylosurus fodiator* |
| ***Craterocephalus*** | *Bathypterois longifilis* | *Saurida nebulosa* | *Tylosurus pacificus* |
| *Craterocephalus mugiloides* | *Bathypterois longipes* | *Saurida tumbil* | **Exocoetidae** |
| ***Hypoatherina*** | *Bathypterois phenax* | *Saurida undosquamis* | ***Cheilopogon*** |
| *Hypoatherina barnesi* | *Bathypterois quadrifilis* | ***Synodus*** | *Cheilopogon abei* |
| *Hypoatherina ovalaua* | *Bathypterois viridensis* | *Synodus binotatus* | *Cheilopogon agoo* |
| ***Kestratherina*** | ***Bathytyphlops*** | *Synodus dermatogenys* | *Cheilopogon cyanopterus* |
| *Kestratherina esox* | *Bathytyphlops marionae* | *Synodus foetens* | *Cheilopogon furcatus* |
| ***Leptatherina*** | *Bathytyphlops sewelli* | *Synodus intermedius* | *Cheilopogon heterurus* |
| *Leptatherina presbyteroides* | ***Ipnops*** | *Synodus jaculum* | *Cheilopogon katoptron* |
| ***Stenatherina*** | *Ipnops agassizii* | *Synodus lacertinus* | *Cheilopogon melanurus* |
| *Stenatherina panatela* | **Notosudidae** | *Synodus lucioceps* | *Cheilopogon nigricans* |
| **Atherinopsidae** | ***Ahliesaurus*** | *Synodus poeyi* | *Cheilopogon pitcairnensis* |
| ***Atherinella*** | *Ahliesaurus berryi* | *Synodus rubromarmoratus* | *Cheilopogon spilonotopterus* |
| *Atherinella brasiliensis* | *Ahliesaurus brevis* | *Synodus saurus* | ***Cypselurus*** |
| *Atherinella eriarcha* | ***Scopelosaurus*** | *Synodus scituliceps* | *Cypselurus angusticeps* |
| *Atherinella nepenthe* | *Scopelosaurus adleri* | *Synodus sechurae* | *Cypselurus callopterus* |
| ***Atherinops*** | *Scopelosaurus ahlstromi* | *Synodus synodus* | *Cypselurus naresii* |
| *Atherinops affinis* | *Scopelosaurus argenteus* | *Synodus variegatus* | *Cypselurus oligolepis* |
| ***Atherinopsis*** | *Scopelosaurus hamiltoni* | ***Trachinocephalus*** | *Cypselurus opisthopus* |
| *Atherinopsis californiensis* | *Scopelosaurus harryi* | *Trachinocephalus myops* | *Cypselurus poecilopterus* |

**Table S1 (continued).**

| ***Exocoetus*** | *Myripristis kuntee* | *Alosa alosa* | *Opisthonema bulleri* |
| --- | --- | --- | --- |
| *Exocoetus monocirrhus* | *Myripristis leiognathus* | *Alosa chrysochloris* | *Opisthonema libertate* |
| *Exocoetus obtusirostris* | *Myripristis murdjan* | *Alosa fallax* | *Opisthonema medirastre* |
| ***Fodiator*** | *Myripristis pralinia* | *Alosa immaculata* | *Opisthonema oglinum* |
| *Fodiator rostratus* | *Myripristis violacea* | *Alosa mediocris* | ***Pellonula*** |
| ***Hirundichthys*** | *Myripristis vittata* | *Alosa pseudoharengus* | *Pellonula leonensis* |
| *Hirundichthys affinis* | *Myripristis woodsi* | *Alosa sapidissima* | ***Ramnogaster*** |
| *Hirundichthys marginatus* | ***Neoniphon*** | *Alosa tanaica* | *Ramnogaster arcuata* |
| *Hirundichthys oxycephalus* | *Neoniphon argenteus* | ***Amblygaster*** | ***Sardina*** |
| *Hirundichthys rondeletii* | *Neoniphon aurolineatus* | *Amblygaster clupeoides* | *Sardina pilchardus* |
| *Hirundichthys speculiger* | *Neoniphon marianus* | *Amblygaster leiogaster* | ***Sardinella*** |
| ***Parexocoetus*** | *Neoniphon opercularis* | *Amblygaster sirm* | *Sardinella albella* |
| *Parexocoetus brachypterus* | *Neoniphon sammara* | ***Anodontostoma*** | *Sardinella aurita* |
| *Parexocoetus mento* | ***Ostichthys*** | *Anodontostoma chacunda* | *Sardinella brachysoma* |
| ***Prognichthys*** | *Ostichthys archiepiscopus* | *Anodontostoma selangkat* | *Sardinella brasiliensis* |
| *Prognichthys occidentalis* | *Ostichthys trachypoma* | *Anodontostoma thailandiae* | *Sardinella fijiense* |
| *Prognichthys tringa* | ***Plectrypops*** | ***Brevoortia*** | *Sardinella fimbriata* |
| **Hemiramphidae** | *Plectrypops lima* | *Brevoortia aurea* | *Sardinella gibbosa* |
| ***Arrhamphus*** | ***Sargocentron*** | *Brevoortia gunteri* | *Sardinella hualiensis* |
| *Arrhamphus sclerolepis* | *Sargocentron caudimaculatum* | *Brevoortia patronus* | *Sardinella jussieu* |
| ***Hemiramphus*** | *Sargocentron cornutum* | *Brevoortia pectinata* | *Sardinella lemuru* |
| *Hemiramphus balao* | *Sargocentron coruscum* | *Brevoortia smithi* | *Sardinella longiceps* |
| *Hemiramphus brasiliensis* | *Sargocentron diadema* | *Brevoortia tyrannus* | *Sardinella maderensis* |
| *Hemiramphus far* | *Sargocentron hastatum* | ***Clupanodon*** | *Sardinella marquesensis* |
| *Hemiramphus robustus* | *Sargocentron iota* | *Clupanodon thrissa* | *Sardinella melanura* |
| ***Hyporhamphus*** | *Sargocentron ittodai* | ***Clupea*** | *Sardinella richardsoni* |
| *Hyporhamphus acutus* | *Sargocentron melanospilos* | *Clupea harengus* | *Sardinella rouxi* |
| *Hyporhamphus australis* | *Sargocentron microstoma* | *Clupea pallasii pallasii* | *Sardinella sindensis* |
| *Hyporhamphus gilli* | *Sargocentron punctatissimum* | ***Clupeonella*** | *Sardinella zunasi* |
| *Hyporhamphus melanochir* | *Sargocentron rubrum* | *Clupeonella cultriventris* | ***Spratelloides*** |
| *Hyporhamphus picarti* | *Sargocentron spiniferum* | ***Dorosoma*** | *Spratelloides delicatulus* |
| *Hyporhamphus sajori* | *Sargocentron suborbitale* | *Dorosoma cepedianum* | *Spratelloides lewisi* |
| *Hyporhamphus unifasciatus* | *Sargocentron tiere* | *Dorosoma petenense* | *Spratelloides robustus* |
| ***Oxyporhamphus*** | *Sargocentron tiereoides* | ***Escualosa*** | ***Sprattus*** |
| *Oxyporhamphus micropterus* | *Sargocentron violaceum* | *Escualosa thoracata* | *Sprattus antipodum* |
| **Scomberesocidae** | **Monocentridae** | ***Ethmalosa*** | *Sprattus fuegensis* |
| ***Cololabis*** | ***Cleidopus*** | *Ethmalosa fimbriata* | *Sprattus muelleri* |
| *Cololabis saira* | *Cleidopus gloriamaris* | ***Harengula*** | *Sprattus novaehollandiae* |
| ***Scomberesox*** | **Trachichthyidae** | *Harengula clupeola* | *Sprattus sprattus* |
| *Scomberesox saurus* | ***Gephyroberyx*** | *Harengula humeralis* | ***Tenualosa*** |
| **Beryciformes** | *Gephyroberyx darwinii* | *Harengula jaguana* | *Tenualosa ilisha* |
| **Anomalopidae** | ***Hoplostethus*** | *Harengula thrissina* | *Tenualosa macrura* |
| ***Anomalops*** | *Hoplostethus atlanticus* | ***Herklotsichthys*** | *Tenualosa reevesii* |
| *Anomalops katoptron* | *Hoplostethus cadenati* | *Herklotsichthys blackburni* | *Tenualosa toli* |
| ***Photoblepharon*** | *Hoplostethus crassispinus* | *Herklotsichthys castelnaui* | **Dussumieriidae** |
| *Photoblepharon palpebratum* | *Hoplostethus melanopeza* | *Herklotsichthys dispilonotus* | ***Dussumieria*** |
| **Anoplogastridae** | **Cetomimiformes** | *Herklotsichthys gotoi* | *Dussumieria acuta* |
| ***Anoplogaster*** | **Cetomimidae** | *Herklotsichthys koningsbergeri* | *Dussumieria elopsoides* |
| *Anoplogaster cornuta* | ***Cetostoma*** | *Herklotsichthys lippa* | ***Etrumeus*** |
| **Berycidae** | *Cetostoma regani* | *Herklotsichthys lossei* | *Etrumeus whiteheadi* |
| ***Beryx*** | ***Danacetichthys*** | *Herklotsichthys punctatus* | **Engraulidae** |
| *Beryx decadactylus* | *Danacetichthys galathenus* | *Herklotsichthys quadrimaculatus* | ***Anchoa*** |
| *Beryx splendens* | ***Ditropichthys*** | *Herklotsichthys spilurus* | *Anchoa argentivittata* |
| ***Centroberyx*** | *Ditropichthys storeri* | ***Hilsa*** | *Anchoa cayorum* |
| *Centroberyx affinis* | ***Eutaeniophorus*** | *Hilsa kelee* | *Anchoa chamensis* |
| *Centroberyx gerrardi* | *Eutaeniophorus festivus* | ***Hyperlophus*** | *Anchoa colonensis* |
| **Diretmidae** | ***Gyrinomimus*** | *Hyperlophus vittatus* | *Anchoa compressa* |
| ***Diretmichthys*** | *Gyrinomimus bruuni* | ***Jenkinsia*** | *Anchoa cubana* |
| *Diretmichthys parini* | *Gyrinomimus grahami* | *Jenkinsia lamprotaenia* | *Anchoa curta* |
| ***Diretmus*** | ***Parataeniophorus*** | *Jenkinsia majua* | *Anchoa delicatissima* |
| *Diretmus argenteus* | *Parataeniophorus gulosus* | *Jenkinsia stolifera* | *Anchoa eigenmannia* |
| **Holocentridae** | **Rondeletiidae** | ***Konosirus*** | *Anchoa exigua* |
| ***Holocentrus*** | ***Rondeletia*** | *Konosirus punctatus* | *Anchoa filifera* |
| *Holocentrus adscensionis* | *Rondeletia loricata* | ***Lile*** | *Anchoa helleri* |
| *Holocentrus rufus* | **Clupeiformes** | *Lile piquitinga* | *Anchoa hepsetus* |
| ***Myripristis*** | **Chirocentridae** | *Lile stolifera* | *Anchoa ischana* |
| *Myripristis adusta* | ***Chirocentrus*** | ***Nematalosa*** | *Anchoa lamprotaenia* |
| *Myripristis amaena* | *Chirocentrus dorab* | *Nematalosa galatheae* | *Anchoa lucida* |
| *Myripristis berndti* | *Chirocentrus nudus* | *Nematalosa japonica* | *Anchoa lyolepis* |
| *Myripristis chryseres* | **Clupeidae** | *Nematalosa nasus* | *Anchoa mitchilli* |
| *Myripristis hexagona* | ***Alosa*** | ***Opisthonema*** | *Anchoa nasus* |
| *Myripristis jacobus* | *Alosa aestivalis* | *Opisthonema berlangai* | *Anchoa panamensis* |

**Table S1 (continued).**

| *Anchoa parva* | *Thryssa setirostris* | ***Gadus*** | *Coelorinchus karrerae* |
| --- | --- | --- | --- |
| *Anchoa pectoralis* | *Thryssa spinidens* | *Gadus macrocephalus* | *Coelorinchus kishinouyei* |
| *Anchoa scofieldi* | *Thryssa vitrirostris* | *Gadus morhua* | *Coelorinchus labiatus* |
| *Anchoa spinifer* | **Pristigasteridae** | ***Melanogrammus*** | *Coelorinchus macrochir* |
| *Anchoa starksi* | ***Chirocentrodon*** | *Melanogrammus aeglefinus* | *Coelorinchus marinii* |
| *Anchoa tricolor* | *Chirocentrodon bleekerianus* | ***Merlangius*** | *Coelorinchus mirus* |
| *Anchoa walkeri* | ***Ilisha*** | *Merlangius merlangus* | *Coelorinchus mycterismus* |
| ***Anchovia*** | *Ilisha africana* | ***Microgadus*** | *Coelorinchus occa* |
| *Anchovia clupeoides* | *Ilisha elongata* | *Microgadus proximus* | *Coelorinchus oliverianus* |
| *Anchovia macrolepidota* | *Ilisha filigera* | *Microgadus tomcod* | *Coelorinchus parallelus* |
| ***Anchoviella*** | *Ilisha fuerthii* | ***Micromesistius*** | *Coelorinchus scaphopsis* |
| *Anchoviella brevirostris* | *Ilisha kampeni* | *Micromesistius australis* | *Coelorinchus supernasutus* |
| *Anchoviella lepidentostole* | *Ilisha macrogaster* | *Micromesistius poutassou* | *Coelorinchus thurla* |
| *Anchoviella perfasciata* | *Ilisha megaloptera* | ***Pollachius*** | *Coelorinchus ventrilux* |
| ***Cetengraulis*** | *Ilisha melastoma* | *Pollachius pollachius* | ***Coryphaenoides*** |
| *Cetengraulis edentulus* | *Ilisha sirishai* | *Pollachius virens* | *Coryphaenoides acrolepis* |
| *Cetengraulis mysticetus* | *Ilisha striatula* | ***Raniceps*** | *Coryphaenoides anguliceps* |
| ***Coilia*** | ***Neoopisthopterus*** | *Raniceps raninus* | *Coryphaenoides ariommus* |
| *Coilia dussumieri* | *Neoopisthopterus tropicus* | ***Trisopterus*** | *Coryphaenoides armatus* |
| *Coilia grayii* | ***Odontognathus*** | *Trisopterus esmarkii* | *Coryphaenoides boops* |
| *Coilia nasus* | *Odontognathus mucronatus* | *Trisopterus luscus* | *Coryphaenoides bucephalus* |
| *Coilia neglecta* | ***Opisthopterus*** | *Trisopterus minutus* | *Coryphaenoides bulbiceps* |
| *Coilia reynaldi* | *Opisthopterus dovii* | **Lotidae** | *Coryphaenoides capito* |
| ***Encrasicholina*** | *Opisthopterus tardoore* | ***Brosme*** | *Coryphaenoides carminifer* |
| *Encrasicholina devisi* | *Opisthopterus valenciennesi* | *Brosme brosme* | *Coryphaenoides cinereus* |
| *Encrasicholina heteroloba* | ***Pellona*** | ***Ciliata*** | *Coryphaenoides delsolari* |
| *Encrasicholina punctifer* | *Pellona ditchela* | *Ciliata mustela* | *Coryphaenoides ferrieri* |
| *Encrasicholina purpurea* | *Pellona harroweri* | *Ciliata septentrionalis* | *Coryphaenoides filicauda* |
| ***Engraulis*** | ***Pliosteostoma*** | ***Enchelyopus*** | *Coryphaenoides filifer* |
| *Engraulis anchoita* | *Pliosteostoma lutipinnis* | *Enchelyopus cimbrius* | *Coryphaenoides guentheri* |
| *Engraulis australis* | ***Raconda*** | ***Gaidropsarus*** | *Coryphaenoides lecointei* |
| *Engraulis encrasicolus* | *Raconda russeliana* | *Gaidropsarus argentatus* | *Coryphaenoides leptolepis* |
| *Engraulis eurystole* | **Cyprinodontiformes** | *Gaidropsarus biscayensis* | *Coryphaenoides longifilis* |
| *Engraulis japonicus* | **Cyprinodontidae** | *Gaidropsarus ensis* | *Coryphaenoides marginatus* |
| *Engraulis mordax* | ***Aphanius*** | *Gaidropsarus granti* | *Coryphaenoides mediterraneus* |
| *Engraulis ringens* | *Aphanius fasciatus* | *Gaidropsarus guttatus* | *Coryphaenoides mexicanus* |
| ***Lycengraulis*** | **Fundulidae** | *Gaidropsarus macrophthalmus* | *Coryphaenoides rudis* |
| *Lycengraulis grossidens* | ***Fundulus*** | *Gaidropsarus mediterraneus* | *Coryphaenoides rupestris* |
| *Lycengraulis poeyi* | *Fundulus confluentus* | *Gaidropsarus vulgaris* | *Coryphaenoides serrulatus* |
| ***Setipinna*** | *Fundulus heteroclitus* | ***Molva*** | *Coryphaenoides subserrulatus* |
| *Setipinna breviceps* | *Fundulus majalis* | *Molva dypterygia* | *Coryphaenoides zaniophorus* |
| *Setipinna melanochir* | *Fundulus similis* | *Molva macrophthalma* | ***Cynomacrurus*** |
| *Setipinna taty* | ***Lucania*** | *Molva molva* | *Cynomacrurus piriei* |
| *Setipinna tenuifilis* | *Lucania parva* | **Macrouridae** | ***Echinomacrurus*** |
| ***Stolephorus*** | **Elopiformes** | ***Albatrossia*** | *Echinomacrurus mollis* |
| *Stolephorus advenus* | **Elopidae** | *Albatrossia pectoralis* | ***Gadomus*** |
| *Stolephorus andhraensis* | ***Elops*** | ***Bathygadus*** | *Gadomus arcuatus* |
| *Stolephorus apiensis* | *Elops hawaiensis* | *Bathygadus macrops* | *Gadomus longifilis* |
| *Stolephorus baganensis* | *Elops lacerta* | *Bathygadus melanobranchus* | ***Hymenocephalus*** |
| *Stolephorus brachycephalus* | *Elops machnata* | ***Coelorinchus*** | *Hymenocephalus italicus* |
| *Stolephorus carpentariae* | *Elops saurus* | *Coelorinchus acanthiger* | ***Lepidorhynchus*** |
| *Stolephorus chinensis* | **Megalopidae** | *Coelorinchus aconcagua* | *Lepidorhynchus denticulatus* |
| *Stolephorus commersonnii* | ***Megalops*** | *Coelorinchus anatirostris* | ***Lucigadus*** |
| *Stolephorus dubiosus* | *Megalops atlanticus* | *Coelorinchus argentatus* | *Lucigadus nigromaculatus* |
| *Stolephorus holodon* | *Megalops cyprinoides* | *Coelorinchus argus* | ***Macrourus*** |
| *Stolephorus indicus* | **Gadiformes** | *Coelorinchus aspercephalus* | *Macrourus berglax* |
| *Stolephorus insularis* | **Bregmacerotidae** | *Coelorinchus australis* | *Macrourus carinatus* |
| *Stolephorus tri* | ***Bregmaceros*** | *Coelorinchus biclinozonalis* | *Macrourus holotrachys* |
| *Stolephorus waitei* | *Bregmaceros bathymaster* | *Coelorinchus caelorhincus* | *Macrourus whitsoni* |
| ***Thryssa*** | *Bregmaceros japonicus* | *Coelorinchus canus* | ***Malacocephalus*** |
| *Thryssa aestuaria* | *Bregmaceros mcclellandi* | *Coelorinchus caribbaeus* | *Malacocephalus laevis* |
| *Thryssa baelama* | **Gadidae** | *Coelorinchus celaenostomus* | *Malacocephalus occidentalis* |
| *Thryssa brevicauda* | ***Arctogadus*** | *Coelorinchus charius* | ***Mataeocephalus*** |
| *Thryssa dayi* | *Arctogadus glacialis* | *Coelorinchus chilensis* | *Mataeocephalus acipenserinus* |
| *Thryssa dussumieri* | ***Boreogadus*** | *Coelorinchus denticulatus* | *Mataeocephalus tenuicauda* |
| *Thryssa encrasicholoides* | *Boreogadus saida* | *Coelorinchus fasciatus* | ***Mesobius*** |
| *Thryssa gautamiensis* | ***Eleginus*** | *Coelorinchus formosanus* | *Mesobius berryi* |
| *Thryssa hamiltonii* | *Eleginus gracilis* | *Coelorinchus horribilis* | ***Nezumia*** |
| *Thryssa kammalensis* | *Eleginus nawaga* | *Coelorinchus innotabilis* | *Nezumia aequalis* |
| *Thryssa malabarica* | ***Gadiculus*** | *Coelorinchus japonicus* | *Nezumia atlantica* |
| *Thryssa mystax* | *Gadiculus argenteus* | *Coelorinchus kaiyomaru* | *Nezumia bairdii* |
| *Thryssa purava* | *Gadiculus thori* | *Coelorinchus kamoharai* | *Nezumia brevibarbata* |

**Table S1 (continued).**

| *Nezumia condylura* | *Eeyorius hutchinsi* | ***Hypoptychus*** | *Antennarius commerson* |
| --- | --- | --- | --- |
| *Nezumia convergens* | ***Gadella*** | *Hypoptychus dybowskii* | *Antennarius hispidus* |
| *Nezumia duodecim* | *Gadella brocca* | **Pegasidae** | *Antennarius maculatus* |
| *Nezumia kapala* | *Gadella imberbis* | ***Eurypegasus*** | *Antennarius multiocellatus* |
| *Nezumia latirostrata* | *Gadella maraldi* | *Eurypegasus draconis* | *Antennarius pardalis* |
| *Nezumia liolepis* | ***Halargyreus*** | *Eurypegasus papilio* | *Antennarius pictus* |
| *Nezumia loricata* | *Halargyreus johnsonii* | ***Pegasus*** | *Antennarius randalli* |
| *Nezumia micronychodon* | ***Laemonema*** | *Pegasus lancifer* | *Antennarius striatus* |
| *Nezumia milleri* | *Laemonema barbatulum* | *Pegasus laternarius* | ***Antennatus*** |
| *Nezumia orbitalis* | *Laemonema goodebeanorum* | *Pegasus volitans* | *Antennatus analis* |
| *Nezumia parini* | *Laemonema laureysi* | **Gobiesociformes** | *Antennatus coccineus* |
| *Nezumia propinqua* | *Laemonema longipes* | **Gobiesocidae** | *Antennatus dorehensis* |
| *Nezumia proxima* | *Laemonema verecundum* | ***Apletodon*** | *Antennatus nummifer* |
| *Nezumia pudens* | *Laemonema yarrellii* | *Apletodon dentatus* | *Antennatus rosaceus* |
| *Nezumia pulchella* | ***Lepidion*** | *Apletodon incognitus* | *Antennatus sanguineus* |
| *Nezumia sclerorhynchus* | *Lepidion ensiferus* | ***Diplecogaster*** | *Antennatus strigatus* |
| *Nezumia stelgidolepis* | *Lepidion eques* | *Diplecogaster bimaculata* | *Antennatus tuberosus* |
| ***Sphagemacrurus*** | *Lepidion guentheri* | ***Gobiesox*** | ***Echinophryne*** |
| *Sphagemacrurus grenadae* | *Lepidion lepidion* | *Gobiesox maeandricus* | *Echinophryne crassispina* |
| *Sphagemacrurus hirundo* | ***Mora*** | *Gobiesox strumosus* | *Echinophryne reynoldsi* |
| ***Trachonurus*** | *Mora moro* | ***Gouania*** | ***Fowlerichthys*** |
| *Trachonurus gagates* | ***Notophycis*** | *Gouania willdenowi* | *Fowlerichthys avalonis* |
| *Trachonurus robinsi* | *Notophycis marginata* | ***Lepadichthys*** | *Fowlerichthys ocellatus* |
| *Trachonurus sulcatus* | ***Physiculus*** | *Lepadichthys caritus* | *Fowlerichthys radiosus* |
| ***Trachyrincus*** | *Physiculus dalwigki* | *Lepadichthys minor* | ***Histrio*** |
| *Trachyrincus helolepis* | *Physiculus huloti* | ***Lepadogaster*** | *Histrio histrio* |
| *Trachyrincus scabrus* | *Physiculus japonicus* | *Lepadogaster candolii* | ***Kuiterichthys*** |
| *Trachyrincus villegai* | *Physiculus nematopus* | *Lepadogaster lepadogaster* | *Kuiterichthys furcipilis* |
| ***Ventrifossa*** | *Physiculus rastrelliger* | ***Rimicola*** | ***Lophiocharon*** |
| *Ventrifossa atherodon* | ***Pseudophycis*** | *Rimicola muscarum* | *Lophiocharon trisignatus* |
| *Ventrifossa ctenomelas* | *Pseudophycis bachus* | **Gonorynchiformes** | ***Phyllophryne*** |
| *Ventrifossa divergens* | *Pseudophycis barbata* | **Chanidae** | *Phyllophryne scortea* |
| *Ventrifossa garmani* | *Pseudophycis breviuscula* | ***Chanos*** | ***Rhycherus*** |
| *Ventrifossa macropogon* | ***Salilota*** | *Chanos chanos* | *Rhycherus filamentosus* |
| *Ventrifossa misakia* | *Salilota australis* | **Gonorynchidae** | ***Tathicarpus*** |
| *Ventrifossa mucocephalus* | ***Tripterophycis*** | ***Gonorynchus*** | *Tathicarpus butleri* |
| *Ventrifossa nasuta* | *Tripterophycis gilchristi* | *Gonorynchus forsteri* | **Brachionichthyidae** |
| *Ventrifossa nigrodorsalis* | **Muraenolepididae** | *Gonorynchus gonorynchus* | ***Brachionichthys*** |
| *Ventrifossa petersonii* | ***Muraenolepis*** | *Gonorynchus greyi* | *Brachionichthys hirsutus* |
| **Melanonidae** | *Muraenolepis marmorata* | **Lampriformes** | **Centrophrynidae** |
| ***Melanonus*** | *Muraenolepis microps* | **Lampridae** | ***Centrophryne*** |
| *Melanonus gracilis* | *Muraenolepis orangiensis* | ***Lampris*** | *Centrophryne spinulosa* |
| *Melanonus zugmayeri* | ***Notomuraenobathys*** | *Lampris guttatus* | **Ceratiidae** |
| **Merlucciidae** | *Notomuraenobathys microcephalus* | *Lampris immaculatus* | ***Ceratias*** |
| ***Lyconus*** | **Phycidae** | **Lophotidae** | *Ceratias holboelli* |
| *Lyconus brachycolus* | ***Phycis*** | ***Lophotus*** | *Ceratias tentaculatus* |
| ***Macruronus*** | *Phycis blennoides* | *Lophotus lacepede* | ***Cryptopsaras*** |
| *Macruronus capensis* | *Phycis chesteri* | **Radiicephalidae** | *Cryptopsaras couesii* |
| *Macruronus magellanicus* | *Phycis phycis* | ***Radiicephalus*** | **Chaunacidae** |
| *Macruronus novaezelandiae* | ***Urophycis*** | *Radiicephalus elongatus* | ***Chaunacops*** |
| ***Merluccius*** | *Urophycis brasiliensis* | **Regalecidae** | *Chaunacops melanostomus* |
| *Merluccius albidus* | *Urophycis chuss* | ***Agrostichthys*** | ***Chaunax*** |
| *Merluccius angustimanus* | *Urophycis cirrata* | *Agrostichthys parkeri* | *Chaunax abei* |
| *Merluccius australis* | *Urophycis earllii* | ***Regalecus*** | *Chaunax endeavouri* |
| *Merluccius bilinearis* | *Urophycis floridana* | *Regalecus glesne* | *Chaunax fimbriatus* |
| *Merluccius capensis* | *Urophycis regia* | **Stylephoridae** | *Chaunax nebulosus* |
| *Merluccius gayi gayi* | *Urophycis tenuis* | ***Stylephorus*** | *Chaunax stigmaeus* |
| *Merluccius hubbsi* | **Gasterosteiformes** | *Stylephorus chordatus* | *Chaunax suttkusi* |
| *Merluccius merluccius* | **Aulorhynchidae** | **Trachipteridae** | **Gigantactinidae** |
| *Merluccius paradoxus* | ***Aulorhynchus*** | ***Desmodema*** | ***Gigantactis*** |
| *Merluccius polli* | *Aulorhynchus flavidus* | *Desmodema lorum* | *Gigantactis vanhoeffeni* |
| *Merluccius productus* | **Gasterosteidae** | *Desmodema polystictum* | **Linophrynidae** |
| *Merluccius senegalensis* | ***Apeltes*** | ***Trachipterus*** | ***Borophryne*** |
| ***Steindachneria*** | *Apeltes quadracus* | *Trachipterus altivelis* | *Borophryne apogon* |
| *Steindachneria argentea* | ***Gasterosteus*** | *Trachipterus arcticus* | **Lophiidae** |
| **Moridae** | *Gasterosteus aculeatus* | *Trachipterus trachypterus* | ***Lophiodes*** |
| ***Antimora*** | *Gasterosteus wheatlandi* | **Lophiiformes** | *Lophiodes caulinaris* |
| *Antimora microlepis* | ***Pungitius*** | **Antennariidae** | *Lophiodes insidiator* |
| *Antimora rostrata* | *Pungitius pungitius* | ***Allenichthys*** | *Lophiodes kempi* |
| ***Auchenoceros*** | ***Spinachia*** | *Allenichthys glauerti* | *Lophiodes monodi* |
| *Auchenoceros punctatus* | *Spinachia spinachia* | ***Antennarius*** | *Lophiodes reticulatus* |
| ***Eeyorius*** | **Hypoptychidae** | *Antennarius biocellatus* | *Lophiodes spilurus* |

**Table S1 (continued).**

| ***Lophius*** | ***Neochelon*** | *Electrona carlsbergi* | *Nannobrachium atrum* |
| --- | --- | --- | --- |
| *Lophius americanus* | *Neochelon falcipinnis* | *Electrona paucirastra* | *Nannobrachium cuprarium* |
| *Lophius budegassa* | ***Neomyxus*** | *Electrona risso* | *Nannobrachium hawaiiensis* |
| *Lophius gastrophysus* | *Neomyxus leuciscus* | *Electrona subaspera* | *Nannobrachium idostigma* |
| *Lophius piscatorius* | ***Oedalechilus*** | ***Gonichthys*** | *Nannobrachium lineatum* |
| *Lophius vaillanti* | *Oedalechilus labeo* | *Gonichthys barnesi* | *Nannobrachium regale* |
| *Lophius vomerinus* | ***Osteomugil*** | *Gonichthys cocco* | *Nannobrachium ritteri* |
| **Melanocetidae** | *Osteomugil engeli* | *Gonichthys tenuiculus* | ***Notolychnus*** |
| ***Melanocetus*** | ***Planiliza*** | ***Gymnoscopelus*** | *Notolychnus valdiviae* |
| *Melanocetus johnsonii* | *Planiliza alata* | *Gymnoscopelus bolini* | ***Notoscopelus*** |
| *Melanocetus murrayi* | *Planiliza haematocheila* | *Gymnoscopelus braueri* | *Notoscopelus bolini* |
| *Melanocetus polyactis* | *Planiliza macrolepis* | *Gymnoscopelus fraseri* | *Notoscopelus caudispinosus* |
| **Ogcocephalidae** | *Planiliza melinopterus* | *Gymnoscopelus hintonoides* | *Notoscopelus elongatus* |
| ***Halieutaea*** | *Planiliza subviridis* | *Gymnoscopelus nicholsi* | *Notoscopelus japonicus* |
| *Halieutaea brevicauda* | ***Plicomugil*** | *Gymnoscopelus opisthopterus* | *Notoscopelus kroyeri* |
| ***Halieutichthys*** | *Plicomugil labiosus* | *Gymnoscopelus piabilis* | *Notoscopelus resplendens* |
| *Halieutichthys aculeatus* | ***Pseudomyxus*** | ***Hintonia*** | ***Parvilux*** |
| ***Ogcocephalus*** | *Pseudomyxus capensis* | *Hintonia candens* | *Parvilux ingens* |
| *Ogcocephalus corniger* | **Myctophiformes** | ***Hygophum*** | ***Protomyctophum*** |
| *Ogcocephalus cubifrons* | **Myctophidae** | *Hygophum atratum* | *Protomyctophum andriashevi* |
| *Ogcocephalus radiatus* | ***Benthosema*** | *Hygophum benoiti* | *Protomyctophum arcticum* |
| *Ogcocephalus rostellum* | *Benthosema fibulatum* | *Hygophum hanseni* | *Protomyctophum beckeri* |
| *Ogcocephalus vespertilio* | *Benthosema glaciale* | *Hygophum hygomii* | *Protomyctophum bolini* |
| **Oneirodidae** | *Benthosema panamense* | ***Krefftichthys*** | *Protomyctophum choriodon* |
| ***Bertella*** | *Benthosema suborbitale* | *Krefftichthys anderssoni* | *Protomyctophum crockeri* |
| *Bertella idiomorpha* | ***Bolinichthys*** | ***Lampadena*** | *Protomyctophum gemmatum* |
| ***Chaenophryne*** | *Bolinichthys distofax* | *Lampadena anomala* | *Protomyctophum luciferum* |
| *Chaenophryne draco* | *Bolinichthys indicus* | *Lampadena atlantica* | *Protomyctophum normani* |
| *Chaenophryne longiceps* | *Bolinichthys longipes* | *Lampadena chavesi* | *Protomyctophum parallelum* |
| *Chaenophryne melanorhabdus* | *Bolinichthys photothorax* | *Lampadena luminosa* | *Protomyctophum subparallelum* |
| ***Oneirodes*** | *Bolinichthys supralateralis* | *Lampadena notialis* | *Protomyctophum tenisoni* |
| *Oneirodes acanthias* | ***Centrobranchus*** | *Lampadena speculigera* | *Protomyctophum thompsoni* |
| *Oneirodes bulbosus* | *Centrobranchus nigroocellatus* | *Lampadena urophaos* | ***Scopelopsis*** |
| *Oneirodes clarkei* | ***Ceratoscopelus*** | ***Lampanyctodes*** | *Scopelopsis multipunctatus* |
| *Oneirodes kreffti* | *Ceratoscopelus townsendi* | *Lampanyctodes hectoris* | ***Stenobrachius*** |
| *Oneirodes macronema* | *Ceratoscopelus warmingii* | ***Lampanyctus*** | *Stenobrachius leucopsarus* |
| *Oneirodes notius* | ***Diaphus*** | *Lampanyctus alatus* | *Stenobrachius nannochir* |
| *Oneirodes thompsoni* | *Diaphus adenomus* | *Lampanyctus australis* | ***Symbolophorus*** |
| **Mugiliformes** | *Diaphus anderseni* | *Lampanyctus crocodilus* | *Symbolophorus boops* |
| **Mugilidae** | *Diaphus antonbruuni* | *Lampanyctus festivus* | *Symbolophorus californiensis* |
| ***Agonostomus*** | *Diaphus bertelseni* | *Lampanyctus intricarius* | *Symbolophorus kreffti* |
| *Agonostomus monticola* | *Diaphus brachycephalus* | *Lampanyctus lepidolychnus* | *Symbolophorus veranyi* |
| ***Aldrichetta*** | *Diaphus chrysorhynchus* | *Lampanyctus macdonaldi* | ***Taaningichthys*** |
| *Aldrichetta forsteri* | *Diaphus diademophilus* | *Lampanyctus macropterus* | *Taaningichthys bathyphilus* |
| ***Chaenomugil*** | *Diaphus dumerilii* | *Lampanyctus nobilis* | *Taaningichthys minimus* |
| *Chaenomugil proboscideus* | *Diaphus effulgens* | *Lampanyctus pusillus* | *Taaningichthys paurolychnus* |
| ***Chelon*** | *Diaphus fragilis* | *Lampanyctus steinbecki* | ***Tarletonbeania*** |
| *Chelon auratus* | *Diaphus fulgens* | ***Lampichthys*** | *Tarletonbeania crenularis* |
| *Chelon dumerili* | *Diaphus garmani* | *Lampichthys procerus* | ***Triphoturus*** |
| *Chelon labrosus* | *Diaphus holti* | ***Lepidophanes*** | *Triphoturus mexicanus* |
| *Chelon planiceps* | *Diaphus hudsoni* | *Lepidophanes guentheri* | *Triphoturus nigrescens* |
| *Chelon ramada* | *Diaphus lucidus* | ***Lobianchia*** | **Neoscopelidae** |
| *Chelon richardsonii* | *Diaphus luetkeni* | *Lobianchia dofleini* | ***Neoscopelus*** |
| *Chelon saliens* | *Diaphus malayanus* | *Lobianchia gemellarii* | *Neoscopelus macrolepidotus* |
| ***Crenimugil*** | *Diaphus megalops* | ***Loweina*** | *Neoscopelus microchir* |
| *Crenimugil buchanani* | *Diaphus metopoclampus* | *Loweina rara* | ***Scopelengys*** |
| *Crenimugil crenilabis* | *Diaphus mollis* | ***Metelectrona*** | *Scopelengys tristis* |
| *Crenimugil heterocheilos* | *Diaphus perspicillatus* | *Metelectrona ventralis* | **Notacanthiformes** |
| *Crenimugil seheli* | *Diaphus phillipsi* | ***Myctophum*** | **Halosauridae** |
| ***Ellochelon*** | *Diaphus problematicus* | *Myctophum affine* | ***Aldrovandia*** |
| *Ellochelon vaigiensis* | *Diaphus rafinesquii* | *Myctophum asperum* | *Aldrovandia gracilis* |
| ***Gracilimugil*** | *Diaphus schmidti* | *Myctophum aurolaternatum* | *Aldrovandia oleosa* |
| *Gracilimugil argenteus* | *Diaphus splendidus* | *Myctophum fissunovi* | *Aldrovandia rostrata* |
| ***Mugil*** | *Diaphus subtilis* | *Myctophum lychnobium* | ***Halosauropsis*** |
| *Mugil cephalus* | *Diaphus taaningi* | *Myctophum nitidulum* | *Halosauropsis macrochir* |
| *Mugil curema* | *Diaphus termophilus* | *Myctophum obtusirostre* | ***Halosaurus*** |
| *Mugil curvidens* | *Diaphus theta* | *Myctophum phengodes* | *Halosaurus johnsonianus* |
| *Mugil gaimardianus* | ***Diogenichthys*** | *Myctophum punctatum* | *Halosaurus ovenii* |
| *Mugil incilis* | *Diogenichthys atlanticus* | *Myctophum selenops* | **Notacanthidae** |
| *Mugil liza* | *Diogenichthys panurgus* | *Myctophum spinosum* | ***Lipogenys*** |
| *Mugil thoburni* | ***Electrona*** | ***Nannobrachium*** | *Lipogenys gillii* |
| *Mugil trichodon* | *Electrona antarctica* | *Nannobrachium achirus* | ***Notacanthus*** |

**Table S1 (continued).**

| *Notacanthus bonaparte* | ***Lepophidium*** | ***Einara*** | *Nansenia atlantica* |
| --- | --- | --- | --- |
| *Notacanthus chemnitzii* | *Lepophidium aporrhox* | *Einara edentula* | *Nansenia candida* |
| ***Polyacanthonotus*** | *Lepophidium brevibarbe* | *Einara macrolepis* | *Nansenia crassa* |
| *Polyacanthonotus africanus* | *Lepophidium collettei* | ***Herwigia*** | *Nansenia groenlandica* |
| *Polyacanthonotus rissoanus* | *Lepophidium crossotum* | *Herwigia kreffti* | *Nansenia oblita* |
| **Ophidiiformes** | *Lepophidium cultratum* | ***Leptoderma*** | *Nansenia tenera* |
| **Aphyonidae** | *Lepophidium entomelan* | *Leptoderma macrops* | **Opisthoproctidae** |
| ***Nybelinella*** | *Lepophidium gilmorei* | ***Mirognathus*** | ***Bathylychnops*** |
| *Nybelinella erikssoni* | *Lepophidium inca* | *Mirognathus normani* | *Bathylychnops exilis* |
| **Bythitidae** | *Lepophidium jeannae* | ***Narcetes*** | ***Dolichopteroides*** |
| ***Brosmophyciops*** | *Lepophidium kallion* | *Narcetes lloydi* | *Dolichopteroides binocularis* |
| *Brosmophyciops pautzkei* | *Lepophidium marmoratum* | *Narcetes stomias* | ***Dolichopteryx*** |
| ***Brosmophycis*** | *Lepophidium microlepis* | ***Photostylus*** | *Dolichopteryx longipes* |
| *Brosmophycis marginata* | *Lepophidium negropinna* | *Photostylus pycnopterus* | ***Macropinna*** |
| ***Cataetyx*** | *Lepophidium pardale* | ***Rinoctes*** | *Macropinna microstoma* |
| *Cataetyx alleni* | *Lepophidium pheromystax* | *Rinoctes nasutus* | ***Monacoa*** |
| *Cataetyx laticeps* | *Lepophidium profundorum* | ***Rouleina*** | *Monacoa grimaldii* |
| *Cataetyx messieri* | *Lepophidium prorates* | *Rouleina attrita* | ***Opisthoproctus*** |
| *Cataetyx rubrirostris* | *Lepophidium robustum* | *Rouleina guentheri* | *Opisthoproctus soleatus* |
| ***Dermatopsis*** | *Lepophidium staurophor* | *Rouleina maderensis* | ***Rhynchohyalus*** |
| *Dermatopsis hoesei* | *Lepophidium wileyi* | *Rouleina squamilatera* | *Rhynchohyalus natalensis* |
| *Dermatopsis multiradiatus* | *Lepophidium zophochir* | ***Talismania*** | ***Winteria*** |
| ***Dinematichthys*** | ***Monomitopus*** | *Talismania antillarum* | *Winteria telescopa* |
| *Dinematichthys iluocoeteoides* | *Monomitopus magnus* | *Talismania bifurcata* | **Osmeridae** |
| ***Dipulus*** | *Monomitopus metriostoma* | *Talismania homoptera* | ***Allosmerus*** |
| *Dipulus caecus* | ***Ophidion*** | *Talismania longifilis* | *Allosmerus elongatus* |
| ***Lucifuga*** | *Ophidion barbatum* | *Talismania mekistonema* | ***Hypomesus*** |
| *Lucifuga spelaeotes* | *Ophidion galeoides* | ***Xenodermichthys*** | *Hypomesus pretiosus* |
| ***Ogilbia*** | *Ophidion grayi* | *Xenodermichthys copei* | ***Mallotus*** |
| *Ogilbia nudiceps* | *Ophidion holbrookii* | **Argentinidae** | *Mallotus villosus* |
| *Ogilbia sedorae* | *Ophidion iris* | ***Argentina*** | ***Osmerus*** |
| *Ogilbia ventralis* | *Ophidion lozanoi* | *Argentina aliceae* | *Osmerus eperlanus* |
| **Carapidae** | *Ophidion marginatum* | *Argentina australiae* | *Osmerus mordax* |
| ***Carapus*** | *Ophidion metoecus* | *Argentina euchus* | ***Spirinchus*** |
| *Carapus acus* | *Ophidion rochei* | *Argentina sialis* | *Spirinchus starksi* |
| *Carapus dubius* | *Ophidion selenops* | *Argentina silus* | *Spirinchus thaleichthys* |
| ***Echiodon*** | ***Otophidium*** | *Argentina sphyraena* | ***Thaleichthys*** |
| *Echiodon drummondii* | *Otophidium omostigma* | *Argentina striata* | *Thaleichthys pacificus* |
| ***Encheliophis*** | ***Parophidion*** | ***Glossanodon*** | **Platytroctidae** |
| *Encheliophis homei* | *Parophidion vassali* | *Glossanodon leioglossus* | ***Holtbyrnia*** |
| *Encheliophis vermicularis* | ***Raneya*** | *Glossanodon semifasciatus* | *Holtbyrnia anomala* |
| ***Pyramodon*** | *Raneya brasiliensis* | **Bathylagidae** | *Holtbyrnia conocephala* |
| *Pyramodon ventralis* | ***Selachophidium*** | ***Bathylagichthys*** | *Holtbyrnia cyanocephala* |
| **Ophidiidae** | *Selachophidium guentheri* | *Bathylagichthys greyae* | *Holtbyrnia laticauda* |
| ***Abyssobrotula*** | ***Spectrunculus*** | ***Bathylagoides*** | *Holtbyrnia latifrons* |
| *Abyssobrotula galatheae* | *Spectrunculus grandis* | *Bathylagoides argyrogaster* | *Holtbyrnia macrops* |
| ***Acanthonus*** | **Osmeriformes** | *Bathylagoides nigrigenys* | ***Maulisia*** |
| *Acanthonus armatus* | **Alepocephalidae** | *Bathylagoides wesethi* | *Maulisia argipalla* |
| ***Benthocometes*** | ***Alepocephalus*** | ***Bathylagus*** | *Maulisia mauli* |
| *Benthocometes robustus* | *Alepocephalus agassizii* | *Bathylagus antarcticus* | *Maulisia microlepis* |
| ***Brotula*** | *Alepocephalus australis* | *Bathylagus gracilis* | ***Normichthys*** |
| *Brotula barbata* | *Alepocephalus bairdii* | *Bathylagus pacificus* | *Normichthys operosus* |
| *Brotula clarkae* | *Alepocephalus productus* | *Bathylagus tenuis* | *Normichthys yahganorum* |
| *Brotula multibarbata* | *Alepocephalus rostratus* | ***Dolicholagus*** | ***Persparsia*** |
| *Brotula townsendi* | *Alepocephalus triangularis* | *Dolicholagus longirostris* | *Persparsia kopua* |
| ***Cherublemma*** | ***Asquamiceps*** | ***Leuroglossus*** | ***Platytroctes*** |
| *Cherublemma emmelas* | *Asquamiceps caeruleus* | *Leuroglossus schmidti* | *Platytroctes apus* |
| ***Chilara*** | ***Bajacalifornia*** | ***Lipolagus*** | *Platytroctes mirus* |
| *Chilara taylori* | *Bajacalifornia megalops* | *Lipolagus ochotensis* | ***Sagamichthys*** |
| ***Epetriodus*** | ***Bathylaco*** | ***Melanolagus*** | *Sagamichthys schnakenbecki* |
| *Epetriodus freddyi* | *Bathylaco nigricans* | *Melanolagus bericoides* | ***Searsia*** |
| ***Genypterus*** | ***Bathyprion*** | **Galaxiidae** | *Searsia koefoedi* |
| *Genypterus blacodes* | *Bathyprion danae* | ***Galaxias*** | **Plecoglossidae** |
| *Genypterus brasiliensis* | ***Bathytroctes*** | *Galaxias truttaceus* | ***Plecoglossus*** |
| *Genypterus capensis* | *Bathytroctes macrolepis* | **Leptochilichthyidae** | *Plecoglossus altivelis* |
| *Genypterus chilensis* | *Bathytroctes michaelsarsi* | ***Leptochilichthys*** | **Perciformes** |
| *Genypterus maculatus* | *Bathytroctes microlepis* | *Leptochilichthys agassizii* | **Acanthuridae** |
| *Genypterus tigerinus* | *Bathytroctes oligolepis* | *Leptochilichthys pinguis* | ***Acanthurus*** |
| ***Lamprogrammus*** | ***Conocara*** | **Microstomatidae** | *Acanthurus achilles* |
| *Lamprogrammus exutus* | *Conocara macropterum* | ***Microstoma*** | *Acanthurus albipectoralis* |
| *Lamprogrammus shcherbachevi* | *Conocara murrayi* | *Microstoma microstoma* | *Acanthurus auranticavus* |
|  | *Conocara salmoneum* | ***Nansenia*** | *Acanthurus bahianus* |

**Table S1 (continued).**

| *Acanthurus bariene* | *Parascombrops spinosus* | *Cheilodipterus parazonatus* | *Sphaeramia orbicularis* |
| --- | --- | --- | --- |
| *Acanthurus blochii* | ***Synagrops*** | *Cheilodipterus quinquelineatus* | ***Taeniamia*** |
| *Acanthurus chirurgus* | *Synagrops bellus* | *Cheilodipterus singapurensis* | *Taeniamia biguttata* |
| *Acanthurus coeruleus* | *Synagrops japonicus* | *Cheilodipterus zonatus* | *Taeniamia fucata* |
| *Acanthurus dussumieri* | **Amarsipidae** | ***Fibramia*** | *Taeniamia zosterophora* |
| *Acanthurus gahhm* | ***Amarsipus*** | *Fibramia amboinensis* | ***Verulux*** |
| *Acanthurus grammoptilus* | *Amarsipus carlsbergi* | *Fibramia lateralis* | *Verulux cypselurus* |
| *Acanthurus guttatus* | **Ambassidae** | ***Foa*** | ***Zapogon*** |
| *Acanthurus leucocheilus* | ***Ambassis*** | *Foa brachygramma* | *Zapogon evermanni* |
| *Acanthurus leucopareius* | *Ambassis interrupta* | ***Fowleria*** | ***Zoramia*** |
| *Acanthurus leucosternon* | *Ambassis jacksoniensis* | *Fowleria aurita* | *Zoramia fragilis* |
| *Acanthurus lineatus* | *Ambassis miops* | *Fowleria flammea* | *Zoramia gilberti* |
| *Acanthurus maculiceps* | *Ambassis natalensis* | *Fowleria isostigma* | *Zoramia leptacantha* |
| *Acanthurus mata* | *Ambassis vachellii* | *Fowleria marmorata* | *Zoramia perlita* |
| *Acanthurus monroviae* | **Ammodytidae** | *Fowleria punctulata* | **Ariommatidae** |
| *Acanthurus nigricans* | ***Ammodytes*** | *Fowleria variegata* | ***Ariomma*** |
| *Acanthurus nigricauda* | *Ammodytes americanus* | ***Gymnapogon*** | *Ariomma bondi* |
| *Acanthurus nigrofuscus* | *Ammodytes dubius* | *Gymnapogon philippinus* | *Ariomma indicum* |
| *Acanthurus nigroris* | *Ammodytes hexapterus* | *Gymnapogon urospilotus* | *Ariomma luridum* |
| *Acanthurus olivaceus* | *Ammodytes marinus* | ***Jaydia*** | *Ariomma melanum* |
| *Acanthurus pyroferus* | *Ammodytes personatus* | *Jaydia ellioti* | *Ariomma regulus* |
| *Acanthurus thompsoni* | *Ammodytes tobianus* | ***Nectamia*** | **Arripidae** |
| *Acanthurus triostegus* | ***Ammodytoides*** | *Nectamia annularis* | ***Arripis*** |
| *Acanthurus tristis* | *Ammodytoides pylei* | *Nectamia bandanensis* | *Arripis georgianus* |
| *Acanthurus xanthopterus* | ***Bleekeria*** | *Nectamia fusca* | *Arripis trutta* |
| ***Ctenochaetus*** | *Bleekeria mitsukurii* | *Nectamia ignitops* | *Arripis truttacea* |
| *Ctenochaetus binotatus* | ***Gymnammodytes*** | *Nectamia luxuria* | **Artedidraconidae** |
| *Ctenochaetus cyanocheilus* | *Gymnammodytes capensis* | *Nectamia similis* | ***Artedidraco*** |
| *Ctenochaetus hawaiiensis* | *Gymnammodytes cicerelus* | *Nectamia viria* | *Artedidraco lonnbergi* |
| *Ctenochaetus marginatus* | *Gymnammodytes semisquamatus* | ***Ostorhinchus*** | *Artedidraco mirus* |
| *Ctenochaetus striatus* | ***Hyperoplus*** | *Ostorhinchus angustatus* | *Artedidraco orianae* |
| *Ctenochaetus strigosus* | *Hyperoplus immaculatus* | *Ostorhinchus aureus* | *Artedidraco shackletoni* |
| *Ctenochaetus tominiensis* | *Hyperoplus lanceolatus* | *Ostorhinchus compressus* | *Artedidraco skottsbergi* |
| ***Naso*** | **Anarhichadidae** | *Ostorhinchus cyanosoma* | ***Dolloidraco*** |
| *Naso annulatus* | ***Anarhichas*** | *Ostorhinchus dispar* | *Dolloidraco longedorsalis* |
| *Naso brachycentron* | *Anarhichas denticulatus* | *Ostorhinchus diversus* | ***Histiodraco*** |
| *Naso brevirostris* | *Anarhichas lupus* | *Ostorhinchus hartzfeldii* | *Histiodraco velifer* |
| *Naso elegans* | *Anarhichas minor* | *Ostorhinchus margaritophorus* | ***Pogonophryne*** |
| *Naso hexacanthus* | *Anarhichas orientalis* | *Ostorhinchus nigrofasciatus* | *Pogonophryne barsukovi* |
| *Naso lituratus* | ***Anarrhichthys*** | *Ostorhinchus novemfasciatus* | *Pogonophryne lanceobarbata* |
| *Naso lopezi* | *Anarrhichthys ocellatus* | *Ostorhinchus rubrimacula* | *Pogonophryne marmorata* |
| *Naso maculatus* | **Aplodactylidae** | *Ostorhinchus rueppellii* | *Pogonophryne permitini* |
| *Naso mcdadei* | ***Aplodactylus*** | *Ostorhinchus sealei* | *Pogonophryne scotti* |
| *Naso unicornis* | *Aplodactylus arctidens* | *Ostorhinchus taeniophorus* | *Pogonophryne ventrimaculata* |
| *Naso vlamingii* | **Apogonidae** | ***Paroncheilus*** | **Bathydraconidae** |
| ***Paracanthurus*** | ***Apogon*** | *Paroncheilus affinis* | ***Akarotaxis*** |
| *Paracanthurus hepatus* | *Apogon binotatus* | ***Phaeoptyx*** | *Akarotaxis nudiceps* |
| ***Prionurus*** | *Apogon caudicinctus* | *Phaeoptyx pigmentaria* | ***Bathydraco*** |
| *Prionurus laticlavius* | *Apogon coccineus* | ***Pristiapogon*** | *Bathydraco antarcticus* |
| *Prionurus microlepidotus* | *Apogon doryssa* | *Pristiapogon exostigma* | *Bathydraco joannae* |
| *Prionurus punctatus* | *Apogon dovii* | *Pristiapogon fraenatus* | *Bathydraco macrolepis* |
| ***Zebrasoma*** | *Apogon imberbis* | *Pristiapogon kallopterus* | *Bathydraco marri* |
| *Zebrasoma desjardinii* | *Apogon maculatus* | *Pristiapogon taeniopterus* | ***Cygnodraco*** |
| *Zebrasoma flavescens* | *Apogon phenax* | ***Pristicon*** | *Cygnodraco mawsoni* |
| *Zebrasoma scopas* | *Apogon pseudomaculatus* | *Pristicon trimaculatus* | ***Gerlachea*** |
| *Zebrasoma velifer* | ***Apogonichthyoides*** | ***Pseudamia*** | *Gerlachea australis* |
| *Zebrasoma xanthurum* | *Apogonichthyoides brevicaudatus* | *Pseudamia amblyuroptera* | ***Gymnodraco*** |
| **Acropomatidae** | *Apogonichthyoides melas* | *Pseudamia gelatinosa* | *Gymnodraco acuticeps* |
| ***Acropoma*** | *Apogonichthyoides pseudotaeniatus* | *Pseudamia hayashii* | ***Parachaenichthys*** |
| *Acropoma japonicum* | ***Apogonichthys*** | *Pseudamia tarri* | *Parachaenichthys charcoti* |
| ***Apogonops*** | *Apogonichthys ocellatus* | *Pseudamia zonata* | *Parachaenichthys georgianus* |
| *Apogonops anomalus* | *Apogonichthys perdix* | ***Pseudamiops*** | ***Prionodraco*** |
| ***Caraibops*** | ***Astrapogon*** | *Pseudamiops gracilicauda* | *Prionodraco evansii* |
| *Caraibops trispinosus* | *Astrapogon alutus* | ***Rhabdamia*** | ***Psilodraco*** |
| ***Doederleinia*** | *Astrapogon stellatus* | *Rhabdamia gracilis* | *Psilodraco breviceps* |
| *Doederleinia berycoides* | ***Cheilodipterus*** | ***Siphamia*** | ***Racovitzia*** |
| ***Kaperangus*** | *Cheilodipterus arabicus* | *Siphamia cephalotes* | *Racovitzia glacialis* |
| *Kaperangus microlepis* | *Cheilodipterus artus* | *Siphamia fistulosa* | ***Vomeridens*** |
| ***Malakichthys*** | *Cheilodipterus isostigmus* | *Siphamia fuscolineata* | *Vomeridens infuscipinnis* |
| *Malakichthys elegans* | *Cheilodipterus lachneri* | *Siphamia randalli* | **Bathymasteridae** |
| ***Parascombrops*** | *Cheilodipterus macrodon* | ***Sphaeramia*** | ***Bathymaster*** |
| *Parascombrops philippinensis* | *Cheilodipterus novemstriatus* | *Sphaeramia nematoptera* | *Bathymaster caeruleofasciatus* |

**Table S1 (continued).**

| **Blenniidae** | ***Glyptoparus*** | *Salarias luctuosus* | ***Diplogrammus*** |
| --- | --- | --- | --- |
| ***Aidablennius*** | *Glyptoparus delicatulus* | ***Scartichthys*** | *Diplogrammus goramensis* |
| *Aidablennius sphynx* | ***Hypleurochilus*** | *Scartichthys gigas* | *Diplogrammus pauciradiatus* |
| ***Alticus*** | *Hypleurochilus bermudensis* | *Scartichthys variolatus* | ***Foetorepus*** |
| *Alticus saliens* | *Hypleurochilus geminatus* | ***Stanulus*** | *Foetorepus agassizii* |
| ***Aspidontus*** | ***Hypsoblennius*** | *Stanulus seychellensis* | *Foetorepus phasis* |
| *Aspidontus dussumieri* | *Hypsoblennius gentilis* | ***Xiphasia*** | ***Neosynchiropus*** |
| *Aspidontus taeniatus* | *Hypsoblennius gilberti* | *Xiphasia matsubarai* | *Neosynchiropus ocellatus* |
| ***Atrosalarias*** | *Hypsoblennius hentz* | **Bovichtidae** | ***Synchiropus*** |
| *Atrosalarias fuscus* | *Hypsoblennius invemar* | ***Cottoperca*** | *Synchiropus altivelis* |
| *Atrosalarias holomelas* | ***Istiblennius*** | *Cottoperca gobio* | *Synchiropus circularis* |
| ***Blenniella*** | *Istiblennius edentulus* | **Bramidae** | *Synchiropus goodenbeani* |
| *Blenniella bilitonensis* | *Istiblennius lineatus* | ***Brama*** | *Synchiropus laddi* |
| *Blenniella caudolineata* | *Istiblennius unicolor* | *Brama brama* | *Synchiropus morrisoni* |
| *Blenniella chrysospilos* | ***Lipophrys*** | *Brama dussumieri* | *Synchiropus phaeton* |
| *Blenniella periophthalmus* | *Lipophrys pholis* | *Brama japonica* | *Synchiropus picturatus* |
| ***Blennius*** | *Lipophrys trigloides* | ***Eumegistus*** | **Caproidae** |
| *Blennius ocellaris* | ***Litobranchus*** | *Eumegistus brevorti* | ***Antigonia*** |
| ***Chasmodes*** | *Litobranchus fowleri* | ***Pterycombus*** | *Antigonia capros* |
| *Chasmodes bosquianus* | ***Meiacanthus*** | *Pterycombus brama* | *Antigonia combatia* |
| *Chasmodes saburrae* | *Meiacanthus anema* | ***Taractes*** | *Antigonia rhomboidea* |
| ***Cirripectes*** | *Meiacanthus atrodorsalis* | *Taractes asper* | ***Capros*** |
| *Cirripectes alboapicalis* | *Meiacanthus ditrema* | ***Taractichthys*** | *Capros aper* |
| *Cirripectes auritus* | *Meiacanthus grammistes* | *Taractichthys longipinnis* | **Carangidae** |
| *Cirripectes castaneus* | *Meiacanthus luteus* | *Taractichthys steindachneri* | ***Alectis*** |
| *Cirripectes chelomatus* | ***Microlipophrys*** | **Caesionidae** | *Alectis alexandrina* |
| *Cirripectes filamentosus* | *Microlipophrys adriaticus* | ***Caesio*** | *Alectis ciliaris* |
| *Cirripectes fuscoguttatus* | *Microlipophrys canevae* | *Caesio caerulaurea* | *Alectis indica* |
| *Cirripectes gilberti* | *Microlipophrys nigriceps* | *Caesio cuning* | ***Alepes*** |
| *Cirripectes hutchinsi* | ***Nannosalarias*** | *Caesio lunaris* | *Alepes djedaba* |
| *Cirripectes imitator* | *Nannosalarias nativitatis* | *Caesio striata* | *Alepes kleinii* |
| *Cirripectes jenningsi* | ***Omobranchus*** | *Caesio suevica* | *Alepes melanoptera* |
| *Cirripectes obscurus* | *Omobranchus obliquus* | *Caesio teres* | ***Atule*** |
| *Cirripectes perustus* | *Omobranchus punctatus* | *Caesio varilineata* | *Atule mate* |
| *Cirripectes polyzona* | ***Omox*** | *Caesio xanthonota* | ***Campogramma*** |
| *Cirripectes quagga* | *Omox biporos* | ***Dipterygonotus*** | *Campogramma glaycos* |
| *Cirripectes randalli* | ***Ophioblennius*** | *Dipterygonotus balteatus* | ***Carangoides*** |
| *Cirripectes springeri* | *Ophioblennius steindachneri* | ***Gymnocaesio*** | *Carangoides armatus* |
| *Cirripectes stigmaticus* | ***Parablennius*** | *Gymnocaesio gymnoptera* | *Carangoides bajad* |
| *Cirripectes vanderbilti* | *Parablennius gattorugine* | ***Pterocaesio*** | *Carangoides bartholomaei* |
| *Cirripectes variolosus* | *Parablennius incognitus* | *Pterocaesio chrysozona* | *Carangoides chrysophrys* |
| ***Coryphoblennius*** | *Parablennius marmoreus* | *Pterocaesio digramma* | *Carangoides coeruleopinnatus* |
| *Coryphoblennius galerita* | *Parablennius parvicornis* | *Pterocaesio lativittata* | *Carangoides dinema* |
| ***Ecsenius*** | *Parablennius pilicornis* | *Pterocaesio marri* | *Carangoides equula* |
| *Ecsenius axelrodi* | *Parablennius rouxi* | *Pterocaesio pisang* | *Carangoides ferdau* |
| *Ecsenius bicolor* | *Parablennius sanguinolentus* | *Pterocaesio randalli* | *Carangoides fulvoguttatus* |
| *Ecsenius bimaculatus* | *Parablennius tentacularis* | *Pterocaesio tessellata* | *Carangoides gymnostethus* |
| *Ecsenius dilemma* | *Parablennius zvonimiri* | *Pterocaesio tile* | *Carangoides orthogrammus* |
| *Ecsenius fourmanoiri* | ***Paralticus*** | *Pterocaesio trilineata* | *Carangoides otrynter* |
| *Ecsenius lividanalis* | *Paralticus amboinensis* | **Callanthiidae** | *Carangoides plagiotaenia* |
| *Ecsenius melarchus* | ***Parenchelyurus*** | ***Callanthias*** | ***Caranx*** |
| *Ecsenius monoculus* | *Parenchelyurus hepburni* | *Callanthias ruber* | *Caranx bucculentus* |
| *Ecsenius namiyei* | ***Petroscirtes*** | ***Grammatonotus*** | *Caranx caballus* |
| *Ecsenius oculus* | *Petroscirtes breviceps* | *Grammatonotus laysanus* | *Caranx caninus* |
| *Ecsenius opsifrontalis* | *Petroscirtes mitratus* | **Callionymidae** | *Caranx crysos* |
| *Ecsenius pictus* | *Petroscirtes thepassii* | ***Anaora*** | *Caranx heberi* |
| *Ecsenius sellifer* | *Petroscirtes variabilis* | *Anaora tentaculata* | *Caranx hippos* |
| *Ecsenius stigmatura* | *Petroscirtes xestus* | ***Callionymus*** | *Caranx ignobilis* |
| *Ecsenius yaeyamaensis* | ***Plagiotremus*** | *Callionymus bairdi* | *Caranx latus* |
| ***Enchelyurus*** | *Plagiotremus laudandus* | *Callionymus delicatulus* | *Caranx lugubris* |
| *Enchelyurus kraussii* | *Plagiotremus rhinorhynchos* | *Callionymus enneactis* | *Caranx melampygus* |
| ***Entomacrodus*** | *Plagiotremus tapeinosoma* | *Callionymus fasciatus* | *Caranx papuensis* |
| *Entomacrodus caudofasciatus* | ***Praealticus*** | *Callionymus filamentosus* | *Caranx rhonchus* |
| *Entomacrodus chiostictus* | *Praealticus natalis* | *Callionymus hindsii* | *Caranx ruber* |
| *Entomacrodus decussatus* | ***Rhabdoblennius*** | *Callionymus lyra* | *Caranx senegallus* |
| *Entomacrodus nigricans* | *Rhabdoblennius rhabdotrachelus* | *Callionymus maculatus* | *Caranx sexfasciatus* |
| *Entomacrodus niuafoouensis* | ***Salaria*** | *Callionymus pusillus* | *Caranx tille* |
| *Entomacrodus sealei* | *Salaria basilisca* | *Callionymus reticulatus* | *Caranx vinctus* |
| *Entomacrodus striatus* | *Salaria fluviatilis* | *Callionymus risso* | ***Chloroscombrus*** |
| *Entomacrodus thalassinus* | *Salaria pavo* | *Callionymus sagitta* | *Chloroscombrus chrysurus* |
| ***Exallias*** | ***Salarias*** | *Callionymus simplicicornis* | *Chloroscombrus orqueta* |
| *Exallias brevis* | *Salarias fasciatus* |  | ***Decapterus*** |

**Table S1 (continued).**

| *Decapterus kurroides* | *Trachurus murphyi* | ***Coralliozetus*** | ***Hemitaurichthys*** |
| --- | --- | --- | --- |
| *Decapterus macarellus* | *Trachurus novaezelandiae* | *Coralliozetus angelicus* | *Hemitaurichthys polylepis* |
| *Decapterus macrosoma* | *Trachurus picturatus* | ***Emblemaria*** | *Hemitaurichthys thompsoni* |
| *Decapterus maruadsi* | *Trachurus symmetricus* | *Emblemaria atlantica* | ***Heniochus*** |
| *Decapterus punctatus* | *Trachurus trecae* | *Emblemaria pandionis* | *Heniochus acuminatus* |
| *Decapterus russelli* | ***Uraspis*** | *Emblemaria piratula* | *Heniochus chrysostomus* |
| *Decapterus tabl* | *Uraspis helvola* | ***Neoclinus*** | *Heniochus diphreutes* |
| ***Elagatis*** | *Uraspis uraspis* | *Neoclinus blanchardi* | *Heniochus monoceros* |
| *Elagatis bipinnulata* | **Caristiidae** | *Neoclinus stephensae* | *Heniochus singularius* |
| ***Hemicaranx*** | ***Caristius*** | *Neoclinus uninotatus* | *Heniochus varius* |
| *Hemicaranx amblyrhynchus* | *Caristius macropus* | ***Stathmonotus*** | ***Johnrandallia*** |
| *Hemicaranx leucurus* | ***Neocaristius*** | *Stathmonotus sinuscalifornici* | *Johnrandallia nigrirostris* |
| *Hemicaranx zelotes* | *Neocaristius heemstrai* | **Chaetodontidae** | ***Prognathodes*** |
| ***Lichia*** | ***Paracaristius*** | ***Amphichaetodon*** | *Prognathodes aculeatus* |
| *Lichia amia* | *Paracaristius aquilus* | *Amphichaetodon melbae* | *Prognathodes aya* |
| ***Megalaspis*** | *Paracaristius maderensis* | ***Chaetodon*** | *Prognathodes marcellae* |
| *Megalaspis cordyla* | *Paracaristius nemorosus* | *Chaetodon adiergastos* | **Champsodontidae** |
| ***Naucrates*** | *Paracaristius nudarcus* | *Chaetodon assarius* | ***Champsodon*** |
| *Naucrates ductor* | ***Platyberyx*** | *Chaetodon aureofasciatus* | *Champsodon guentheri* |
| ***Oligoplites*** | *Platyberyx andriashevi* | *Chaetodon auriga* | **Channichthyidae** |
| *Oligoplites altus* | *Platyberyx opalescens* | *Chaetodon auripes* | ***Chaenocephalus*** |
| *Oligoplites palometa* | **Centrolophidae** | *Chaetodon austriacus* | *Chaenocephalus aceratus* |
| *Oligoplites refulgens* | ***Centrolophus*** | *Chaetodon baronessa* | ***Chaenodraco*** |
| *Oligoplites saliens* | *Centrolophus niger* | *Chaetodon bennetti* | *Chaenodraco wilsoni* |
| *Oligoplites saurus* | ***Hyperoglyphe*** | *Chaetodon burgessi* | ***Champsocephalus*** |
| ***Parastromateus*** | *Hyperoglyphe antarctica* | *Chaetodon capistratus* | *Champsocephalus esox* |
| *Parastromateus niger* | *Hyperoglyphe bythites* | *Chaetodon citrinellus* | *Champsocephalus gunnari* |
| ***Parona*** | *Hyperoglyphe perciformis* | *Chaetodon ephippium* | ***Channichthys*** |
| *Parona signata* | ***Icichthys*** | *Chaetodon falcula* | *Channichthys rhinoceratus* |
| ***Pseudocaranx*** | *Icichthys australis* | *Chaetodon flavirostris* | ***Chionobathyscus*** |
| *Pseudocaranx dentex* | *Icichthys lockingtoni* | *Chaetodon humeralis* | *Chionobathyscus dewitti* |
| ***Scomberoides*** | ***Psenopsis*** | *Chaetodon interruptus* | ***Chionodraco*** |
| *Scomberoides lysan* | *Psenopsis anomala* | *Chaetodon kleinii* | *Chionodraco hamatus* |
| *Scomberoides tala* | ***Schedophilus*** | *Chaetodon lineolatus* | *Chionodraco myersi* |
| ***Selar*** | *Schedophilus medusophagus* | *Chaetodon marleyi* | *Chionodraco rastrospinosus* |
| *Selar boops* | *Schedophilus ovalis* | *Chaetodon melannotus* | ***Cryodraco*** |
| *Selar crumenophthalmus* | *Schedophilus velaini* | *Chaetodon mertensii* | *Cryodraco antarcticus* |
| ***Selaroides*** | ***Seriolella*** | *Chaetodon meyeri* | ***Dacodraco*** |
| *Selaroides leptolepis* | *Seriolella brama* | *Chaetodon nippon* | *Dacodraco hunteri* |
| ***Selene*** | *Seriolella caerulea* | *Chaetodon ocellatus* | ***Neopagetopsis*** |
| *Selene brevoortii* | *Seriolella porosa* | *Chaetodon ocellicaudus* | *Neopagetopsis ionah* |
| *Selene dorsalis* | *Seriolella punctata* | *Chaetodon octofasciatus* | ***Pagetopsis*** |
| *Selene orstedii* | **Centropomidae** | *Chaetodon ornatissimus* | *Pagetopsis macropterus* |
| *Selene setapinnis* | ***Centropomus*** | *Chaetodon oxycephalus* | *Pagetopsis maculatus* |
| *Selene vomer* | *Centropomus armatus* | *Chaetodon pelewensis* | ***Pseudochaenichthys*** |
| ***Seriola*** | *Centropomus ensiferus* | *Chaetodon plebeius* | *Pseudochaenichthys georgianus* |
| *Seriola dumerili* | *Centropomus medius* | *Chaetodon punctatofasciatus* | **Cheilodactylidae** |
| *Seriola fasciata* | *Centropomus mexicanus* | *Chaetodon quadrimaculatus* | ***Cheilodactylus*** |
| *Seriola hippos* | *Centropomus nigrescens* | *Chaetodon rafflesii* | *Cheilodactylus variegatus* |
| *Seriola lalandi* | *Centropomus parallelus* | *Chaetodon rainfordi* | ***Chirodactylus*** |
| *Seriola peruana* | *Centropomus pectinatus* | *Chaetodon reticulatus* | *Chirodactylus brachydactylus* |
| *Seriola rivoliana* | *Centropomus robalito* | *Chaetodon robustus* | *Chirodactylus grandis* |
| *Seriola zonata* | *Centropomus undecimalis* | *Chaetodon sedentarius* | *Chirodactylus jessicalenorum* |
| ***Seriolina*** | *Centropomus unionensis* | *Chaetodon semeion* | ***Nemadactylus*** |
| *Seriolina nigrofasciata* | *Centropomus viridis* | *Chaetodon speculum* | *Nemadactylus bergi* |
| ***Trachinotus*** | **Cepolidae** | *Chaetodon striatus* | *Nemadactylus douglasii* |
| *Trachinotus baillonii* | ***Acanthocepola*** | *Chaetodon tinkeri* | *Nemadactylus macropterus* |
| *Trachinotus blochii* | *Acanthocepola abbreviata* | *Chaetodon tricinctus* | **Chiasmodontidae** |
| *Trachinotus carolinus* | *Acanthocepola krusensternii* | *Chaetodon trifascialis* | ***Chiasmodon*** |
| *Trachinotus cayennensis* | *Acanthocepola limbata* | *Chaetodon ulietensis* | *Chiasmodon niger* |
| *Trachinotus falcatus* | ***Cepola*** | *Chaetodon unimaculatus* | *Chiasmodon subniger* |
| *Trachinotus goodei* | *Cepola macrophthalma* | *Chaetodon vagabundus* | ***Dysalotus*** |
| *Trachinotus goreensis* | **Chaenopsidae** | ***Chelmon*** | *Dysalotus alcocki* |
| *Trachinotus kennedyi* | ***Acanthemblemaria*** | *Chelmon marginalis* | *Dysalotus oligoscolus* |
| *Trachinotus ovatus* | *Acanthemblemaria aspera* | *Chelmon muelleri* | ***Kali*** |
| *Trachinotus teraia* | *Acanthemblemaria balanorum* | ***Chelmonops*** | *Kali indica* |
| ***Trachurus*** | *Acanthemblemaria crockeri* | *Chelmonops curiosus* | ***Pseudoscopelus*** |
| *Trachurus capensis* | *Acanthemblemaria exilispinus* | ***Coradion*** | *Pseudoscopelus sagamianus* |
| *Trachurus declivis* | *Acanthemblemaria hancocki* | *Coradion chrysozonus* | *Pseudoscopelus scriptus* |
| *Trachurus japonicus* | *Acanthemblemaria macrospilus* | ***Forcipiger*** | **Chironemidae** |
| *Trachurus lathami* | *Acanthemblemaria maria* | *Forcipiger flavissimus* | ***Chironemus*** |
| *Trachurus mediterraneus* | *Acanthemblemaria spinosa* | *Forcipiger longirostris* | *Chironemus marmoratus* |

**Table S1 (continued).**

| **Cichlidae** | *Drepane punctata* | **Ephippidae** | *Eucinostomus jonesii* |
| --- | --- | --- | --- |
| ***Tilapia*** | **Echeneidae** | ***Chaetodipterus*** | *Eucinostomus melanopterus* |
| *Tilapia sparrmanii* | ***Echeneis*** | *Chaetodipterus faber* | ***Eugerres*** |
| **Cirrhitidae** | *Echeneis naucrates* | *Chaetodipterus zonatus* | *Eugerres brasilianus* |
| ***Amblycirrhitus*** | *Echeneis neucratoides* | ***Parapsettus*** | *Eugerres plumieri* |
| *Amblycirrhitus bimacula* | ***Remora*** | *Parapsettus panamensis* | ***Gerres*** |
| *Amblycirrhitus unimacula* | *Remora osteochir* | ***Platax*** | *Gerres cinereus* |
| ***Cirrhitichthys*** | *Remora remora* | *Platax orbicularis* | *Gerres erythrourus* |
| *Cirrhitichthys falco* | **Eleginopsidae** | *Platax pinnatus* | *Gerres filamentosus* |
| *Cirrhitichthys oxycephalus* | ***Eleginops*** | *Platax teira* | *Gerres longirostris* |
| ***Cirrhitus*** | *Eleginops maclovinus* | **Epigonidae** | *Gerres methueni* |
| *Cirrhitus pinnulatus* | **Eleotridae** | ***Epigonus*** | *Gerres nigri* |
| *Cirrhitus rivulatus* | ***Bunaka*** | *Epigonus constanciae* | *Gerres oblongus* |
| ***Neocirrhites*** | *Bunaka gyrinoides* | *Epigonus crassicaudus* | *Gerres oyena* |
| *Neocirrhites armatus* | ***Calumia*** | *Epigonus denticulatus* | ***Parequula*** |
| ***Oxycirrhites*** | *Calumia godeffroyi* | *Epigonus lenimen* | *Parequula melbournensis* |
| *Oxycirrhites typus* | ***Dormitator*** | *Epigonus pandionis* | ***Ulaema*** |
| ***Paracirrhites*** | *Dormitator latifrons* | *Epigonus robustus* | *Ulaema lefroyi* |
| *Paracirrhites arcatus* | *Dormitator lebretonis* | *Epigonus telescopus* | **Glaucosomatidae** |
| *Paracirrhites forsteri* | *Dormitator maculatus* | **Gempylidae** | ***Glaucosoma*** |
| *Paracirrhites hemistictus* | ***Eleotris*** | ***Diplospinus*** | *Glaucosoma buergeri* |
| **Clinidae** | *Eleotris fusca* | *Diplospinus multistriatus* | *Glaucosoma hebraicum* |
| ***Blennophis*** | *Eleotris melanosoma* | ***Epinnula*** | *Glaucosoma magnificum* |
| *Blennophis anguillaris* | *Eleotris pisonis* | *Epinnula magistralis* | *Glaucosoma scapulare* |
| ***Clinitrachus*** | *Eleotris senegalensis* | ***Gempylus*** | **Gobiidae** |
| *Clinitrachus argentatus* | ***Giuris*** | *Gempylus serpens* | ***Acanthogobius*** |
| ***Clinus*** | *Giuris margaritacea* | ***Lepidocybium*** | *Acanthogobius flavimanus* |
| *Clinus agilis* | ***Gobiomorphus*** | *Lepidocybium flavobrunneum* | ***Acentrogobius*** |
| *Clinus superciliosus* | *Gobiomorphus gobioides* | ***Nealotus*** | *Acentrogobius caninus* |
| *Clinus venustris* | ***Gobiomorus*** | *Nealotus tripes* | *Acentrogobius nebulosus* |
| ***Gibbonsia*** | *Gobiomorus dormitor* | ***Neoepinnula*** | ***Amblyeleotris*** |
| *Gibbonsia elegans* | **Embiotocidae** | *Neoepinnula americana* | *Amblyeleotris fasciata* |
| *Gibbonsia metzi* | ***Amphistichus*** | *Neoepinnula orientalis* | *Amblyeleotris fontanesii* |
| *Gibbonsia montereyensis* | *Amphistichus argenteus* | ***Nesiarchus*** | *Amblyeleotris guttata* |
| ***Heterostichus*** | *Amphistichus koelzi* | *Nesiarchus nasutus* | *Amblyeleotris periophthalma* |
| *Heterostichus rostratus* | *Amphistichus rhodoterus* | ***Paradiplospinus*** | *Amblyeleotris randalli* |
| ***Muraenoclinus*** | ***Brachyistius*** | *Paradiplospinus antarcticus* | *Amblyeleotris steinitzi* |
| *Muraenoclinus dorsalis* | *Brachyistius frenatus* | *Paradiplospinus gracilis* | *Amblyeleotris wheeleri* |
| ***Pavoclinus*** | ***Cymatogaster*** | ***Promethichthys*** | ***Amblygobius*** |
| *Pavoclinus pavo* | *Cymatogaster aggregata* | *Promethichthys prometheus* | *Amblygobius decussatus* |
| ***Springeratus*** | ***Embiotoca*** | ***Rexea*** | *Amblygobius nocturnus* |
| *Springeratus xanthosoma* | *Embiotoca jacksoni* | *Rexea alisae* | ***Aphia*** |
| **Coryphaenidae** | *Embiotoca lateralis* | *Rexea antefurcata* | *Aphia minuta* |
| ***Coryphaena*** | ***Hyperprosopon*** | *Rexea bengalensis* | ***Asterropteryx*** |
| *Coryphaena equiselis* | *Hyperprosopon anale* | *Rexea brevilineata* | *Asterropteryx ensifera* |
| *Coryphaena hippurus* | *Hyperprosopon argenteum* | *Rexea nakamurai* | *Asterropteryx semipunctata* |
| **Creediidae** | *Hyperprosopon ellipticum* | *Rexea prometheoides* | ***Bathygobius*** |
| ***Chalixodytes*** | ***Hypsurus*** | *Rexea solandri* | *Bathygobius coalitus* |
| *Chalixodytes tauensis* | *Hypsurus caryi* | ***Rexichthys*** | *Bathygobius cocosensis* |
| **Cryptacanthodidae** | ***Micrometrus*** | *Rexichthys johnpaxtoni* | *Bathygobius cotticeps* |
| ***Cryptacanthodes*** | *Micrometrus aurora* | ***Ruvettus*** | *Bathygobius curacao* |
| *Cryptacanthodes giganteus* | *Micrometrus minimus* | *Ruvettus pretiosus* | *Bathygobius fuscus* |
| *Cryptacanthodes maculatus* | ***Phanerodon*** | ***Thyrsites*** | *Bathygobius soporator* |
| **Dactyloscopidae** | *Phanerodon atripes* | *Thyrsites atun* | ***Bollmannia*** |
| ***Dactyloscopus*** | *Phanerodon furcatus* | ***Thyrsitoides*** | *Bollmannia chlamydes* |
| *Dactyloscopus crossotus* | ***Rhacochilus*** | *Thyrsitoides marleyi* | ***Bryaninops*** |
| *Dactyloscopus foraminosus* | *Rhacochilus toxotes* | ***Thyrsitops*** | *Bryaninops amplus* |
| *Dactyloscopus moorei* | *Rhacochilus vacca* | *Thyrsitops lepidopoides* | *Bryaninops erythrops* |
| *Dactyloscopus tridigitatus* | ***Zalembius*** | ***Tongaichthys*** | *Bryaninops natans* |
| ***Gillellus*** | *Zalembius rosaceus* | *Tongaichthys robustus* | *Bryaninops ridens* |
| *Gillellus healae* | **Emmelichthyidae** | **Gerreidae** | *Bryaninops yongei* |
| **Dichistiidae** | ***Emmelichthys*** | ***Diapterus*** | ***Buenia*** |
| ***Dichistius*** | *Emmelichthys nitidus nitidus* | *Diapterus auratus* | *Buenia affinis* |
| *Dichistius capensis* | *Emmelichthys ruber* | *Diapterus peruvianus* | *Buenia jeffreysii* |
| **Dinopercidae** | ***Erythrocles*** | *Diapterus rhombeus* | ***Cabillus*** |
| ***Centrarchops*** | *Erythrocles monodi* | ***Eucinostomus*** | *Cabillus tongarevae* |
| *Centrarchops chapini* | *Erythrocles scintillans* | *Eucinostomus argenteus* | ***Callogobius*** |
| ***Dinoperca*** | ***Plagiogeneion*** | *Eucinostomus currani* | *Callogobius centrolepis* |
| *Dinoperca petersi* | *Plagiogeneion rubiginosum* | *Eucinostomus entomelas* | *Callogobius hasseltii* |
| **Drepaneidae** | **Enoplosidae** | *Eucinostomus gracilis* | *Callogobius maculipinnis* |
| ***Drepane*** | ***Enoplosus*** | *Eucinostomus gula* | *Callogobius mucosus* |
| *Drepane africana* | *Enoplosus armatus* | *Eucinostomus havana* | *Callogobius okinawae* |

**Table S1 (continued).**

| *Callogobius sclateri* | *Glossogobius celebius* | ***Millerigobius*** | ***Tigrigobius*** |
| --- | --- | --- | --- |
| ***Chromogobius*** | *Glossogobius giuris* | *Millerigobius macrocephalus* | *Tigrigobius multifasciatus* |
| *Chromogobius quadrivittatus* | ***Gnatholepis*** | ***Mugilogobius*** | ***Trimma*** |
| ***Clevelandia*** | *Gnatholepis cauerensis* | *Mugilogobius littoralis* | *Trimma naudei* |
| *Clevelandia ios* | *Gnatholepis thompsoni* | ***Neogobius*** | *Trimma okinawae* |
| ***Coryphopterus*** | ***Gobiodon*** | *Neogobius melanostomus* | *Trimma taylori* |
| *Coryphopterus glaucofraenum* | *Gobiodon albofasciatus* | ***Nes*** | *Trimma tevegae* |
| *Coryphopterus hyalinus* | *Gobiodon citrinus* | *Nes longus* | *Trimma trioculatum* |
| *Coryphopterus lipernes* | *Gobiodon okinawae* | ***Nesogobius*** | ***Trimmatom*** |
| *Coryphopterus personatus* | *Gobiodon quinquestrigatus* | *Nesogobius hinsbyi* | *Trimmatom eviotops* |
| *Coryphopterus urospilus* | *Gobiodon rivulatus* | ***Odondebuenia*** | *Trimmatom nanus* |
| ***Croilia*** | ***Gobionellus*** | *Odondebuenia balearica* | ***Valenciennea*** |
| *Croilia mossambica* | *Gobionellus occidentalis* | ***Oplopomops*** | *Valenciennea helsdingenii* |
| ***Cryptocentroides*** | *Gobionellus oceanicus* | *Oplopomops diacanthus* | *Valenciennea muralis* |
| *Cryptocentroides insignis* | ***Gobiopsis*** | ***Opua*** | *Valenciennea puellaris* |
| ***Cryptocentrus*** | *Gobiopsis bravoi* | *Opua nephodes* | *Valenciennea sexguttata* |
| *Cryptocentrus cinctus* | ***Gobiosoma*** | ***Oxyurichthys*** | *Valenciennea strigata* |
| *Cryptocentrus strigilliceps* | *Gobiosoma bosc* | *Oxyurichthys stigmalophius* | ***Vanderhorstia*** |
| ***Crystallogobius*** | *Gobiosoma ginsburgi* | ***Paragobiodon*** | *Vanderhorstia ambanoro* |
| *Crystallogobius linearis* | ***Gobius*** | *Paragobiodon echinocephalus* | *Vanderhorstia ornatissima* |
| ***Ctenogobiops*** | *Gobius auratus* | *Paragobiodon lacunicolus* | ***Zebrus*** |
| *Ctenogobiops aurocingulus* | *Gobius bucchichi* | *Paragobiodon melanosomus* | *Zebrus zebrus* |
| *Ctenogobiops feroculus* | *Gobius cobitis* | *Paragobiodon modestus* | ***Zosterisessor*** |
| *Ctenogobiops pomastictus* | *Gobius couchi* | *Paragobiodon xanthosoma* | *Zosterisessor ophiocephalus* |
| *Ctenogobiops tangaroai* | *Gobius cruentatus* | ***Periophthalmus*** | **Grammatidae** |
| ***Ctenogobius*** | *Gobius gasteveni* | *Periophthalmus barbarus* | ***Gramma*** |
| *Ctenogobius boleosoma* | *Gobius geniporus* | *Periophthalmus kalolo* | *Gramma brasiliensis* |
| *Ctenogobius sagittula* | *Gobius niger* | ***Pleurosicya*** | **Haemulidae** |
| ***Deltentosteus*** | *Gobius paganellus* | *Pleurosicya bilobata* | ***Anisotremus*** |
| *Deltentosteus quadrimaculatus* | *Gobius vittatus* | *Pleurosicya micheli* | *Anisotremus caesius* |
| ***Elacatinus*** | *Gobius xanthocephalus* | *Pleurosicya muscarum* | *Anisotremus davidsonii* |
| *Elacatinus figaro* | ***Gobiusculus*** | ***Pomatoschistus*** | *Anisotremus interruptus* |
| *Elacatinus oceanops* | *Gobiusculus flavescens* | *Pomatoschistus bathi* | *Anisotremus moricandi* |
| *Elacatinus xanthiprora* | ***Ilypnus*** | *Pomatoschistus lozanoi* | *Anisotremus scapularis* |
| ***Evermannichthys*** | *Ilypnus gilberti* | *Pomatoschistus marmoratus* | *Anisotremus surinamensis* |
| *Evermannichthys metzelaari* | ***Istigobius*** | *Pomatoschistus microps* | *Anisotremus taeniatus* |
| ***Eviota*** | *Istigobius campbelli* | *Pomatoschistus minutus* | *Anisotremus virginicus* |
| *Eviota afelei* | *Istigobius decoratus* | *Pomatoschistus norvegicus* | ***Brachydeuterus*** |
| *Eviota albolineata* | *Istigobius hoesei* | *Pomatoschistus pictus* | *Brachydeuterus auritus* |
| *Eviota atriventris* | *Istigobius hoshinonis* | *Pomatoschistus quagga* | ***Conodon*** |
| *Eviota bifasciata* | *Istigobius nigroocellatus* | ***Priolepis*** | *Conodon nobilis* |
| *Eviota distigma* | *Istigobius ornatus* | *Priolepis cincta* | *Conodon serrifer* |
| *Eviota fasciola* | *Istigobius rigilius* | *Priolepis inhaca* | ***Diagramma*** |
| *Eviota herrei* | *Istigobius spence* | *Priolepis semidoliata* | *Diagramma pictum* |
| *Eviota hinanoae* | ***Kelloggella*** | ***Proterorhinus*** | ***Genyatremus*** |
| *Eviota infulata* | *Kelloggella cardinalis* | *Proterorhinus marmoratus* | *Genyatremus dovii* |
| *Eviota lachdeberei* | ***Knipowitschia*** | ***Pseudaphya*** | *Genyatremus luteus* |
| *Eviota latifasciata* | *Knipowitschia caucasica* | *Pseudaphya ferreri* | *Genyatremus pacifici* |
| *Eviota melasma* | ***Koumansetta*** | ***Redigobius*** | ***Haemulon*** |
| *Eviota pellucida* | *Koumansetta hectori* | *Redigobius bikolanus* | *Haemulon album* |
| *Eviota prasina* | *Koumansetta rainfordi* | *Redigobius tambujon* | *Haemulon aurolineatum* |
| *Eviota queenslandica* | ***Lebetus*** | ***Rhinogobiops*** | *Haemulon boschmae* |
| *Eviota saipanensis* | *Lebetus guilleti* | *Rhinogobiops nicholsii* | *Haemulon californiensis* |
| *Eviota sebreei* | *Lebetus scorpioides* | ***Rhinogobius*** | *Haemulon carbonarium* |
| *Eviota smaragdus* | ***Lepidogobius*** | *Rhinogobius brunneus* | *Haemulon chrysargyreum* |
| *Eviota sparsa* | *Lepidogobius lepidus* | *Rhinogobius giurinus* | *Haemulon flaviguttatum* |
| *Eviota zonura* | ***Lesueurigobius*** | ***Sicyopterus*** | *Haemulon flavolineatum* |
| ***Evorthodus*** | *Lesueurigobius friesii* | *Sicyopterus lagocephalus* | *Haemulon macrostomum* |
| *Evorthodus lyricus* | *Lesueurigobius sanzi* | ***Signigobius*** | *Haemulon maculicauda* |
| ***Exyrias*** | *Lesueurigobius suerii* | *Signigobius biocellatus* | *Haemulon melanurum* |
| *Exyrias belissimus* | ***Lophogobius*** | ***Silhouettea*** | *Haemulon parra* |
| *Exyrias puntang* | *Lophogobius cyprinoides* | *Silhouettea aegyptia* | *Haemulon plumierii* |
| ***Fusigobius*** | ***Lotilia*** | ***Stonogobiops*** | *Haemulon sciurus* |
| *Fusigobius neophytus* | *Lotilia graciliosa* | *Stonogobiops dracula* | *Haemulon scudderii* |
| ***Gillichthys*** | ***Macrodontogobius*** | *Stonogobiops medon* | *Haemulon sexfasciatum* |
| *Gillichthys mirabilis* | *Macrodontogobius wilburi* | *Stonogobiops nematodes* | *Haemulon squamipinna* |
| ***Ginsburgellus*** | ***Mahidolia*** | *Stonogobiops xanthorhinica* | *Haemulon steindachneri* |
| *Ginsburgellus novemlineatus* | *Mahidolia mystacina* | ***Sufflogobius*** | *Haemulon striatum* |
| ***Gladiogobius*** | ***Microgobius*** | *Sufflogobius bibarbatus* | *Haemulon vittatum* |
| *Gladiogobius ensifer* | *Microgobius carri* | ***Thorogobius*** | ***Haemulopsis*** |
| ***Glossogobius*** | *Microgobius gulosus* | *Thorogobius angolensis* | *Haemulopsis axillaris* |
| *Glossogobius bicirrhosus* | *Microgobius thalassinus* | *Thorogobius ephippiatus* | *Haemulopsis leuciscus* |

**Table S1 (continued).**

| *Haemulopsis nitidus* | *Kuhlia sandvicensis* | *Coris variegata* | ***Labrus*** |
| --- | --- | --- | --- |
| ***Isacia*** | **Kyphosidae** | *Coris venusta* | *Labrus bergylta* |
| *Isacia conceptionis* | ***Girella*** | ***Ctenolabrus*** | *Labrus merula* |
| ***Orthopristis*** | *Girella elevata* | *Ctenolabrus rupestris* | *Labrus mixtus* |
| *Orthopristis cantharinus* | *Girella nigricans* | ***Cymolutes*** | *Labrus viridis* |
| *Orthopristis chalceus* | *Girella punctata* | *Cymolutes praetextatus* | ***Lachnolaimus*** |
| *Orthopristis chrysoptera* | *Girella simplicidens* | *Cymolutes torquatus* | *Lachnolaimus maximus* |
| *Orthopristis reddingi* | *Girella tricuspidata* | ***Decodon*** | ***Macropharyngodon*** |
| ***Parapristipoma*** | *Girella zonata* | *Decodon melasma* | *Macropharyngodon meleagris* |
| *Parapristipoma octolineatum* | ***Kyphosus*** | *Decodon puellaris* | *Macropharyngodon negrosensis* |
| *Parapristipoma trilineatum* | *Kyphosus analogus* | ***Diproctacanthus*** | ***Novaculichthys*** |
| ***Plectorhinchus*** | *Kyphosus azureus* | *Diproctacanthus xanthurus* | *Novaculichthys taeniourus* |
| *Plectorhinchus albovittatus* | *Kyphosus bigibbus* | ***Doratonotus*** | ***Novaculoides*** |
| *Plectorhinchus chaetodonoides* | *Kyphosus cinerascens* | *Doratonotus megalepis* | *Novaculoides macrolepidotus* |
| *Plectorhinchus diagrammus* | *Kyphosus elegans* | ***Epibulus*** | ***Oxycheilinus*** |
| *Plectorhinchus gaterinus* | *Kyphosus incisor* | *Epibulus insidiator* | *Oxycheilinus arenatus* |
| *Plectorhinchus gibbosus* | *Kyphosus ocyurus* | ***Gomphosus*** | *Oxycheilinus bimaculatus* |
| *Plectorhinchus macrolepis* | *Kyphosus sectatrix* | *Gomphosus varius* | *Oxycheilinus digramma* |
| *Plectorhinchus mediterraneus* | *Kyphosus vaigiensis* | ***Halichoeres*** | *Oxycheilinus orientalis* |
| *Plectorhinchus obscurus* | ***Medialuna*** | *Halichoeres aestuaricola* | *Oxycheilinus unifasciatus* |
| *Plectorhinchus picus* | *Medialuna californiensis* | *Halichoeres bathyphilus* | ***Oxyjulis*** |
| *Plectorhinchus polytaenia* | ***Neoscorpis*** | *Halichoeres biocellatus* | *Oxyjulis californica* |
| *Plectorhinchus schotaf* | *Neoscorpis lithophilus* | *Halichoeres bivittatus* | ***Paracheilinus*** |
| *Plectorhinchus sordidus* | **Labridae** | *Halichoeres caudalis* | *Paracheilinus carpenteri* |
| *Plectorhinchus vittatus* | ***Acantholabrus*** | *Halichoeres chierchiae* | *Paracheilinus lineopunctatus* |
| ***Pomadasys*** | *Acantholabrus palloni* | *Halichoeres chloropterus* | *Paracheilinus octotaenia* |
| *Pomadasys argenteus* | ***Anampses*** | *Halichoeres chrysus* | ***Polylepion*** |
| *Pomadasys branickii* | *Anampses caeruleopunctatus* | *Halichoeres claudia* | *Polylepion cruentum* |
| *Pomadasys crocro* | *Anampses geographicus* | *Halichoeres cyanocephalus* | ***Pseudocheilinops*** |
| *Pomadasys incisus* | *Anampses lennardi* | *Halichoeres dispilus* | *Pseudocheilinops ataenia* |
| *Pomadasys jubelini* | *Anampses melanurus* | *Halichoeres garnoti* | ***Pseudocheilinus*** |
| *Pomadasys kaakan* | *Anampses meleagrides* | *Halichoeres hortulanus* | *Pseudocheilinus evanidus* |
| *Pomadasys macracanthus* | *Anampses twistii* | *Halichoeres leucoxanthus* | *Pseudocheilinus hexataenia* |
| *Pomadasys olivaceus* | ***Bodianus*** | *Halichoeres leucurus* | *Pseudocheilinus octotaenia* |
| *Pomadasys panamensis* | *Bodianus anthioides* | *Halichoeres maculipinna* | *Pseudocheilinus tetrataenia* |
| *Pomadasys stridens* | *Bodianus axillaris* | *Halichoeres margaritaceus* | ***Pseudocoris*** |
| ***Xenichthys*** | *Bodianus bilunulatus* | *Halichoeres marginatus* | *Pseudocoris yamashiroi* |
| *Xenichthys xanti* | *Bodianus bimaculatus* | *Halichoeres melanochir* | ***Pseudodax*** |
| **Harpagiferidae** | *Bodianus diana* | *Halichoeres melanotis* | *Pseudodax moluccanus* |
| ***Harpagifer*** | *Bodianus diplotaenia* | *Halichoeres melanurus* | ***Pseudojuloides*** |
| *Harpagifer bispinis* | *Bodianus loxozonus* | *Halichoeres melasmapomus* | *Pseudojuloides atavai* |
| *Harpagifer kerguelensis* | *Bodianus mesothorax* | *Halichoeres nebulosus* | *Pseudojuloides cerasinus* |
| *Harpagifer spinosus* | *Bodianus opercularis* | *Halichoeres nicholsi* | ***Pseudolabrus*** |
| **Howellidae** | *Bodianus pulchellus* | *Halichoeres notospilus* | *Pseudolabrus luculentus* |
| ***Howella*** | *Bodianus rufus* | *Halichoeres pictus* | ***Pteragogus*** |
| *Howella atlantica* | ***Centrolabrus*** | *Halichoeres poeyi* | *Pteragogus cryptus* |
| **Icosteidae** | *Centrolabrus exoletus* | *Halichoeres prosopeion* | *Pteragogus enneacanthus* |
| ***Icosteus*** | *Centrolabrus melanocercus* | *Halichoeres radiatus* | *Pteragogus guttatus* |
| *Icosteus aenigmaticus* | ***Cheilinus*** | *Halichoeres richmondi* | ***Semicossyphus*** |
| **Istiophoridae** | *Cheilinus chlorourus* | *Halichoeres scapularis* | *Semicossyphus pulcher* |
| ***Istiompax*** | *Cheilinus fasciatus* | *Halichoeres semicinctus* | ***Stethojulis*** |
| *Istiompax indica* | *Cheilinus oxycephalus* | *Halichoeres trimaculatus* | *Stethojulis bandanensis* |
| ***Istiophorus*** | *Cheilinus trilobatus* | ***Hemigymnus*** | *Stethojulis interrupta* |
| *Istiophorus albicans* | *Cheilinus undulatus* | *Hemigymnus fasciatus* | *Stethojulis strigiventer* |
| *Istiophorus platypterus* | ***Cheilio*** | *Hemigymnus melapterus* | *Stethojulis trilineata* |
| ***Kajikia*** | *Cheilio inermis* | ***Hologymnosus*** | ***Suezichthys*** |
| *Kajikia albida* | ***Choerodon*** | *Hologymnosus annulatus* | *Suezichthys arquatus* |
| *Kajikia audax* | *Choerodon anchorago* | *Hologymnosus doliatus* | *Suezichthys aylingi* |
| ***Makaira*** | *Choerodon oligacanthus* | ***Iniistius*** | *Suezichthys caudavittatus* |
| *Makaira mazara* | ***Cirrhilabrus*** | *Iniistius aneitensis* | *Suezichthys gracilis* |
| *Makaira nigricans* | *Cirrhilabrus cyanopleura* | *Iniistius pavo* | *Suezichthys notatus* |
| ***Tetrapturus*** | *Cirrhilabrus exquisitus* | ***Labrichthys*** | *Suezichthys soelae* |
| *Tetrapturus angustirostris* | *Cirrhilabrus luteovittatus* | *Labrichthys unilineatus* | ***Symphodus*** |
| *Tetrapturus belone* | ***Clepticus*** | ***Labroides*** | *Symphodus cinereus* |
| *Tetrapturus georgii* | *Clepticus parrae* | *Labroides bicolor* | *Symphodus doderleini* |
| *Tetrapturus pfluegeri* | ***Coris*** | *Labroides dimidiatus* | *Symphodus mediterraneus* |
| **Kraemeriidae** | *Coris aygula* | *Labroides pectoralis* | *Symphodus melops* |
| ***Kraemeria*** | *Coris batuensis* | ***Labropsis*** | *Symphodus ocellatus* |
| *Kraemeria samoensis* | *Coris flavovittata* | *Labropsis alleni* | *Symphodus roissali* |
| **Kuhliidae** | *Coris gaimard* | *Labropsis australis* | *Symphodus rostratus* |
| ***Kuhlia*** | *Coris julis* | *Labropsis micronesica* | *Symphodus tinca* |
| *Kuhlia mugil* | *Coris picta* | *Labropsis xanthonota* | *Symphodus trutta* |

**Table S1 (continued).**

| ***Tautoga*** | **Lethrinidae** | *Lutjanus bengalensis* | *Pristipomoides flavipinnis* |
| --- | --- | --- | --- |
| *Tautoga onitis* | ***Gnathodentex*** | *Lutjanus biguttatus* | *Pristipomoides freemani* |
| ***Tautogolabrus*** | *Gnathodentex aureolineatus* | *Lutjanus bitaeniatus* | *Pristipomoides macrophthalmus* |
| *Tautogolabrus adspersus* | ***Gymnocranius*** | *Lutjanus bohar* | *Pristipomoides multidens* |
| ***Thalassoma*** | *Gymnocranius audleyi* | *Lutjanus boutton* | *Pristipomoides sieboldii* |
| *Thalassoma amblycephalum* | *Gymnocranius elongatus* | *Lutjanus buccanella* | *Pristipomoides typus* |
| *Thalassoma ascensionis* | *Gymnocranius euanus* | *Lutjanus campechanus* | *Pristipomoides zonatus* |
| *Thalassoma bifasciatum* | *Gymnocranius frenatus* | *Lutjanus carponotatus* | ***Randallichthys*** |
| *Thalassoma cupido* | *Gymnocranius grandoculis* | *Lutjanus coeruleolineatus* | *Randallichthys filamentosus* |
| *Thalassoma duperrey* | *Gymnocranius griseus* | *Lutjanus colorado* | ***Rhomboplites*** |
| *Thalassoma grammaticum* | *Gymnocranius microdon* | *Lutjanus cyanopterus* | *Rhomboplites aurorubens* |
| *Thalassoma hardwicke* | ***Lethrinus*** | *Lutjanus decussatus* | ***Symphorichthys*** |
| *Thalassoma hebraicum* | *Lethrinus amboinensis* | *Lutjanus dentatus* | *Symphorichthys spilurus* |
| *Thalassoma jansenii* | *Lethrinus atkinsoni* | *Lutjanus dodecacanthoides* | ***Symphorus*** |
| *Thalassoma lucasanum* | *Lethrinus atlanticus* | *Lutjanus ehrenbergii* | *Symphorus nematophorus* |
| *Thalassoma lunare* | *Lethrinus borbonicus* | *Lutjanus erythropterus* | **Luvaridae** |
| *Thalassoma lutescens* | *Lethrinus conchyliatus* | *Lutjanus fulgens* | ***Luvarus*** |
| *Thalassoma nigrofasciatum* | *Lethrinus crocineus* | *Lutjanus fulviflamma* | *Luvarus imperialis* |
| *Thalassoma pavo* | *Lethrinus enigmaticus* | *Lutjanus fulvus* | **Malacanthidae** |
| *Thalassoma purpureum* | *Lethrinus erythracanthus* | *Lutjanus gibbus* | ***Branchiostegus*** |
| *Thalassoma quinquevittatum* | *Lethrinus erythropterus* | *Lutjanus goreensis* | *Branchiostegus japonicus* |
| *Thalassoma trilobatum* | *Lethrinus genivittatus* | *Lutjanus griseus* | *Branchiostegus sawakinensis* |
| ***Wetmorella*** | *Lethrinus haematopterus* | *Lutjanus guilcheri* | *Branchiostegus semifasciatus* |
| *Wetmorella albofasciata* | *Lethrinus harak* | *Lutjanus guttatus* | ***Caulolatilus*** |
| *Wetmorella nigropinnata* | *Lethrinus laticaudis* | *Lutjanus inermis* | *Caulolatilus chrysops* |
| ***Xyrichtys*** | *Lethrinus lentjan* | *Lutjanus jocu* | *Caulolatilus cyanops* |
| *Xyrichtys splendens* | *Lethrinus mahsena* | *Lutjanus johnii* | *Caulolatilus guppyi* |
| **Labrisomidae** | *Lethrinus microdon* | *Lutjanus jordani* | *Caulolatilus hubbsi* |
| ***Gobioclinus*** | *Lethrinus miniatus* | *Lutjanus kasmira* | *Caulolatilus intermedius* |
| *Gobioclinus bucciferus* | *Lethrinus nebulosus* | *Lutjanus lemniscatus* | *Caulolatilus microps* |
| ***Labrisomus*** | *Lethrinus obsoletus* | *Lutjanus lunulatus* | ***Hoplolatilus*** |
| *Labrisomus nuchipinnis* | *Lethrinus olivaceus* | *Lutjanus lutjanus* | *Hoplolatilus cuniculus* |
| *Labrisomus philippii* | *Lethrinus ornatus* | *Lutjanus madras* | *Hoplolatilus fronticinctus* |
| ***Malacoctenus*** | *Lethrinus ravus* | *Lutjanus mahogoni* | *Hoplolatilus starcki* |
| *Malacoctenus boehlkei* | *Lethrinus reticulatus* | *Lutjanus malabaricus* | ***Lopholatilus*** |
| *Malacoctenus macropus* | *Lethrinus rubrioperculatus* | *Lutjanus mizenkoi* | *Lopholatilus chamaeleonticeps* |
| ***Paraclinus*** | *Lethrinus semicinctus* | *Lutjanus monostigma* | *Lopholatilus villarii* |
| *Paraclinus fasciatus* | *Lethrinus variegatus* | *Lutjanus notatus* | ***Malacanthus*** |
| *Paraclinus nigripinnis* | *Lethrinus xanthochilus* | *Lutjanus novemfasciatus* | *Malacanthus brevirostris* |
| ***Starksia*** | ***Monotaxis*** | *Lutjanus peru* | *Malacanthus latovittatus* |
| *Starksia ocellata* | *Monotaxis grandoculis* | *Lutjanus purpureus* | *Malacanthus plumieri* |
| **Lactariidae** | ***Wattsia*** | *Lutjanus quinquelineatus* | **Menidae** |
| ***Lactarius*** | *Wattsia mossambica* | *Lutjanus rivulatus* | ***Mene*** |
| *Lactarius lactarius* | **Lobotidae** | *Lutjanus rufolineatus* | *Mene maculata* |
| **Lateolabracidae** | ***Lobotes*** | *Lutjanus russellii* | **Microdesmidae** |
| ***Lateolabrax*** | *Lobotes surinamensis* | *Lutjanus sanguineus* | ***Gunnellichthys*** |
| *Lateolabrax japonicus* | **Lutjanidae** | *Lutjanus sebae* | *Gunnellichthys monostigma* |
| **Latidae** | ***Aphareus*** | *Lutjanus semicinctus* | *Gunnellichthys pleurotaenia* |
| ***Lates*** | *Aphareus furca* | *Lutjanus synagris* | *Gunnellichthys viridescens* |
| *Lates calcarifer* | *Aphareus rutilans* | *Lutjanus timoriensis* | ***Microdesmus*** |
| **Latridae** | ***Aprion*** | *Lutjanus viridis* | *Microdesmus longipinnis* |
| ***Latridopsis*** | *Aprion virescens* | *Lutjanus vitta* | ***Nemateleotris*** |
| *Latridopsis ciliaris* | ***Apsilus*** | *Lutjanus vivanus* | *Nemateleotris decora* |
| **Leiognathidae** | *Apsilus dentatus* | ***Macolor*** | *Nemateleotris helfrichi* |
| ***Aurigequula*** | *Apsilus fuscus* | *Macolor macularis* | *Nemateleotris magnifica* |
| *Aurigequula fasciata* | ***Etelis*** | *Macolor niger* | ***Parioglossus*** |
| ***Equulites*** | *Etelis carbunculus* | ***Ocyurus*** | *Parioglossus formosus* |
| *Equulites elongatus* | *Etelis coruscans* | *Ocyurus chrysurus* | *Parioglossus nudus* |
| *Equulites leuciscus* | *Etelis oculatus* | ***Paracaesio*** | *Parioglossus palustris* |
| *Equulites lineolatus* | *Etelis radiosus* | *Paracaesio gonzalesi* | *Parioglossus raoi* |
| *Equulites stercorarius* | ***Hoplopagrus*** | *Paracaesio kusakarii* | ***Ptereleotris*** |
| ***Eubleekeria*** | *Hoplopagrus guentherii* | *Paracaesio sordida* | *Ptereleotris arabica* |
| *Eubleekeria splendens* | ***Lipocheilus*** | *Paracaesio stonei* | *Ptereleotris evides* |
| ***Gazza*** | *Lipocheilus carnolabrum* | *Paracaesio xanthura* | *Ptereleotris grammica* |
| *Gazza achlamys* | ***Lutjanus*** | ***Pinjalo*** | *Ptereleotris hanae* |
| *Gazza minuta* | *Lutjanus adetii* | *Pinjalo lewisi* | *Ptereleotris heteroptera* |
| ***Leiognathus*** | *Lutjanus agennes* | *Pinjalo pinjalo* | *Ptereleotris microlepis* |
| *Leiognathus equulus* | *Lutjanus analis* | ***Pristipomoides*** | *Ptereleotris monoptera* |
| ***Nuchequula*** | *Lutjanus apodus* | *Pristipomoides aquilonaris* | *Ptereleotris uroditaenia* |
| *Nuchequula longicornis* | *Lutjanus aratus* | *Pristipomoides argyrogrammicus* | *Ptereleotris zebra* |
| ***Photopectoralis*** | *Lutjanus argentimaculatus* | *Pristipomoides auricilla* | **Monodactylidae** |
| *Photopectoralis bindus* | *Lutjanus argentiventris* | *Pristipomoides filamentosus* | ***Monodactylus*** |

**Table S1 (continued).**

| *Monodactylus argenteus* | ***Parascolopsis*** | ***Paranotothenia*** | *Percophis brasiliensis* |
| --- | --- | --- | --- |
| *Monodactylus sebae* | *Parascolopsis aspinosa* | *Paranotothenia magellanica* | **Pholidae** |
| **Moronidae** | *Parascolopsis boesemani* | ***Patagonotothen*** | ***Apodichthys*** |
| ***Dicentrarchus*** | *Parascolopsis eriomma* | *Patagonotothen brevicauda* | *Apodichthys flavidus* |
| *Dicentrarchus labrax* | *Parascolopsis inermis* | *Patagonotothen guntheri* | ***Pholis*** |
| *Dicentrarchus punctatus* | *Parascolopsis rufomaculatus* | *Patagonotothen ramsayi* | *Pholis fasciata* |
| ***Morone*** | *Parascolopsis tanyactis* | *Patagonotothen wiltoni* | *Pholis gunnellus* |
| *Morone americana* | *Parascolopsis tosensis* | ***Pleuragramma*** | *Pholis ornata* |
| *Morone saxatilis* | *Parascolopsis townsendi* | *Pleuragramma antarctica* | *Pholis schultzi* |
| **Mullidae** | ***Pentapodus*** | ***Trematomus*** | **Pholidichthyidae** |
| ***Mulloidichthys*** | *Pentapodus bifasciatus* | *Trematomus bernacchii* | ***Pholidichthys*** |
| *Mulloidichthys flavolineatus* | *Pentapodus caninus* | *Trematomus eulepidotus* | *Pholidichthys leucotaenia* |
| *Mulloidichthys martinicus* | *Pentapodus emeryii* | *Trematomus hansoni* | **Pinguipedidae** |
| *Mulloidichthys pfluegeri* | *Pentapodus nagasakiensis* | *Trematomus lepidorhinus* | ***Parapercis*** |
| *Mulloidichthys vanicolensis* | *Pentapodus paradiseus* | *Trematomus loennbergii* | *Parapercis clathrata* |
| ***Mullus*** | *Pentapodus porosus* | *Trematomus newnesi* | *Parapercis colias* |
| *Mullus argentinae* | *Pentapodus setosus* | *Trematomus nicolai* | *Parapercis cylindrica* |
| *Mullus auratus* | *Pentapodus trivittatus* | *Trematomus pennellii* | *Parapercis dockinsi* |
| *Mullus barbatus barbatus* | ***Scaevius*** | *Trematomus scotti* | *Parapercis hexophtalma* |
| *Mullus surmuletus* | *Scaevius milii* | *Trematomus tokarevi* | *Parapercis lineopunctata* |
| ***Parupeneus*** | ***Scolopsis*** | **Odacidae** | *Parapercis millepunctata* |
| *Parupeneus barberinoides* | *Scolopsis affinis* | ***Haletta*** | *Parapercis nebulosa* |
| *Parupeneus barberinus* | *Scolopsis aurata* | *Haletta semifasciata* | *Parapercis ramsayi* |
| *Parupeneus ciliatus* | *Scolopsis bilineata* | ***Heteroscarus*** | *Parapercis roseoviridis* |
| *Parupeneus crassilabris* | *Scolopsis bimaculata* | *Heteroscarus acroptilus* | ***Pinguipes*** |
| *Parupeneus cyclostomus* | *Scolopsis ciliata* | ***Neoodax*** | *Pinguipes brasilianus* |
| *Parupeneus heptacanthus* | *Scolopsis frenata* | *Neoodax balteatus* | ***Prolatilus*** |
| *Parupeneus indicus* | *Scolopsis ghanam* | ***Olisthops*** | *Prolatilus jugularis* |
| *Parupeneus macronemus* | *Scolopsis lineata* | *Olisthops cyanomelas* | ***Pseudopercis*** |
| *Parupeneus multifasciatus* | *Scolopsis margaritifera* | ***Siphonognathus*** | *Pseudopercis numida* |
| *Parupeneus pleurostigma* | *Scolopsis monogramma* | *Siphonognathus argyrophanes* | *Pseudopercis semifasciata* |
| *Parupeneus porphyreus* | *Scolopsis taeniata* | *Siphonognathus attenuatus* | **Plesiopidae** |
| *Parupeneus spilurus* | *Scolopsis taenioptera* | *Siphonognathus beddomei* | ***Acanthoclinus*** |
| *Parupeneus trifasciatus* | *Scolopsis temporalis* | *Siphonognathus caninis* | *Acanthoclinus fuscus* |
| ***Pseudupeneus*** | *Scolopsis trilineata* | *Siphonognathus radiatus* | ***Acanthoplesiops*** |
| *Pseudupeneus maculatus* | *Scolopsis vosmeri* | *Siphonognathus tanyourus* | *Acanthoplesiops hiatti* |
| *Pseudupeneus prayensis* | *Scolopsis xenochrous* | **Opistognathidae** | ***Belonepterygion*** |
| ***Upeneus*** | **Nomeidae** | ***Opistognathus*** | *Belonepterygion fasciolatum* |
| *Upeneus moluccensis* | ***Cubiceps*** | *Opistognathus maxillosus* | ***Calloplesiops*** |
| *Upeneus parvus* | *Cubiceps baxteri* | *Opistognathus melachasme* | *Calloplesiops altivelis* |
| *Upeneus sulphureus* | *Cubiceps capensis* | *Opistognathus papuensis* | ***Plesiops*** |
| *Upeneus taeniopterus* | *Cubiceps pauciradiatus* | **Pempheridae** | *Plesiops coeruleolineatus* |
| *Upeneus tragula* | ***Nomeus*** | ***Parapriacanthus*** | *Plesiops corallicola* |
| *Upeneus vittatus* | *Nomeus gronovii* | *Parapriacanthus ransonneti* | **Polynemidae** |
| **Nematistiidae** | ***Psenes*** | ***Pempheris*** | ***Eleutheronema*** |
| ***Nematistius*** | *Psenes arafurensis* | *Pempheris multiradiata* | *Eleutheronema rhadinum* |
| *Nematistius pectoralis* | *Psenes cyanophrys* | *Pempheris oualensis* | *Eleutheronema tetradactylum* |
| **Nemipteridae** | *Psenes maculatus* | *Pempheris vanicolensis* | *Eleutheronema tridactylum* |
| ***Nemipterus*** | *Psenes pellucidus* | **Pentacerotidae** | ***Filimanus*** |
| *Nemipterus aurora* | **Nototheniidae** | ***Evistias*** | *Filimanus heptadactyla* |
| *Nemipterus balinensis* | ***Cryothenia*** | *Evistias acutirostris* | *Filimanus sealei* |
| *Nemipterus balinensoides* | *Cryothenia peninsulae* | ***Paristiopterus*** | *Filimanus xanthonema* |
| *Nemipterus bathybius* | ***Dissostichus*** | *Paristiopterus gallipavo* | ***Galeoides*** |
| *Nemipterus bipunctatus* | *Dissostichus eleginoides* | *Paristiopterus labiosus* | *Galeoides decadactylus* |
| *Nemipterus celebicus* | *Dissostichus mawsoni* | ***Pentaceros*** | ***Leptomelanosoma*** |
| *Nemipterus furcosus* | ***Gobionotothen*** | *Pentaceros richardsoni* | *Leptomelanosoma indicum* |
| *Nemipterus gracilis* | *Gobionotothen acuta* | *Pentaceros wheeleri* | ***Pentanemus*** |
| *Nemipterus hexodon* | *Gobionotothen gibberifrons* | **Percophidae** | *Pentanemus quinquarius* |
| *Nemipterus isacanthus* | *Gobionotothen marionensis* | ***Acanthaphritis*** | ***Polydactylus*** |
| *Nemipterus japonicus* | ***Lepidonotothen*** | *Acanthaphritis barbata* | *Polydactylus approximans* |
| *Nemipterus marginatus* | *Lepidonotothen squamifrons* | ***Bembrops*** | *Polydactylus macrochir* |
| *Nemipterus mesoprion* | ***Lindbergichthys*** | *Bembrops filiferus* | *Polydactylus microstomus* |
| *Nemipterus nematophorus* | *Lindbergichthys mizops* | *Bembrops gobioides* | *Polydactylus mullani* |
| *Nemipterus nematopus* | *Lindbergichthys nudifrons* | *Bembrops greyi* | *Polydactylus multiradiatus* |
| *Nemipterus nemurus* | ***Notothenia*** | *Bembrops heterurus* | *Polydactylus nigripinnis* |
| *Nemipterus peronii* | *Notothenia coriiceps* | ***Chrionema*** | *Polydactylus octonemus* |
| *Nemipterus tambuloides* | *Notothenia rossii* | *Chrionema pallidum* | *Polydactylus oligodon* |
| *Nemipterus theodorei* | ***Nototheniops*** | ***Matsubaraea*** | *Polydactylus opercularis* |
| *Nemipterus thosaporni* | *Nototheniops larseni* | *Matsubaraea fusiforme* | *Polydactylus plebeius* |
| *Nemipterus virgatus* | ***Pagothenia*** | ***Osopsaron*** | *Polydactylus quadrifilis* |
| *Nemipterus vitiensis* | *Pagothenia borchgrevinki* | *Osopsaron karlik* | *Polydactylus sexfilis* |
| *Nemipterus zysron* | *Pagothenia brachysoma* | ***Percophis*** | *Polydactylus sextarius* |

**Table S1 (continued).**

| *Polydactylus virginicus* | *Abudefduf troschelii* | *Chromis leucura* | ***Neopomacentrus*** |
| --- | --- | --- | --- |
| ***Polynemus*** | *Abudefduf vaigiensis* | *Chromis limbata* | *Neopomacentrus anabatoides* |
| *Polynemus paradiseus* | *Abudefduf whitleyi* | *Chromis limbaughi* | *Neopomacentrus azysron* |
| **Polyprionidae** | ***Acanthochromis*** | *Chromis lineata* | *Neopomacentrus cyanomos* |
| ***Polyprion*** | *Acanthochromis polyacanthus* | *Chromis margaritifer* | *Neopomacentrus fuliginosus* |
| *Polyprion americanus* | ***Amblyglyphidodon*** | *Chromis multilineata* | *Neopomacentrus metallicus* |
| *Polyprion oxygeneios* | *Amblyglyphidodon aureus* | *Chromis nigrura* | *Neopomacentrus taeniurus* |
| ***Stereolepis*** | *Amblyglyphidodon curacao* | *Chromis nitida* | *Neopomacentrus violascens* |
| *Stereolepis gigas* | *Amblyglyphidodon flavilatus* | *Chromis pamae* | ***Parma*** |
| **Pomacanthidae** | *Amblyglyphidodon leucogaster* | *Chromis punctipinnis* | *Parma alboscapularis* |
| ***Apolemichthys*** | *Amblyglyphidodon ternatensis* | *Chromis retrofasciata* | *Parma microlepis* |
| *Apolemichthys arcuatus* | ***Amblypomacentrus*** | *Chromis scotti* | ***Plectroglyphidodon*** |
| *Apolemichthys griffisi* | *Amblypomacentrus breviceps* | *Chromis struhsakeri* | *Plectroglyphidodon dickii* |
| *Apolemichthys trimaculatus* | ***Amphiprion*** | *Chromis ternatensis* | *Plectroglyphidodon imparipennis* |
| *Apolemichthys xanthopunctatus* | *Amphiprion akallopisos* | *Chromis vanderbilti* | *Plectroglyphidodon johnstonianus* |
| ***Centropyge*** | *Amphiprion akindynos* | *Chromis verater* | *Plectroglyphidodon lacrymatus* |
| *Centropyge argi* | *Amphiprion allardi* | *Chromis viridis* | *Plectroglyphidodon leucozonus* |
| *Centropyge aurantia* | *Amphiprion bicinctus* | *Chromis weberi* | *Plectroglyphidodon phoenixensis* |
| *Centropyge bicolor* | *Amphiprion chagosensis* | *Chromis woodsi* | ***Pomacentrus*** |
| *Centropyge bispinosa* | *Amphiprion chrysogaster* | *Chromis xanthochira* | *Pomacentrus adelus* |
| *Centropyge colini* | *Amphiprion chrysopterus* | *Chromis xanthura* | *Pomacentrus amboinensis* |
| *Centropyge flavissima* | *Amphiprion clarkii* | ***Chrysiptera*** | *Pomacentrus baenschi* |
| *Centropyge heraldi* | *Amphiprion ephippium* | *Chrysiptera biocellata* | *Pomacentrus bankanensis* |
| *Centropyge loriculus* | *Amphiprion frenatus* | *Chrysiptera brownriggii* | *Pomacentrus brachialis* |
| *Centropyge multicolor* | *Amphiprion fuscocaudatus* | *Chrysiptera caeruleolineata* | *Pomacentrus burroughi* |
| *Centropyge nigriocella* | *Amphiprion latezonatus* | *Chrysiptera cyanea* | *Pomacentrus chrysurus* |
| *Centropyge nox* | *Amphiprion latifasciatus* | *Chrysiptera glauca* | *Pomacentrus coelestis* |
| *Centropyge potteri* | *Amphiprion leucokranos* | *Chrysiptera oxycephala* | *Pomacentrus emarginatus* |
| *Centropyge tibicen* | *Amphiprion mccullochi* | *Chrysiptera parasema* | *Pomacentrus grammorhynchus* |
| *Centropyge vrolikii* | *Amphiprion melanopus* | *Chrysiptera rapanui* | *Pomacentrus lepidogenys* |
| ***Chaetodontoplus*** | *Amphiprion nigripes* | *Chrysiptera rex* | *Pomacentrus moluccensis* |
| *Chaetodontoplus mesoleucus* | *Amphiprion ocellaris* | *Chrysiptera rollandi* | *Pomacentrus nagasakiensis* |
| ***Genicanthus*** | *Amphiprion omanensis* | *Chrysiptera sinclairi* | *Pomacentrus nigromanus* |
| *Genicanthus bellus* | *Amphiprion percula* | *Chrysiptera springeri* | *Pomacentrus opisthostigma* |
| *Genicanthus melanospilos* | *Amphiprion perideraion* | *Chrysiptera starcki* | *Pomacentrus pavo* |
| *Genicanthus personatus* | *Amphiprion polymnus* | *Chrysiptera talboti* | *Pomacentrus philippinus* |
| *Genicanthus watanabei* | *Amphiprion rubrocinctus* | *Chrysiptera traceyi* | *Pomacentrus reidi* |
| ***Holacanthus*** | *Amphiprion sandaracinos* | *Chrysiptera tricincta* | *Pomacentrus simsiang* |
| *Holacanthus bermudensis* | *Amphiprion sebae* | *Chrysiptera unimaculata* | *Pomacentrus stigma* |
| *Holacanthus ciliaris* | *Amphiprion tricinctus* | ***Dascyllus*** | *Pomacentrus sulfureus* |
| *Holacanthus passer* | ***Azurina*** | *Dascyllus albisella* | *Pomacentrus vaiuli* |
| *Holacanthus tricolor* | *Azurina eupalama* | *Dascyllus aruanus* | ***Pomachromis*** |
| ***Paracentropyge*** | *Azurina hirundo* | *Dascyllus melanurus* | *Pomachromis exilis* |
| *Paracentropyge multifasciata* | ***Cheiloprion*** | *Dascyllus reticulatus* | *Pomachromis richardsoni* |
| ***Pomacanthus*** | *Cheiloprion labiatus* | *Dascyllus strasburgi* | ***Premnas*** |
| *Pomacanthus annularis* | ***Chromis*** | *Dascyllus trimaculatus* | *Premnas biaculeatus* |
| *Pomacanthus arcuatus* | *Chromis abyssicola* | ***Dischistodus*** | ***Similiparma*** |
| *Pomacanthus imperator* | *Chromis acares* | *Dischistodus chrysopoecilus* | *Similiparma lurida* |
| *Pomacanthus navarchus* | *Chromis agilis* | *Dischistodus fasciatus* | ***Stegastes*** |
| *Pomacanthus paru* | *Chromis alpha* | *Dischistodus melanotus* | *Stegastes acapulcoensis* |
| *Pomacanthus semicirculatus* | *Chromis alta* | *Dischistodus perspicillatus* | *Stegastes adustus* |
| *Pomacanthus sexstriatus* | *Chromis amboinensis* | *Dischistodus prosopotaenia* | *Stegastes albifasciatus* |
| *Pomacanthus xanthometopon* | *Chromis analis* | *Dischistodus pseudochrysopoecilus* | *Stegastes altus* |
| *Pomacanthus zonipectus* | *Chromis atrilobata* | ***Hemiglyphidodon*** | *Stegastes apicalis* |
| ***Pygoplites*** | *Chromis atripectoralis* | *Hemiglyphidodon plagiometopon* | *Stegastes arcifrons* |
| *Pygoplites diacanthus* | *Chromis atripes* | ***Hypsypops*** | *Stegastes aureus* |
| **Pomacentridae** | *Chromis brevirostris* | *Hypsypops rubicundus* | *Stegastes beebei* |
| ***Abudefduf*** | *Chromis caudalis* | ***Lepidozygus*** | *Stegastes emeryi* |
| *Abudefduf abdominalis* | *Chromis chromis* | *Lepidozygus tapeinosoma* | *Stegastes fasciolatus* |
| *Abudefduf bengalensis* | *Chromis chrysura* | ***Microspathodon*** | *Stegastes flavilatus* |
| *Abudefduf concolor* | *Chromis cinerascens* | *Microspathodon bairdii* | *Stegastes gascoynei* |
| *Abudefduf conformis* | *Chromis cyanea* | *Microspathodon chrysurus* | *Stegastes insularis* |
| *Abudefduf declivifrons* | *Chromis delta* | *Microspathodon dorsalis* | *Stegastes leucorus* |
| *Abudefduf lorenzi* | *Chromis earina* | *Microspathodon frontatus* | *Stegastes leucostictus* |
| *Abudefduf natalensis* | *Chromis elerae* | ***Neoglyphidodon*** | *Stegastes limbatus* |
| *Abudefduf notatus* | *Chromis enchrysura* | *Neoglyphidodon bonang* | *Stegastes nigricans* |
| *Abudefduf saxatilis* | *Chromis flavicauda* | *Neoglyphidodon carlsoni* | *Stegastes obreptus* |
| *Abudefduf septemfasciatus* | *Chromis flavipectoralis* | *Neoglyphidodon crossi* | *Stegastes partitus* |
| *Abudefduf sexfasciatus* | *Chromis flavomaculata* | *Neoglyphidodon melas* | *Stegastes pelicieri* |
| *Abudefduf sordidus* | *Chromis hanui* | *Neoglyphidodon nigroris* | *Stegastes punctatus* |
| *Abudefduf sparoides* | *Chromis hypsilepis* | *Neoglyphidodon polyacanthus* | *Stegastes rectifraenum* |
| *Abudefduf taurus* | *Chromis lepidolepis* | *Neoglyphidodon thoracotaeniatus* | *Stegastes variabilis* |

**Table S1 (continued).**

| **Pomatomidae** | *Chlorurus sordidus* | ***Ctenosciaena*** | *Odontoscion eurymesops* |
| --- | --- | --- | --- |
| ***Pomatomus*** | ***Cryptotomus*** | *Ctenosciaena gracilicirrhus* | *Odontoscion xanthops* |
| *Pomatomus saltatrix* | *Cryptotomus roseus* | *Ctenosciaena peruviana* | ***Ophioscion*** |
| **Priacanthidae** | ***Hipposcarus*** | ***Cynoscion*** | *Ophioscion imiceps* |
| ***Cookeolus*** | *Hipposcarus longiceps* | *Cynoscion acoupa* | *Ophioscion scierus* |
| *Cookeolus japonicus* | ***Leptoscarus*** | *Cynoscion analis* | ***Otolithes*** |
| ***Heteropriacanthus*** | *Leptoscarus vaigiensis* | *Cynoscion arenarius* | *Otolithes ruber* |
| *Heteropriacanthus cruentatus* | ***Nicholsina*** | *Cynoscion guatucupa* | ***Otolithoides*** |
| ***Priacanthus*** | *Nicholsina denticulata* | *Cynoscion jamaicensis* | *Otolithoides biauritus* |
| *Priacanthus alalaua* | *Nicholsina usta* | *Cynoscion leiarchus* | ***Paralonchurus*** |
| *Priacanthus arenatus* | ***Scarus*** | *Cynoscion microlepidotus* | *Paralonchurus goodei* |
| *Priacanthus hamrur* | *Scarus altipinnis* | *Cynoscion nannus* | *Paralonchurus peruanus* |
| *Priacanthus macracanthus* | *Scarus chameleon* | *Cynoscion nebulosus* | ***Pareques*** |
| *Priacanthus tayenus* | *Scarus coelestinus* | *Cynoscion nortoni* | *Pareques acuminatus* |
| ***Pristigenys*** | *Scarus coeruleus* | *Cynoscion nothus* | *Pareques umbrosus* |
| *Pristigenys alta* | *Scarus compressus* | *Cynoscion parvipinnis* | *Pareques viola* |
| *Pristigenys serrula* | *Scarus dimidiatus* | *Cynoscion phoxocephalus* | ***Pennahia*** |
| **Pseudochromidae** | *Scarus festivus* | *Cynoscion regalis* | *Pennahia argentata* |
| ***Anisochromis*** | *Scarus flavipectoralis* | *Cynoscion reticulatus* | ***Pentheroscion*** |
| *Anisochromis kenyae* | *Scarus forsteni* | *Cynoscion similis* | *Pentheroscion mbizi* |
| ***Blennodesmus*** | *Scarus frenatus* | *Cynoscion squamipinnis* | ***Pogonias*** |
| *Blennodesmus scapularis* | *Scarus ghobban* | *Cynoscion steindachneri* | *Pogonias cromis* |
| ***Congrogadus*** | *Scarus globiceps* | *Cynoscion stolzmanni* | ***Protonibea*** |
| *Congrogadus subducens* | *Scarus guacamaia* | *Cynoscion striatus* | *Protonibea diacanthus* |
| ***Halidesmus*** | *Scarus hoefleri* | *Cynoscion virescens* | ***Pseudotolithus*** |
| *Halidesmus scapularis* | *Scarus hypselopterus* | ***Elattarchus*** | *Pseudotolithus elongatus* |
| ***Halimuraena*** | *Scarus iseri* | *Elattarchus archidium* | *Pseudotolithus epipercus* |
| *Halimuraena hexagonata* | *Scarus niger* | ***Equetus*** | *Pseudotolithus moorii* |
| *Halimuraena lepopareia* | *Scarus oviceps* | *Equetus lanceolatus* | *Pseudotolithus senegalensis* |
| *Halimuraena shakai* | *Scarus prasiognathos* | *Equetus punctatus* | *Pseudotolithus senegallus* |
| ***Haliophis*** | *Scarus psittacus* | ***Genyonemus*** | *Pseudotolithus typus* |
| *Haliophis guttatus* | *Scarus quoyi* | *Genyonemus lineatus* | ***Pteroscion*** |
| ***Manonichthys*** | *Scarus rubroviolaceus* | ***Isopisthus*** | *Pteroscion peli* |
| *Manonichthys polynemus* | *Scarus schlegeli* | *Isopisthus parvipinnis* | ***Roncador*** |
| ***Pictichromis*** | *Scarus spinus* | *Isopisthus remifer* | *Roncador stearnsii* |
| *Pictichromis porphyrea* | *Scarus taeniopterus* | ***Johnius*** | ***Sciaena*** |
| ***Pseudochromis*** | *Scarus vetula* | *Johnius borneensis* | *Sciaena umbra* |
| *Pseudochromis ammeri* | ***Sparisoma*** | ***Larimichthys*** | ***Sciaenops*** |
| *Pseudochromis cyanotaenia* | *Sparisoma atomarium* | *Larimichthys crocea* | *Sciaenops ocellatus* |
| *Pseudochromis eichleri* | *Sparisoma aurofrenatum* | *Larimichthys polyactis* | ***Seriphus*** |
| *Pseudochromis fuligifinis* | *Sparisoma chrysopterum* | ***Larimus*** | *Seriphus politus* |
| *Pseudochromis fuscus* | *Sparisoma cretense* | *Larimus acclivis* | ***Stellifer*** |
| *Pseudochromis marshallensis* | *Sparisoma radians* | *Larimus argenteus* | *Stellifer chrysoleuca* |
| *Pseudochromis nigrovittatus* | *Sparisoma rubripinne* | *Larimus breviceps* | *Stellifer ericymba* |
| *Pseudochromis striatus* | *Sparisoma tuiupiranga* | *Larimus effulgens* | *Stellifer illecebrosus* |
| *Pseudochromis tapeinosoma* | *Sparisoma viride* | *Larimus fasciatus* | *Stellifer lanceolatus* |
| ***Pseudoplesiops*** | **Scatophagidae** | *Larimus pacificus* | *Stellifer mancorensis* |
| *Pseudoplesiops howensis* | ***Scatophagus*** | ***Leiostomus*** | *Stellifer minor* |
| *Pseudoplesiops revellei* | *Scatophagus argus* | *Leiostomus xanthurus* | ***Totoaba*** |
| *Pseudoplesiops rosae* | *Scatophagus tetracanthus* | ***Macrodon*** | *Totoaba macdonaldi* |
| *Pseudoplesiops typus* | **Sciaenidae** | *Macrodon ancylodon* | ***Umbrina*** |
| **Ptilichthyidae** | ***Argyrosomus*** | ***Menticirrhus*** | *Umbrina bussingi* |
| ***Ptilichthys*** | *Argyrosomus hololepidotus* | *Menticirrhus americanus* | *Umbrina canariensis* |
| *Ptilichthys goodei* | *Argyrosomus inodorus* | *Menticirrhus elongatus* | *Umbrina canosai* |
| **Rachycentridae** | *Argyrosomus japonicus* | *Menticirrhus littoralis* | *Umbrina cirrosa* |
| ***Rachycentron*** | *Argyrosomus regius* | *Menticirrhus nasus* | *Umbrina coroides* |
| *Rachycentron canadum* | *Argyrosomus thorpei* | *Menticirrhus paitensis* | *Umbrina roncador* |
| **Scaridae** | ***Atractoscion*** | *Menticirrhus panamensis* | *Umbrina ronchus* |
| ***Bolbometopon*** | *Atractoscion aequidens* | *Menticirrhus saxatilis* | *Umbrina xanti* |
| *Bolbometopon muricatum* | *Atractoscion nobilis* | *Menticirrhus undulatus* | **Scombridae** |
| ***Calotomus*** | ***Atrobucca*** | ***Micropogonias*** | ***Acanthocybium*** |
| *Calotomus carolinus* | *Atrobucca nibe* | *Micropogonias altipinnis* | *Acanthocybium solandri* |
| *Calotomus japonicus* | ***Bairdiella*** | *Micropogonias ectenes* | ***Allothunnus*** |
| *Calotomus spinidens* | *Bairdiella ensifera* | *Micropogonias furnieri* | *Allothunnus fallai* |
| ***Cetoscarus*** | *Bairdiella ronchus* | *Micropogonias undulatus* | ***Auxis*** |
| *Cetoscarus bicolor* | ***Cheilotrema*** | ***Miracorvina*** | *Auxis rochei* |
| ***Chlorurus*** | *Cheilotrema saturnum* | *Miracorvina angolensis* | *Auxis thazard* |
| *Chlorurus bleekeri* | ***Cilus*** | ***Nibea*** | ***Cybiosarda*** |
| *Chlorurus bowersi* | *Cilus gilberti* | *Nibea albiflora* | *Cybiosarda elegans* |
| *Chlorurus frontalis* | ***Corvula*** | *Nibea soldado* | ***Euthynnus*** |
| *Chlorurus japanensis* | *Corvula macrops* | ***Odontoscion*** | *Euthynnus affinis* |
| *Chlorurus microrhinos* | *Corvula sanctaeluciae* | *Odontoscion dentex* | *Euthynnus alletteratus* |

**Table S1 (continued).**

| *Euthynnus lineatus* | *Anthias anthias* | *Epinephelus bleekeri* | *Hemanthias leptus* |
| --- | --- | --- | --- |
| ***Gasterochisma*** | *Anthias asperilinguis* | *Epinephelus bontoides* | *Hemanthias peruanus* |
| *Gasterochisma melampus* | *Anthias menezesi* | *Epinephelus bruneus* | *Hemanthias signifer* |
| ***Grammatorcynus*** | *Anthias nicholsi* | *Epinephelus caninus* | ***Hypoplectrus*** |
| *Grammatorcynus bicarinatus* | *Anthias noeli* | *Epinephelus chabaudi* | *Hypoplectrus nigricans* |
| *Grammatorcynus bilineatus* | ***Anyperodon*** | *Epinephelus chlorostigma* | *Hypoplectrus unicolor* |
| ***Gymnosarda*** | *Anyperodon leucogrammicus* | *Epinephelus cifuentesi* | ***Hyporthodus*** |
| *Gymnosarda unicolor* | ***Aulacocephalus*** | *Epinephelus coeruleopunctatus* | *Hyporthodus acanthistius* |
| ***Katsuwonus*** | *Aulacocephalus temminckii* | *Epinephelus coioides* | *Hyporthodus ergastularius* |
| *Katsuwonus pelamis* | ***Baldwinella*** | *Epinephelus corallicola* | *Hyporthodus flavolimbatus* |
| ***Orcynopsis*** | *Baldwinella aureorubens* | *Epinephelus costae* | *Hyporthodus haifensis* |
| *Orcynopsis unicolor* | *Baldwinella vivanus* | *Epinephelus cyanopodus* | *Hyporthodus mystacinus* |
| ***Rastrelliger*** | ***Bathyanthias*** | *Epinephelus daemelii* | *Hyporthodus nigritus* |
| *Rastrelliger brachysoma* | *Bathyanthias mexicanus* | *Epinephelus diacanthus* | *Hyporthodus niphobles* |
| *Rastrelliger faughni* | ***Belonoperca*** | *Epinephelus drummondhayi* | *Hyporthodus niveatus* |
| *Rastrelliger kanagurta* | *Belonoperca chabanaudi* | *Epinephelus erythrurus* | *Hyporthodus octofasciatus* |
| ***Sarda*** | ***Centropristis*** | *Epinephelus fasciatomaculosus* | *Hyporthodus quernus* |
| *Sarda australis* | *Centropristis ocyurus* | *Epinephelus fasciatus* | *Hyporthodus septemfasciatus* |
| *Sarda chiliensis* | *Centropristis philadelphica* | *Epinephelus faveatus* | ***Liopropoma*** |
| *Sarda lineolata* | *Centropristis striata* | *Epinephelus flavocaeruleus* | *Liopropoma africanum* |
| *Sarda orientalis* | ***Cephalopholis*** | *Epinephelus fuscoguttatus* | *Liopropoma carmabi* |
| *Sarda sarda* | *Cephalopholis aitha* | *Epinephelus goreensis* | *Liopropoma eukrines* |
| ***Scomber*** | *Cephalopholis argus* | *Epinephelus heniochus* | *Liopropoma fasciatum* |
| *Scomber australasicus* | *Cephalopholis aurantia* | *Epinephelus hexagonatus* | *Liopropoma mitratum* |
| *Scomber japonicus* | *Cephalopholis boenak* | *Epinephelus howlandi* | *Liopropoma mowbrayi* |
| *Scomber scombrus* | *Cephalopholis cruentata* | *Epinephelus itajara* | *Liopropoma multilineatum* |
| ***Scomberomorus*** | *Cephalopholis cyanostigma* | *Epinephelus lanceolatus* | *Liopropoma pallidum* |
| *Scomberomorus brasiliensis* | *Cephalopholis formosa* | *Epinephelus latifasciatus* | *Liopropoma susumi* |
| *Scomberomorus cavalla* | *Cephalopholis fulva* | *Epinephelus longispinis* | *Liopropoma tonstrinum* |
| *Scomberomorus commerson* | *Cephalopholis hemistiktos* | *Epinephelus macrospilos* | ***Luzonichthys*** |
| *Scomberomorus concolor* | *Cephalopholis igarashiensis* | *Epinephelus maculatus* | *Luzonichthys waitei* |
| *Scomberomorus guttatus* | *Cephalopholis leopardus* | *Epinephelus magniscuttis* | *Luzonichthys whitleyi* |
| *Scomberomorus koreanus* | *Cephalopholis microprion* | *Epinephelus malabaricus* | ***Mycteroperca*** |
| *Scomberomorus lineolatus* | *Cephalopholis miniata* | *Epinephelus marginatus* | *Mycteroperca acutirostris* |
| *Scomberomorus maculatus* | *Cephalopholis nigri* | *Epinephelus melanostigma* | *Mycteroperca fusca* |
| *Scomberomorus munroi* | *Cephalopholis nigripinnis* | *Epinephelus merra* | *Mycteroperca interstitialis* |
| *Scomberomorus niphonius* | *Cephalopholis oligosticta* | *Epinephelus miliaris* | *Mycteroperca jordani* |
| *Scomberomorus plurilineatus* | *Cephalopholis panamensis* | *Epinephelus morio* | *Mycteroperca microlepis* |
| *Scomberomorus queenslandicus* | *Cephalopholis polleni* | *Epinephelus morrhua* | *Mycteroperca olfax* |
| *Scomberomorus regalis* | *Cephalopholis polyspila* | *Epinephelus multinotatus* | *Mycteroperca phenax* |
| *Scomberomorus semifasciatus* | *Cephalopholis sexmaculata* | *Epinephelus ongus* | *Mycteroperca prionura* |
| *Scomberomorus sierra* | *Cephalopholis sonnerati* | *Epinephelus poecilonotus* | *Mycteroperca rosacea* |
| *Scomberomorus sinensis* | *Cephalopholis spiloparaea* | *Epinephelus polylepis* | *Mycteroperca rubra* |
| *Scomberomorus tritor* | *Cephalopholis taeniops* | *Epinephelus polyphekadion* | *Mycteroperca tigris* |
| ***Thunnus*** | *Cephalopholis urodeta* | *Epinephelus posteli* | *Mycteroperca venenosa* |
| *Thunnus alalunga* | ***Cromileptes*** | *Epinephelus quoyanus* | *Mycteroperca xenarcha* |
| *Thunnus albacares* | *Cromileptes altivelis* | *Epinephelus radiatus* | ***Niphon*** |
| *Thunnus atlanticus* | ***Dermatolepis*** | *Epinephelus retouti* | *Niphon spinosus* |
| *Thunnus maccoyii* | *Dermatolepis dermatolepis* | *Epinephelus rivulatus* | ***Paralabrax*** |
| *Thunnus obesus* | *Dermatolepis inermis* | *Epinephelus sexfasciatus* | *Paralabrax auroguttatus* |
| *Thunnus orientalis* | *Dermatolepis striolata* | *Epinephelus socialis* | *Paralabrax clathratus* |
| *Thunnus thynnus* | ***Diplectrum*** | *Epinephelus spilotoceps* | *Paralabrax humeralis* |
| *Thunnus tonggol* | *Diplectrum bivittatum* | *Epinephelus stictus* | *Paralabrax loro* |
| **Scombrolabracidae** | *Diplectrum eumelum* | *Epinephelus stoliczkae* | *Paralabrax maculatofasciatus* |
| ***Scombrolabrax*** | *Diplectrum euryplectrum* | *Epinephelus striatus* | *Paralabrax nebulifer* |
| *Scombrolabrax heterolepis* | *Diplectrum formosum* | *Epinephelus summana* | ***Paranthias*** |
| **Scombropidae** | *Diplectrum labarum* | *Epinephelus tauvina* | *Paranthias colonus* |
| ***Scombrops*** | *Diplectrum macropoma* | *Epinephelus timorensis* | *Paranthias furcifer* |
| *Scombrops oculatus* | *Diplectrum pacificum* | *Epinephelus trimaculatus* | ***Plectranthias*** |
| **Serranidae** | *Diplectrum rostrum* | *Epinephelus tuamotuensis* | *Plectranthias alleni* |
| ***Acanthistius*** | *Diplectrum sciurus* | *Epinephelus tukula* | *Plectranthias exsul* |
| *Acanthistius brasilianus* | ***Diploprion*** | *Epinephelus undulatostriatus* | *Plectranthias fourmanoiri* |
| *Acanthistius ocellatus* | *Diploprion bifasciatum* | *Epinephelus undulosus* | *Plectranthias garrupellus* |
| *Acanthistius paxtoni* | ***Epinephelus*** | ***Gonioplectrus*** | *Plectranthias kamii* |
| *Acanthistius sebastoides* | *Epinephelus adscensionis* | *Gonioplectrus hispanus* | *Plectranthias longimanus* |
| *Acanthistius serratus* | *Epinephelus aeneus* | ***Gracila*** | *Plectranthias nanus* |
| ***Aethaloperca*** | *Epinephelus akaara* | *Gracila albomarginata* | *Plectranthias robertsi* |
| *Aethaloperca rogaa* | *Epinephelus albomarginatus* | ***Grammistes*** | *Plectranthias winniensis* |
| ***Alphestes*** | *Epinephelus amblycephalus* | *Grammistes sexlineatus* | ***Plectropomus*** |
| *Alphestes afer* | *Epinephelus areolatus* | ***Grammistops*** | *Plectropomus areolatus* |
| *Alphestes immaculatus* | *Epinephelus awoara* | *Grammistops ocellatus* | *Plectropomus laevis* |
| ***Anthias*** | *Epinephelus bilobatus* | ***Hemanthias*** | *Plectropomus leopardus* |

**Table S1 (continued).**

| *Plectropomus maculatus* | *Siganus punctatissimus* | *Chrysoblephus cristiceps* | *Sphyraena acutipinnis* |
| --- | --- | --- | --- |
| *Plectropomus oligacanthus* | *Siganus punctatus* | *Chrysoblephus gibbiceps* | *Sphyraena afra* |
| *Plectropomus pessuliferus* | *Siganus randalli* | ***Cymatoceps*** | *Sphyraena argentea* |
| *Plectropomus punctatus* | *Siganus spinus* | *Cymatoceps nasutus* | *Sphyraena barracuda* |
| ***Pogonoperca*** | *Siganus stellatus* | ***Dentex*** | *Sphyraena borealis* |
| *Pogonoperca punctata* | *Siganus sutor* | *Dentex angolensis* | *Sphyraena forsteri* |
| ***Pronotogrammus*** | *Siganus unimaculatus* | *Dentex barnardi* | *Sphyraena helleri* |
| *Pronotogrammus eos* | *Siganus vermiculatus* | *Dentex canariensis* | *Sphyraena idiastes* |
| *Pronotogrammus martinicensis* | *Siganus virgatus* | *Dentex congoensis* | *Sphyraena jello* |
| *Pronotogrammus multifasciatus* | *Siganus vulpinus* | *Dentex dentex* | *Sphyraena novaehollandiae* |
| ***Pseudanthias*** | **Sillaginidae** | *Dentex gibbosus* | *Sphyraena obtusata* |
| *Pseudanthias bartlettorum* | ***Sillaginodes*** | *Dentex macrophthalmus* | *Sphyraena picudilla* |
| *Pseudanthias bicolor* | *Sillaginodes punctatus* | *Dentex maroccanus* | *Sphyraena qenie* |
| *Pseudanthias cooperi* | ***Sillaginopodys*** | *Dentex tumifrons* | *Sphyraena sphyraena* |
| *Pseudanthias dispar* | *Sillaginopodys chondropus* | ***Diplodus*** | *Sphyraena tome* |
| *Pseudanthias huchtii* | ***Sillaginops*** | *Diplodus annularis* | **Stichaeidae** |
| *Pseudanthias hypselosoma* | *Sillaginops macrolepis* | *Diplodus argenteus* | ***Alectridium*** |
| *Pseudanthias lori* | ***Sillaginopsis*** | *Diplodus bellottii* | *Alectridium aurantiacum* |
| *Pseudanthias pascalus* | *Sillaginopsis panijus* | *Diplodus cervinus* | ***Anisarchus*** |
| *Pseudanthias pleurotaenia* | ***Sillago*** | *Diplodus holbrookii* | *Anisarchus medius* |
| *Pseudanthias randalli* | *Sillago aeolus* | *Diplodus puntazzo* | ***Anoplarchus*** |
| *Pseudanthias smithvanizi* | *Sillago analis* | *Diplodus sargus* | *Anoplarchus purpurescens* |
| *Pseudanthias squamipinnis* | *Sillago arabica* | *Diplodus vulgaris* | ***Cebidichthys*** |
| *Pseudanthias tuka* | *Sillago asiatica* | ***Evynnis*** | *Cebidichthys violaceus* |
| *Pseudanthias ventralis* | *Sillago bassensis* | *Evynnis cardinalis* | ***Chirolophis*** |
| ***Pseudogramma*** | *Sillago burrus* | ***Lagodon*** | *Chirolophis ascanii* |
| *Pseudogramma gregoryi* | *Sillago ciliata* | *Lagodon rhomboides* | ***Eumesogrammus*** |
| *Pseudogramma polyacantha* | *Sillago flindersi* | ***Lithognathus*** | *Eumesogrammus praecisus* |
| *Pseudogramma thaumasia* | *Sillago indica* | *Lithognathus lithognathus* | ***Leptoclinus*** |
| ***Rypticus*** | *Sillago ingenuua* | *Lithognathus mormyrus* | *Leptoclinus maculatus* |
| *Rypticus maculatus* | *Sillago intermedius* | ***Oblada*** | ***Lumpenella*** |
| *Rypticus saponaceus* | *Sillago japonica* | *Oblada melanura* | *Lumpenella longirostris* |
| ***Saloptia*** | *Sillago lutea* | ***Pachymetopon*** | ***Lumpenus*** |
| *Saloptia powelli* | *Sillago maculata* | *Pachymetopon aeneum* | *Lumpenus fabricii* |
| ***Serraniculus*** | *Sillago parvisquamis* | ***Pagellus*** | *Lumpenus lampretaeformis* |
| *Serraniculus pumilio* | *Sillago robusta* | *Pagellus acarne* | *Lumpenus sagitta* |
| ***Serranocirrhitus*** | *Sillago schomburgkii* | *Pagellus bellottii* | ***Opisthocentrus*** |
| *Serranocirrhitus latus* | *Sillago sihama* | *Pagellus bogaraveo* | *Opisthocentrus ocellatus* |
| ***Serranus*** | *Sillago vincenti* | *Pagellus erythrinus* | ***Phytichthys*** |
| *Serranus annularis* | *Sillago vittata* | ***Pagrus*** | *Phytichthys chirus* |
| *Serranus atricauda* | **Sparidae** | *Pagrus africanus* | ***Plectobranchus*** |
| *Serranus atrobranchus* | ***Acanthopagrus*** | *Pagrus auriga* | *Plectobranchus evides* |
| *Serranus baldwini* | *Acanthopagrus australis* | *Pagrus caeruleostictus* | ***Stichaeus*** |
| *Serranus cabrilla* | *Acanthopagrus berda* | *Pagrus major* | *Stichaeus punctatus punctatus* |
| *Serranus chionaraia* | *Acanthopagrus bifasciatus* | *Pagrus pagrus* | ***Ulvaria*** |
| *Serranus flaviventris* | *Acanthopagrus butcheri* | ***Petrus*** | *Ulvaria subbifurcata* |
| *Serranus hepatus* | *Acanthopagrus latus* | *Petrus rupestris* | ***Xiphister*** |
| *Serranus phoebe* | *Acanthopagrus schlegelii* | ***Polysteganus*** | *Xiphister atropurpureus* |
| *Serranus psittacinus* | ***Archosargus*** | *Polysteganus coeruleopunctatus* | *Xiphister mucosus* |
| *Serranus scriba* | *Archosargus probatocephalus* | *Polysteganus praeorbitalis* | **Stromateidae** |
| *Serranus subligarius* | *Archosargus rhomboidalis* | ***Pterogymnus*** | ***Pampus*** |
| *Serranus tabacarius* | ***Argyrops*** | *Pterogymnus laniarius* | *Pampus argenteus* |
| *Serranus tigrinus* | *Argyrops spinifer* | ***Rhabdosargus*** | *Pampus chinensis* |
| ***Triso*** | ***Argyrozona*** | *Rhabdosargus globiceps* | ***Peprilus*** |
| *Triso dermopterus* | *Argyrozona argyrozona* | *Rhabdosargus sarba* | *Peprilus burti* |
| ***Variola*** | ***Boops*** | *Rhabdosargus thorpei* | *Peprilus medius* |
| *Variola albimarginata* | *Boops boops* | ***Sarpa*** | *Peprilus simillimus* |
| *Variola louti* | ***Calamus*** | *Sarpa salpa* | *Peprilus triacanthus* |
| **Siganidae** | *Calamus arctifrons* | ***Sparidentex*** | ***Stromateus*** |
| ***Siganus*** | *Calamus brachysomus* | *Sparidentex hasta* | *Stromateus fiatola* |
| *Siganus argenteus* | *Calamus calamus* | ***Spicara*** | **Symphysanodontidae** |
| *Siganus canaliculatus* | *Calamus leucosteus* | *Spicara maena* | ***Symphysanodon*** |
| *Siganus corallinus* | *Calamus nodosus* | *Spicara melanurus* | *Symphysanodon maunaloae* |
| *Siganus doliatus* | *Calamus penna* | *Spicara smaris* | **Terapontidae** |
| *Siganus fuscescens* | *Calamus pennatula* | ***Spondyliosoma*** | ***Mesopristes*** |
| *Siganus guttatus* | *Calamus proridens* | *Spondyliosoma cantharus* | *Mesopristes argenteus* |
| *Siganus javus* | ***Centracanthus*** | *Spondyliosoma emarginatum* | *Mesopristes cancellatus* |
| *Siganus labyrinthodes* | *Centracanthus cirrus* | ***Stenotomus*** | ***Rhynchopelates*** |
| *Siganus lineatus* | ***Cheimerius*** | *Stenotomus caprinus* | *Rhynchopelates oxyrhynchus* |
| *Siganus luridus* | *Cheimerius nufar* | *Stenotomus chrysops* | ***Terapon*** |
| *Siganus magnificus* | ***Chrysoblephus*** | **Sphyraenidae** | *Terapon jarbua* |
| *Siganus puellus* | *Chrysoblephus anglicus* | ***Sphyraena*** | *Terapon theraps* |

**Table S1 (continued).**

| **Tetragonuridae** | *Astroscopus guttatus* | **Achiridae** | *Cynoglossus lida* |
| --- | --- | --- | --- |
| ***Tetragonurus*** | *Astroscopus sexspinosus* | ***Achirus*** | *Cynoglossus lingua* |
| *Tetragonurus atlanticus* | *Astroscopus y-graecum* | *Achirus klunzingeri* | *Cynoglossus monodi* |
| **Trachinidae** | ***Kathetostoma*** | *Achirus lineatus* | *Cynoglossus senegalensis* |
| ***Echiichthys*** | *Kathetostoma albigutta* | *Achirus mazatlanus* | *Cynoglossus sinusarabici* |
| *Echiichthys vipera* | *Kathetostoma averruncus* | *Achirus scutum* | *Cynoglossus zanzibarensis* |
| ***Trachinus*** | *Kathetostoma giganteum* | ***Gymnachirus*** | ***Paraplagusia*** |
| *Trachinus araneus* | ***Uranoscopus*** | *Gymnachirus melas* | *Paraplagusia bilineata* |
| *Trachinus draco* | *Uranoscopus albesca* | ***Trinectes*** | ***Symphurus*** |
| **Trichiuridae** | *Uranoscopus scaber* | *Trinectes fimbriatus* | *Symphurus arawak* |
| ***Aphanopus*** | *Uranoscopus sulphureus* | *Trinectes fluviatilis* | *Symphurus atramentatus* |
| *Aphanopus capricornis* | ***Xenocephalus*** | *Trinectes fonsecensis* | *Symphurus atricaudus* |
| *Aphanopus carbo* | *Xenocephalus egregius* | *Trinectes maculatus* | *Symphurus callopterus* |
| *Aphanopus intermedius* | **Xiphiidae** | *Trinectes paulistanus* | *Symphurus caribbeanus* |
| *Aphanopus microphthalmus* | ***Xiphias*** | **Achiropsettidae** | *Symphurus chabanaudi* |
| *Aphanopus mikhailini* | *Xiphias gladius* | ***Achiropsetta*** | *Symphurus civitatium* |
| ***Assurger*** | **Zanclidae** | *Achiropsetta tricholepis* | *Symphurus diomedeanus* |
| *Assurger anzac* | ***Zanclus*** | ***Mancopsetta*** | *Symphurus fasciolaris* |
| ***Benthodesmus*** | *Zanclus cornutus* | *Mancopsetta maculata* | *Symphurus ginsburgi* |
| *Benthodesmus elongatus* | **Zaproridae** | ***Pseudomancopsetta*** | *Symphurus gorgonae* |
| *Benthodesmus macrophthalmus* | ***Zaprora*** | *Pseudomancopsetta andriashevi* | *Symphurus insularis* |
| *Benthodesmus neglectus* | *Zaprora silenus* | **Bothidae** | *Symphurus jenynsi* |
| *Benthodesmus oligoradiatus* | **Zoarcidae** | ***Arnoglossus*** | *Symphurus leei* |
| *Benthodesmus pacificus* | ***Austrolycus*** | *Arnoglossus aspilos* | *Symphurus ligulatus* |
| *Benthodesmus papua* | *Austrolycus laticinctus* | *Arnoglossus capensis* | *Symphurus marginatus* |
| *Benthodesmus simonyi* | ***Dieidolycus*** | *Arnoglossus debilis* | *Symphurus melanurus* |
| *Benthodesmus suluensis* | *Dieidolycus leptodermatus* | *Arnoglossus imperialis* | *Symphurus melasmatotheca* |
| *Benthodesmus tenuis* | ***Gymnelus*** | *Arnoglossus kessleri* | *Symphurus minor* |
| *Benthodesmus tuckeri* | *Gymnelus viridis* | *Arnoglossus laterna* | *Symphurus nebulosus* |
| *Benthodesmus vityazi* | ***Iluocoetes*** | *Arnoglossus rueppelii* | *Symphurus nigrescens* |
| ***Eupleurogrammus*** | *Iluocoetes fimbriatus* | *Arnoglossus thori* | *Symphurus oculellus* |
| *Eupleurogrammus glossodon* | ***Lycenchelys*** | ***Asterorhombus*** | *Symphurus oligomerus* |
| *Eupleurogrammus muticus* | *Lycenchelys antarctica* | *Asterorhombus intermedius* | *Symphurus ommaspilus* |
| ***Evoxymetopon*** | *Lycenchelys aratrirostris* | ***Bothus*** | *Symphurus parvus* |
| *Evoxymetopon taeniatus* | *Lycenchelys bachmanni* | *Bothus guibei* | *Symphurus pelicanus* |
| ***Lepidopus*** | *Lycenchelys bellingshauseni* | *Bothus leopardinus* | *Symphurus piger* |
| *Lepidopus caudatus* | *Lycenchelys crotalinus* | *Bothus lunatus* | *Symphurus plagiusa* |
| *Lepidopus dubius* | *Lycenchelys hureaui* | *Bothus mancus* | *Symphurus plagusia* |
| *Lepidopus fitchi* | *Lycenchelys sarsii* | *Bothus myriaster* | *Symphurus prolatinaris* |
| ***Lepturacanthus*** | *Lycenchelys verrillii* | *Bothus ocellatus* | *Symphurus pusillus* |
| *Lepturacanthus savala* | ***Lycodapus*** | *Bothus pantherinus* | *Symphurus stigmosus* |
| ***Tentoriceps*** | *Lycodapus antarcticus* | *Bothus podas* | *Symphurus tessellatus* |
| *Tentoriceps cristatus* | *Lycodapus pachysoma* | *Bothus robinsi* | *Symphurus thermophilus* |
| ***Trichiurus*** | ***Lycodes*** | ***Chascanopsetta*** | *Symphurus trewavasae* |
| *Trichiurus auriga* | *Lycodes eudipleurostictus* | *Chascanopsetta lugubris* | *Symphurus undecimplerus* |
| *Trichiurus lepturus* | *Lycodes lavalaei* | ***Engyophrys*** | *Symphurus urospilus* |
| **Trichodontidae** | *Lycodes pallidus* | *Engyophrys sanctilaurentii* | *Symphurus varius* |
| ***Arctoscopus*** | *Lycodes reticulatus* | ***Grammatobothus*** | *Symphurus williamsi* |
| *Arctoscopus japonicus* | *Lycodes turneri* | *Grammatobothus pennatus* | **Paralichthyidae** |
| ***Trichodon*** | ***Lycodichthys*** | ***Monolene*** | ***Ancylopsetta*** |
| *Trichodon trichodon* | *Lycodichthys antarcticus* | *Monolene antillarum* | *Ancylopsetta dendritica* |
| **Tripterygiidae** | *Lycodichthys dearborni* | *Monolene asaedae* | *Ancylopsetta dilecta* |
| ***Acanthanectes*** | ***Melanostigma*** | *Monolene dubiosa* | *Ancylopsetta ommata* |
| *Acanthanectes hystrix* | *Melanostigma atlanticum* | *Monolene maculipinna* | ***Citharichthys*** |
| *Acanthanectes rufus* | *Melanostigma gelatinosum* | *Monolene mertensi* | *Citharichthys arctifrons* |
| ***Enneapterygius*** | *Melanostigma pammelas* | *Monolene microstoma* | *Citharichthys cornutus* |
| *Enneapterygius atrogulare* | *Melanostigma vitiazi* | *Monolene sessilicauda* | *Citharichthys gilberti* |
| *Enneapterygius hemimelas* | ***Ophthalmolycus*** | ***Perissias*** | *Citharichthys gymnorhinus* |
| *Enneapterygius minutus* | *Ophthalmolycus amberensis* | *Perissias taeniopterus* | *Citharichthys macrops* |
| *Enneapterygius nanus* | *Ophthalmolycus bothriocephalus* | ***Trichopsetta*** | *Citharichthys platophrys* |
| *Enneapterygius qirmiz* | ***Pachycara*** | *Trichopsetta caribbaea* | *Citharichthys sordidus* |
| *Enneapterygius similis* | *Pachycara brachycephalum* | *Trichopsetta ventralis* | *Citharichthys spilopterus* |
| ***Helcogramma*** | *Pachycara bulbiceps* | **Citharidae** | *Citharichthys stampflii* |
| *Helcogramma hudsoni* | *Pachycara crassiceps* | ***Citharoides*** | *Citharichthys stigmaeus* |
| *Helcogramma striata* | ***Puzanovia*** | *Citharoides macrolepis* | *Citharichthys xanthostigma* |
| ***Norfolkia*** | *Puzanovia rubra* | **Cynoglossidae** | ***Cyclopsetta*** |
| *Norfolkia brachylepis* | ***Seleniolycus*** | ***Cynoglossus*** | *Cyclopsetta chittendeni* |
| ***Tripterygion*** | *Seleniolycus laevifasciatus* | *Cynoglossus acutirostris* | *Cyclopsetta fimbriata* |
| *Tripterygion delaisi* | ***Zoarces*** | *Cynoglossus arel* | *Cyclopsetta panamensis* |
| *Tripterygion tripteronotum* | *Zoarces americanus* | *Cynoglossus browni* | *Cyclopsetta querna* |
| **Uranoscopidae** | *Zoarces viviparus* | *Cynoglossus cadenati* | ***Etropus*** |
| ***Astroscopus*** | **Pleuronectiformes** | *Cynoglossus canariensis* | *Etropus crossotus* |

**Table S1 (continued).**

| *Etropus microstomus* | ***Nematops*** | ***Heteromycteris*** | *Agonopsis vulsa* |
| --- | --- | --- | --- |
| *Etropus rimosus* | *Nematops macrochirus* | *Heteromycteris capensis* | ***Agonus*** |
| ***Gastropsetta*** | ***Oncopterus*** | *Heteromycteris proboscideus* | *Agonus cataphractus* |
| *Gastropsetta frontalis* | *Oncopterus darwinii* | ***Microchirus*** | ***Aspidophoroides*** |
| ***Hippoglossina*** | ***Paralichthodes*** | *Microchirus azevia* | *Aspidophoroides monopterygius* |
| *Hippoglossina bollmani* | *Paralichthodes algoensis* | *Microchirus boscanion* | *Aspidophoroides olrikii* |
| *Hippoglossina oblonga* | ***Parophrys*** | *Microchirus frechkopi* | ***Bathyagonus*** |
| *Hippoglossina stomata* | *Parophrys vetulus* | *Microchirus ocellatus* | *Bathyagonus alascanus* |
| *Hippoglossina tetrophthalma* | ***Platichthys*** | *Microchirus theophila* | *Bathyagonus infraspinatus* |
| ***Paralichthys*** | *Platichthys flesus* | *Microchirus variegatus* | *Bathyagonus nigripinnis* |
| *Paralichthys adspersus* | *Platichthys stellatus* | *Microchirus wittei* | *Bathyagonus pentacanthus* |
| *Paralichthys aestuarius* | ***Pleuronectes*** | ***Monochirus*** | ***Bothragonus*** |
| *Paralichthys albigutta* | *Pleuronectes platessa* | *Monochirus hispidus* | *Bothragonus swanii* |
| *Paralichthys brasiliensis* | *Pleuronectes putnami* | ***Pardachirus*** | ***Hypsagonus*** |
| *Paralichthys californicus* | *Pleuronectes quadrituberculatus* | *Pardachirus pavoninus* | *Hypsagonus quadricornis* |
| *Paralichthys dentatus* | ***Pleuronichthys*** | ***Pegusa*** | ***Leptagonus*** |
| *Paralichthys isosceles* | *Pleuronichthys ocellatus* | *Pegusa impar* | *Leptagonus decagonus* |
| *Paralichthys lethostigma* | ***Poecilopsetta*** | *Pegusa lascaris* | ***Occella*** |
| *Paralichthys olivaceus* | *Poecilopsetta beanii* | *Pegusa nasuta* | *Occella dodecaedron* |
| *Paralichthys orbignyanus* | *Poecilopsetta inermis* | *Pegusa triophthalma* | ***Odontopyxis*** |
| *Paralichthys squamilentus* | ***Psettichthys*** | ***Solea*** | *Odontopyxis trispinosa* |
| *Paralichthys triocellatus* | *Psettichthys melanostictus* | *Solea elongata* | ***Sarritor*** |
| *Paralichthys woolmani* | ***Pseudopleuronectes*** | *Solea senegalensis* | *Sarritor frenatus* |
| ***Pseudorhombus*** | *Pseudopleuronectes americanus* | *Solea solea* | ***Stellerina*** |
| *Pseudorhombus cinnamoneus* | *Pseudopleuronectes herzensteini* | ***Soleichthys*** | *Stellerina xyosterna* |
| *Pseudorhombus elevatus* | ***Reinhardtius*** | *Soleichthys heterorhinos* | ***Xeneretmus*** |
| ***Syacium*** | *Reinhardtius hippoglossoides* | *Soleichthys microcephalus* | *Xeneretmus latifrons* |
| *Syacium latifrons* | ***Taratretis*** | ***Synapturichthys*** | *Xeneretmus triacanthus* |
| *Syacium ovale* | *Taratretis derwentensis* | *Synapturichthys kleinii* | **Anoplopomatidae** |
| *Syacium papillosum* | **Psettodidae** | ***Vanstraelenia*** | ***Anoplopoma*** |
| ***Thysanopsetta*** | ***Psettodes*** | *Vanstraelenia chirophthalma* | *Anoplopoma fimbria* |
| *Thysanopsetta naresi* | *Psettodes belcheri* | **Polymixiiformes** | ***Erilepis*** |
| ***Xystreurys*** | *Psettodes bennettii* | **Polymixiidae** | *Erilepis zonifer* |
| *Xystreurys liolepis* | *Psettodes erumei* | ***Polymixia*** | **Aploactinidae** |
| **Pleuronectidae** | **Samaridae** | *Polymixia japonica* | ***Aploactisoma*** |
| ***Acanthopsetta*** | ***Samaris*** | *Polymixia lowei* | *Aploactisoma milesii* |
| *Acanthopsetta nadeshnyi* | *Samaris cristatus* | **Saccopharyngiformes** | ***Kanekonia*** |
| ***Atheresthes*** | ***Samariscus*** | **Saccopharyngidae** | *Kanekonia queenslandica* |
| *Atheresthes evermanni* | *Samariscus triocellatus* | ***Saccopharynx*** | ***Pseudopataecus*** |
| *Atheresthes stomias* | **Scophthalmidae** | *Saccopharynx ampullaceus* | *Pseudopataecus carnatobarbatus* |
| ***Clidoderma*** | ***Lepidorhombus*** | **Salmoniformes** | **Congiopodidae** |
| *Clidoderma asperrimum* | *Lepidorhombus boscii* | **Salmonidae** | ***Congiopodus*** |
| ***Embassichthys*** | *Lepidorhombus whiffiagonis* | ***Coregonus*** | *Congiopodus peruvianus* |
| *Embassichthys bathybius* | ***Phrynorhombus*** | *Coregonus albula* | *Congiopodus spinifer* |
| ***Eopsetta*** | *Phrynorhombus norvegicus* | *Coregonus autumnalis* | ***Zanclorhynchus*** |
| *Eopsetta jordani* | ***Scophthalmus*** | *Coregonus maraena* | *Zanclorhynchus spinifer* |
| ***Glyptocephalus*** | *Scophthalmus aquosus* | *Coregonus sardinella* | **Cottidae** |
| *Glyptocephalus cynoglossus* | *Scophthalmus maximus* | ***Oncorhynchus*** | ***Archistes*** |
| *Glyptocephalus stelleri* | *Scophthalmus rhombus* | *Oncorhynchus clarkii* | *Archistes biseriatus* |
| *Glyptocephalus zachirus* | ***Zeugopterus*** | *Oncorhynchus gorbuscha* | ***Artediellus*** |
| ***Hippoglossoides*** | *Zeugopterus punctatus* | *Oncorhynchus keta* | *Artediellus atlanticus* |
| *Hippoglossoides dubius* | *Zeugopterus regius* | *Oncorhynchus kisutch* | *Artediellus uncinatus* |
| *Hippoglossoides elassodon* | **Soleidae** | *Oncorhynchus masou* | ***Artedius*** |
| *Hippoglossoides platessoides* | ***Aseraggodes*** | *Oncorhynchus mykiss* | *Artedius fenestralis* |
| ***Hippoglossus*** | *Aseraggodes heemstrai* | *Oncorhynchus nerka* | *Artedius harringtoni* |
| *Hippoglossus hippoglossus* | *Aseraggodes melanostictus* | *Oncorhynchus tshawytscha* | *Artedius lateralis* |
| *Hippoglossus stenolepis* | *Aseraggodes normani* | ***Parahucho*** | *Artedius notospilotus* |
| ***Isopsetta*** | *Aseraggodes whitakeri* | *Parahucho perryi* | ***Ascelichthys*** |
| *Isopsetta isolepis* | ***Austroglossus*** | ***Salmo*** | *Ascelichthys rhodorus* |
| ***Lepidopsetta*** | *Austroglossus microlepis* | *Salmo salar* | ***Chitonotus*** |
| *Lepidopsetta bilineata* | *Austroglossus pectoralis* | *Salmo trutta* | *Chitonotus pugetensis* |
| ***Limanda*** | ***Bathysolea*** | ***Salvelinus*** | ***Clinocottus*** |
| *Limanda aspera* | *Bathysolea profundicola* | *Salvelinus alpinus* | *Clinocottus acuticeps* |
| *Limanda ferruginea* | ***Brachirus*** | *Salvelinus fontinalis* | *Clinocottus embryum* |
| *Limanda limanda* | *Brachirus orientalis* | *Salvelinus leucomaenis* | *Clinocottus globiceps* |
| ***Lyopsetta*** | ***Buglossidium*** | *Salvelinus malma* | ***Enophrys*** |
| *Lyopsetta exilis* | *Buglossidium luteum* | **Scorpaeniformes** | *Enophrys bison* |
| ***Marleyella*** | ***Dexillus*** | **Agonidae** | *Enophrys taurina* |
| *Marleyella bicolorata* | *Dexillus muelleri* | ***Agonomalus*** | ***Gymnocanthus*** |
| ***Microstomus*** | ***Dicologlossa*** | *Agonomalus mozinoi* | *Gymnocanthus tricuspis* |
| *Microstomus kitt* | *Dicologlossa cuneata* | ***Agonopsis*** | ***Hemilepidotus*** |
| *Microstomus pacificus* | *Dicologlossa hexophthalma* | *Agonopsis chiloensis* | *Hemilepidotus gilberti* |

**Table S1 (continued).**

| *Hemilepidotus hemilepidotus* | ***Hemitripterus*** | *Normanichthys crockeri* | *Parascorpaena mcadamsi* |
| --- | --- | --- | --- |
| *Hemilepidotus jordani* | *Hemitripterus americanus* | **Parabembridae** | ***Phenacoscorpius*** |
| *Hemilepidotus spinosus* | *Hemitripterus villosus* | ***Parabembras*** | *Phenacoscorpius megalops* |
| *Hemilepidotus zapus* | ***Nautichthys*** | *Parabembras curtus* | ***Pontinus*** |
| ***Icelinus*** | *Nautichthys oculofasciatus* | **Pataecidae** | *Pontinus castor* |
| *Icelinus cavifrons* | **Hexagrammidae** | ***Pataecus*** | *Pontinus furcirhinus* |
| *Icelinus tenuis* | ***Hexagrammos*** | *Pataecus fronto* | *Pontinus kuhlii* |
| ***Icelus*** | *Hexagrammos decagrammus* | **Peristediidae** | *Pontinus leda* |
| *Icelus bicornis* | *Hexagrammos lagocephalus* | ***Gargariscus*** | *Pontinus longispinis* |
| *Icelus spatula* | *Hexagrammos octogrammus* | *Gargariscus prionocephalus* | *Pontinus nematophthalmus* |
| *Icelus stenosomus* | *Hexagrammos otakii* | ***Peristedion*** | *Pontinus rathbuni* |
| ***Jordania*** | *Hexagrammos stelleri* | *Peristedion cataphractum* | ***Pterois*** |
| *Jordania zonope* | ***Ophiodon*** | *Peristedion gracile* | *Pterois antennata* |
| ***Leptocottus*** | *Ophiodon elongatus* | *Peristedion miniatum* | *Pterois miles* |
| *Leptocottus armatus* | ***Oxylebius*** | *Peristedion thompsoni* | *Pterois radiata* |
| ***Micrenophrys*** | *Oxylebius pictus* | **Platycephalidae** | *Pterois russelii* |
| *Micrenophrys lilljeborgii* | ***Pleurogrammus*** | ***Ambiserrula*** | *Pterois volitans* |
| ***Myoxocephalus*** | *Pleurogrammus azonus* | *Ambiserrula jugosa* | ***Rhinopias*** |
| *Myoxocephalus aenaeus* | *Pleurogrammus monopterygius* | ***Cymbacephalus*** | *Rhinopias frondosa* |
| *Myoxocephalus octodecemspinosus* | ***Zaniolepis*** | *Cymbacephalus beauforti* | ***Scorpaena*** |
| *Myoxocephalus polyacanthocephalus* | *Zaniolepis latipinnis* | ***Onigocia*** | *Scorpaena agassizii* |
| *Myoxocephalus quadricornis* | **Liparidae** | *Onigocia oligolepis* | *Scorpaena angolensis* |
| *Myoxocephalus scorpioides* | ***Acantholiparis*** | ***Platycephalus*** | *Scorpaena bergii* |
| *Myoxocephalus scorpius* | *Acantholiparis opercularis* | *Platycephalus bassensis* | *Scorpaena brasiliensis* |
| ***Oligocottus*** | ***Careproctus*** | *Platycephalus conatus* | *Scorpaena calcarata* |
| *Oligocottus maculosus* | *Careproctus comus* | *Platycephalus fuscus* | *Scorpaena dispar* |
| *Oligocottus rubellio* | *Careproctus faunus* | *Platycephalus grandispinis* | *Scorpaena elachys* |
| *Oligocottus snyderi* | *Careproctus ranula* | *Platycephalus indicus* | *Scorpaena elongata* |
| ***Paricelinus*** | *Careproctus reinhardti* | *Platycephalus laevigatus* | *Scorpaena grandicornis* |
| *Paricelinus hopliticus* | *Careproctus simus* | *Platycephalus marmoratus* | *Scorpaena guttata* |
| ***Radulinus*** | ***Eutelichthys*** | *Platycephalus richardsoni* | *Scorpaena histrio* |
| *Radulinus asprellus* | *Eutelichthys leptochirus* | ***Ratabulus*** | *Scorpaena inermis* |
| *Radulinus boleoides* | ***Genioliparis*** | *Ratabulus diversidens* | *Scorpaena isthmensis* |
| ***Ruscarius*** | *Genioliparis lindbergi* | ***Rogadius*** | *Scorpaena loppei* |
| *Ruscarius creaseri* | ***Liparis*** | *Rogadius welanderi* | *Scorpaena maderensis* |
| *Ruscarius meanyi* | *Liparis atlanticus* | ***Solitas*** | *Scorpaena mystes* |
| ***Scorpaenichthys*** | *Liparis coheni* | *Solitas gruveli* | *Scorpaena notata* |
| *Scorpaenichthys marmoratus* | *Liparis fabricii* | ***Sunagocia*** | *Scorpaena plumieri* |
| ***Synchirus*** | *Liparis gibbus* | *Sunagocia otaitensis* | *Scorpaena porcus* |
| *Synchirus gilli* | *Liparis inquilinus* | ***Thysanophrys*** | *Scorpaena russula* |
| ***Taurulus*** | *Liparis liparis* | *Thysanophrys chiltonae* | *Scorpaena scrofa* |
| *Taurulus bubalis* | *Liparis montagui* | *Thysanophrys cirronasa* | *Scorpaena sonorae* |
| ***Triglops*** | *Liparis pulchellus* | **Psychrolutidae** | ***Scorpaenodes*** |
| *Triglops jordani* | *Liparis tanakae* | ***Cottunculus*** | *Scorpaenodes caribbaeus* |
| *Triglops macellus* | *Liparis tunicatus* | *Cottunculus granulosus* | *Scorpaenodes englerti* |
| *Triglops metopias* | ***Nectoliparis*** | *Cottunculus microps* | *Scorpaenodes guamensis* |
| *Triglops murrayi* | *Nectoliparis pelagicus* | *Cottunculus sadko* | *Scorpaenodes hirsutus* |
| *Triglops nybelini* | ***Paraliparis*** | ***Dasycottus*** | *Scorpaenodes kelloggi* |
| *Triglops pingelii* | *Paraliparis antarcticus* | *Dasycottus setiger* | *Scorpaenodes minor* |
| *Triglops scepticus* | *Paraliparis australis* | ***Malacocottus*** | *Scorpaenodes parvipinnis* |
| *Triglops xenostethus* | *Paraliparis gracilis* | *Malacocottus kincaidi* | *Scorpaenodes xyris* |
| ***Zesticelus*** | *Paraliparis leobergi* | *Malacocottus zonurus* | ***Scorpaenopsis*** |
| *Zesticelus profundorum* | *Paraliparis meganchus* | ***Psychrolutes*** | *Scorpaenopsis cacopsis* |
| **Cyclopteridae** | *Paraliparis monoporus* | *Psychrolutes marcidus* | *Scorpaenopsis diabolus* |
| ***Aptocyclus*** | *Paraliparis operculosus* | *Psychrolutes marmoratus* | *Scorpaenopsis furneauxi* |
| *Aptocyclus ventricosus* | *Paraliparis penicillus* | *Psychrolutes paradoxus* | *Scorpaenopsis macrochir* |
| ***Cyclopterus*** | *Paraliparis thalassobathyalis* | *Psychrolutes sigalutes* | *Scorpaenopsis oxycephala* |
| *Cyclopterus lumpus* | ***Rhinoliparis*** | **Rhamphocottidae** | ***Sebastapistes*** |
| ***Eumicrotremus*** | *Rhinoliparis barbulifer* | ***Rhamphocottus*** | *Sebastapistes cyanostigma* |
| *Eumicrotremus derjugini* | ***Temnocora*** | *Rhamphocottus richardsonii* | *Sebastapistes fowleri* |
| *Eumicrotremus orbis* | *Temnocora candida* | **Scorpaenidae** | *Sebastapistes mauritiana* |
| *Eumicrotremus spinosus* | **Neosebastidae** | ***Caracanthus*** | *Sebastapistes strongia* |
| **Dactylopteridae** | ***Maxillicosta*** | *Caracanthus maculatus* | ***Taenianotus*** |
| ***Dactyloptena*** | *Maxillicosta scabriceps* | *Caracanthus unipinna* | *Taenianotus triacanthus* |
| *Dactyloptena orientalis* | *Maxillicosta whitleyi* | ***Dendrochirus*** | **Sebastidae** |
| ***Dactylopterus*** | ***Neosebastes*** | *Dendrochirus bellus* | ***Helicolenus*** |
| *Dactylopterus volitans* | *Neosebastes bougainvillii* | *Dendrochirus biocellatus* | *Helicolenus dactylopterus* |
| **Hemitripteridae** | *Neosebastes incisipinnis* | *Dendrochirus brachypterus* | *Helicolenus lengerichi* |
| ***Blepsias*** | *Neosebastes nigropunctatus* | *Dendrochirus zebra* | *Helicolenus percoides* |
| *Blepsias bilobus* | *Neosebastes pandus* | ***Neomerinthe*** | ***Sebastes*** |
| *Blepsias cirrhosus* | **Normanichthyidae** | *Neomerinthe hemingwayi* | *Sebastes aleutianus* |
|  | ***Normanichthys*** | ***Parascorpaena*** | *Sebastes alutus* |

**Table S1 (continued).**

| *Sebastes atrovirens* | ***Lioscorpius*** | *Prionotus stephanophrys* | *Scopelogadus beanii* |
| --- | --- | --- | --- |
| *Sebastes auriculatus* | *Lioscorpius trifasciatus* | *Prionotus teaguei* | *Scopelogadus bispinosus* |
| *Sebastes aurora* | ***Setarches*** | *Prionotus tribulus* | *Scopelogadus mizolepis* |
| *Sebastes babcocki* | *Setarches guentheri* | ***Pterygotrigla*** | ***Sio*** |
| *Sebastes borealis* | **Synanceiidae** | *Pterygotrigla polyommata* | *Sio nordenskjoldii* |
| *Sebastes brevispinis* | ***Inimicus*** | ***Trigla*** | **Stephanoberycidae** |
| *Sebastes capensis* | *Inimicus didactylus* | *Trigla lyra* | ***Acanthochaenus*** |
| *Sebastes carnatus* | ***Minous*** | **Siluriformes** | *Acanthochaenus luetkenii* |
| *Sebastes caurinus* | *Minous versicolor* | **Ariidae** | **Stomiiformes** |
| *Sebastes chlorostictus* | ***Synanceia*** | ***Amphiarius*** | **Gonostomatidae** |
| *Sebastes chrysomelas* | *Synanceia nana* | *Amphiarius phrygiatus* | ***Bonapartia*** |
| *Sebastes ciliatus* | *Synanceia verrucosa* | *Amphiarius rugispinis* | *Bonapartia pedaliota* |
| *Sebastes constellatus* | **Tetrarogidae** | ***Ariopsis*** | ***Cyclothone*** |
| *Sebastes cortezi* | ***Ablabys*** | *Ariopsis felis* | *Cyclothone acclinidens* |
| *Sebastes crameri* | *Ablabys macracanthus* | *Ariopsis guatemalensis* | *Cyclothone alba* |
| *Sebastes dallii* | *Ablabys taenianotus* | ***Arius*** | *Cyclothone atraria* |
| *Sebastes diploproa* | ***Centropogon*** | *Arius maculatus* | *Cyclothone braueri* |
| *Sebastes elongatus* | *Centropogon australis* | ***Aspistor*** | *Cyclothone kobayashii* |
| *Sebastes emphaeus* | ***Glyptauchen*** | *Aspistor quadriscutis* | *Cyclothone livida* |
| *Sebastes ensifer* | *Glyptauchen panduratus* | ***Bagre*** | *Cyclothone microdon* |
| *Sebastes entomelas* | ***Gymnapistes*** | *Bagre bagre* | *Cyclothone obscura* |
| *Sebastes eos* | *Gymnapistes marmoratus* | *Bagre marinus* | *Cyclothone pallida* |
| *Sebastes fasciatus* | ***Liocranium*** | *Bagre pinnimaculatus* | *Cyclothone pseudopallida* |
| *Sebastes flavidus* | *Liocranium praepositum* | ***Carlarius*** | *Cyclothone pygmaea* |
| *Sebastes gilli* | ***Neovespicula*** | *Carlarius heudelotii* | ***Diplophos*** |
| *Sebastes goodei* | *Neovespicula depressifrons* | ***Cathorops*** | *Diplophos taenia* |
| *Sebastes helvomaculatus* | ***Tetraroge*** | *Cathorops spixii* | ***Gonostoma*** |
| *Sebastes hopkinsi* | *Tetraroge barbata* | ***Galeichthys*** | *Gonostoma atlanticum* |
| *Sebastes inermis* | **Triglidae** | *Galeichthys feliceps* | *Gonostoma denudatum* |
| *Sebastes jordani* | ***Bellator*** | ***Nemapteryx*** | ***Manducus*** |
| *Sebastes lentiginosus* | *Bellator brachychir* | *Nemapteryx caelata* | *Manducus maderensis* |
| *Sebastes levis* | *Bellator egretta* | ***Neoarius*** | ***Margrethia*** |
| *Sebastes macdonaldi* | *Bellator gymnostethus* | *Neoarius graeffei* | *Margrethia obtusirostra* |
| *Sebastes maliger* | *Bellator loxias* | *Neoarius leptaspis* | ***Sigmops*** |
| *Sebastes melanops* | *Bellator militaris* | ***Netuma*** | *Sigmops bathyphilus* |
| *Sebastes melanostomus* | *Bellator ribeiroi* | *Netuma thalassina* | *Sigmops elongatus* |
| *Sebastes mentella* | *Bellator xenisma* | ***Notarius*** | *Sigmops gracilis* |
| *Sebastes miniatus* | ***Chelidonichthys*** | *Notarius grandicassis* | **Phosichthyidae** |
| *Sebastes mystinus* | *Chelidonichthys capensis* | *Notarius troschelii* | ***Ichthyococcus*** |
| *Sebastes nebulosus* | *Chelidonichthys cuculus* | ***Occidentarius*** | *Ichthyococcus elongatus* |
| *Sebastes nigrocinctus* | *Chelidonichthys kumu* | *Occidentarius platypogon* | *Ichthyococcus intermedius* |
| *Sebastes norvegicus* | *Chelidonichthys lucerna* | ***Sciades*** | *Ichthyococcus irregularis* |
| *Sebastes ovalis* | *Chelidonichthys obscurus* | *Sciades parkeri* | *Ichthyococcus ovatus* |
| *Sebastes paucispinis* | *Chelidonichthys queketti* | *Sciades proops* | *Ichthyococcus polli* |
| *Sebastes pinniger* | ***Eutrigla*** | **Plotosidae** | ***Phosichthys*** |
| *Sebastes proriger* | *Eutrigla gurnardus* | ***Cnidoglanis*** | *Phosichthys argenteus* |
| *Sebastes rastrelliger* | ***Lepidotrigla*** | *Cnidoglanis macrocephalus* | ***Pollichthys*** |
| *Sebastes reedi* | *Lepidotrigla brachyoptera* | ***Plotosus*** | *Pollichthys mauli* |
| *Sebastes rosaceus* | *Lepidotrigla cadmani* | *Plotosus lineatus* | ***Polymetme*** |
| *Sebastes rosenblatti* | *Lepidotrigla calodactyla* | **Stephanoberyciformes** | *Polymetme corythaeola* |
| *Sebastes ruberrimus* | *Lepidotrigla carolae* | **Gibberichthyidae** | ***Vinciguerria*** |
| *Sebastes rubrivinctus* | *Lepidotrigla cavillone* | ***Gibberichthys*** | *Vinciguerria attenuata* |
| *Sebastes rufus* | *Lepidotrigla dieuzeidei* | *Gibberichthys pumilus* | *Vinciguerria nimbaria* |
| *Sebastes saxicola* | *Lepidotrigla modesta* | **Melamphaidae** | *Vinciguerria poweriae* |
| *Sebastes semicinctus* | *Lepidotrigla mulhalli* | ***Melamphaes*** | ***Woodsia*** |
| *Sebastes serranoides* | *Lepidotrigla papilio* | *Melamphaes danae* | *Woodsia nonsuchae* |
| *Sebastes serriceps* | *Lepidotrigla vanessa* | *Melamphaes hubbsi* | ***Yarrella*** |
| *Sebastes simulator* | ***Prionotus*** | *Melamphaes leprus* | *Yarrella blackfordi* |
| *Sebastes sinensis* | *Prionotus alatus* | *Melamphaes lugubris* | **Sternoptychidae** |
| *Sebastes umbrosus* | *Prionotus albirostris* | *Melamphaes macrocephalus* | ***Argyropelecus*** |
| *Sebastes variegatus* | *Prionotus birostratus* | *Melamphaes microps* | *Argyropelecus aculeatus* |
| *Sebastes viviparus* | *Prionotus carolinus* | *Melamphaes polylepis* | *Argyropelecus affinis* |
| *Sebastes zacentrus* | *Prionotus evolans* | *Melamphaes pumilus* | *Argyropelecus gigas* |
| ***Sebastolobus*** | *Prionotus longispinosus* | *Melamphaes suborbitalis* | *Argyropelecus hemigymnus* |
| *Sebastolobus alascanus* | *Prionotus ophryas* | *Melamphaes typhlops* | *Argyropelecus olfersii* |
| *Sebastolobus altivelis* | *Prionotus paralatus* | ***Poromitra*** | *Argyropelecus sladeni* |
| *Sebastolobus macrochir* | *Prionotus punctatus* | *Poromitra capito* | ***Danaphos*** |
| ***Trachyscorpia*** | *Prionotus roseus* | *Poromitra crassa* | *Danaphos oculatus* |
| *Trachyscorpia cristulata cristulata* | *Prionotus rubio* | *Poromitra oscitans* | ***Maurolicus*** |
| *Trachyscorpia cristulata echinata* | *Prionotus ruscarius* | ***Scopeloberyx*** | *Maurolicus muelleri* |
| *Trachyscorpia eschmeyeri* | *Prionotus scitulus* | *Scopeloberyx microlepis* | *Maurolicus stehmanni* |
| **Setarchidae** | *Prionotus stearnsi* | ***Scopelogadus*** | *Maurolicus walvisensis* |

**Table S1 (continued).**

| ***Polyipnus*** | *Eustomias melanonema* | ***Thysanactis*** | *Hippocampus breviceps* |
| --- | --- | --- | --- |
| *Polyipnus polli* | *Eustomias melanostigma* | *Thysanactis dentex* | *Hippocampus camelopardalis* |
| ***Sternoptyx*** | *Eustomias monoclonus* | ***Trigonolampa*** | *Hippocampus comes* |
| *Sternoptyx diaphana* | *Eustomias obscurus* | *Trigonolampa miriceps* | *Hippocampus erectus* |
| *Sternoptyx pseudobscura* | *Eustomias patulus* | **Syngnathiformes** | *Hippocampus guttulatus* |
| ***Valenciennellus*** | *Eustomias satterleei* | **Aulostomidae** | *Hippocampus hippocampus* |
| *Valenciennellus tripunctulatus* | *Eustomias schmidti* | ***Aulostomus*** | *Hippocampus histrix* |
| **Stomiidae** | *Eustomias simplex* | *Aulostomus chinensis* | *Hippocampus kuda* |
| ***Aristostomias*** | *Eustomias spherulifer* | *Aulostomus maculatus* | *Hippocampus reidi* |
| *Aristostomias grimaldii* | *Eustomias tenisoni* | **Centriscidae** | *Hippocampus spinosissimus* |
| *Aristostomias lunifer* | *Eustomias tetranema* | ***Aeoliscus*** | *Hippocampus trimaculatus* |
| *Aristostomias polydactylus* | *Eustomias trewavasae* | *Aeoliscus strigatus* | *Hippocampus whitei* |
| *Aristostomias scintillans* | ***Flagellostomias*** | ***Macroramphosus*** | *Hippocampus zebra* |
| *Aristostomias tittmanni* | *Flagellostomias boureei* | *Macroramphosus gracilis* | *Hippocampus zosterae* |
| *Aristostomias xenostoma* | ***Grammatostomias*** | *Macroramphosus scolopax* | ***Ichthyocampus*** |
| ***Astronesthes*** | *Grammatostomias circularis* | **Fistulariidae** | *Ichthyocampus carce* |
| *Astronesthes bilobatus* | ***Heterophotus*** | ***Fistularia*** | ***Micrognathus*** |
| *Astronesthes boulengeri* | *Heterophotus ophistoma* | *Fistularia commersonii* | *Micrognathus andersonii* |
| *Astronesthes caulophorus* | ***Idiacanthus*** | *Fistularia corneta* | *Micrognathus crinitus* |
| *Astronesthes cyaneus* | *Idiacanthus antrostomus* | *Fistularia petimba* | *Micrognathus micronotopterus* |
| *Astronesthes gemmifer* | *Idiacanthus atlanticus* | *Fistularia tabacaria* | ***Microphis*** |
| *Astronesthes illuminatus* | *Idiacanthus fasciola* | **Syngnathidae** | *Microphis argulus* |
| *Astronesthes indopacificus* | ***Leptostomias*** | ***Amphelikturus*** | *Microphis brachyurus* |
| *Astronesthes leucopogon* | *Leptostomias gladiator* | *Amphelikturus dendriticus* | ***Minyichthys*** |
| *Astronesthes lucifer* | *Leptostomias gracilis* | ***Anarchopterus*** | *Minyichthys myersi* |
| *Astronesthes macropogon* | *Leptostomias haplocaulus* | *Anarchopterus tectus* | ***Nannocampus*** |
| *Astronesthes micropogon* | *Leptostomias longibarba* | ***Bhanotia*** | *Nannocampus elegans* |
| *Astronesthes neopogon* | ***Malacosteus*** | *Bhanotia nuda* | ***Nerophis*** |
| *Astronesthes niger* | *Malacosteus australis* | ***Bryx*** | *Nerophis lumbriciformis* |
| *Astronesthes richardsoni* | *Malacosteus niger* | *Bryx dunckeri* | *Nerophis ophidion* |
| *Astronesthes similus* | ***Melanostomias*** | ***Bulbonaricus*** | ***Phoxocampus*** |
| *Astronesthes splendidus* | *Melanostomias bartonbeani* | *Bulbonaricus brauni* | *Phoxocampus diacanthus* |
| ***Bathophilus*** | *Melanostomias biseriatus* | ***Choeroichthys*** | ***Phycodurus*** |
| *Bathophilus ater* | *Melanostomias macrophotus* | *Choeroichthys brachysoma* | *Phycodurus eques* |
| *Bathophilus brevis* | *Melanostomias melanopogon* | *Choeroichthys sculptus* | ***Pugnaso*** |
| *Bathophilus digitatus* | *Melanostomias melanops* | ***Corythoichthys*** | *Pugnaso curtirostris* |
| *Bathophilus flemingi* | *Melanostomias niger* | *Corythoichthys amplexus* | ***Stigmatopora*** |
| *Bathophilus longipinnis* | *Melanostomias tentaculatus* | *Corythoichthys flavofasciatus* | *Stigmatopora nigra* |
| *Bathophilus nigerrimus* | *Melanostomias valdiviae* | *Corythoichthys haematopterus* | ***Syngnathoides*** |
| *Bathophilus pawneei* | ***Neonesthes*** | *Corythoichthys intestinalis* | *Syngnathoides biaculeatus* |
| *Bathophilus schizochirus* | *Neonesthes capensis* | *Corythoichthys nigripectus* | ***Syngnathus*** |
| *Bathophilus vaillanti* | ***Odontostomias*** | *Corythoichthys ocellatus* | *Syngnathus abaster* |
| ***Borostomias*** | *Odontostomias micropogon* | *Corythoichthys schultzi* | *Syngnathus acus* |
| *Borostomias antarcticus* | ***Opostomias*** | ***Cosmocampus*** | *Syngnathus floridae* |
| *Borostomias elucens* | *Opostomias mitsuii* | *Cosmocampus albirostris* | *Syngnathus fuscus* |
| ***Chauliodus*** | ***Pachystomias*** | *Cosmocampus banneri* | *Syngnathus leptorhynchus* |
| *Chauliodus danae* | *Pachystomias microdon* | *Cosmocampus darrosanus* | *Syngnathus louisianae* |
| *Chauliodus macouni* | ***Photonectes*** | *Cosmocampus elucens* | *Syngnathus rostellatus* |
| *Chauliodus minimus* | *Photonectes albipennis* | *Cosmocampus maxweberi* | *Syngnathus springeri* |
| *Chauliodus pammelas* | *Photonectes braueri* | ***Doryrhamphus*** | ***Trachyrhamphus*** |
| *Chauliodus schmidti* | *Photonectes caerulescens* | *Doryrhamphus excisus excisus* | *Trachyrhamphus bicoarctatus* |
| *Chauliodus sloani* | *Photonectes dinema* | *Doryrhamphus janssi* | ***Urocampus*** |
| ***Chirostomias*** | *Photonectes leucospilus* | *Doryrhamphus negrosensis* | *Urocampus carinirostris* |
| *Chirostomias pliopterus* | *Photonectes margarita* | ***Dunckerocampus*** | **Tetraodontiformes** |
| ***Echiostoma*** | *Photonectes mirabilis* | *Dunckerocampus dactyliophorus* | **Balistidae** |
| *Echiostoma barbatum* | *Photonectes parvimanus* | ***Entelurus*** | ***Abalistes*** |
| ***Eustomias*** | *Photonectes phyllopogon* | *Entelurus aequoreus* | *Abalistes stellaris* |
| *Eustomias achirus* | ***Photostomias*** | ***Halicampus*** | *Abalistes stellatus* |
| *Eustomias arborifer* | *Photostomias guernei* | *Halicampus brocki* | ***Balistapus*** |
| *Eustomias bibulbosus* | ***Rhadinesthes*** | *Halicampus dunckeri* | *Balistapus undulatus* |
| *Eustomias bigelowi* | *Rhadinesthes decimus* | *Halicampus nitidus* | ***Balistes*** |
| *Eustomias bimargaritatus* | ***Stomias*** | ***Haliichthys*** | *Balistes capriscus* |
| *Eustomias braueri* | *Stomias affinis* | *Haliichthys taeniophorus* | *Balistes polylepis* |
| *Eustomias bulbornatus* | *Stomias atriventer* | ***Hippichthys*** | *Balistes punctatus* |
| *Eustomias dendriticus* | *Stomias boa boa* | *Hippichthys penicillus* | *Balistes vetula* |
| *Eustomias enbarbatus* | *Stomias boa ferox* | *Hippichthys spicifer* | ***Balistoides*** |
| *Eustomias filifer* | *Stomias brevibarbatus* | ***Hippocampus*** | *Balistoides conspicillum* |
| *Eustomias fissibarbis* | *Stomias gracilis* | *Hippocampus abdominalis* | *Balistoides viridescens* |
| *Eustomias kreffti* | *Stomias lampropeltis* | *Hippocampus algiricus* | ***Canthidermis*** |
| *Eustomias lipochirus* | *Stomias longibarbatus* | *Hippocampus angustus* | *Canthidermis maculata* |
| *Eustomias longibarba* | ***Tactostoma*** | *Hippocampus barbouri* | *Canthidermis sufflamen* |
| *Eustomias macrurus* | *Tactostoma macropus* | *Hippocampus bargibanti* | ***Melichthys*** |

**Table S1 (continued).**

| *Melichthys niger* | ***Paramonacanthus*** | *Sphoeroides lobatus* | *Carcharhinus altimus* |
| --- | --- | --- | --- |
| *Melichthys vidua* | *Paramonacanthus cryptodon* | *Sphoeroides maculatus* | *Carcharhinus amblyrhynchoides* |
| ***Odonus*** | *Paramonacanthus japonicus* | *Sphoeroides marmoratus* | *Carcharhinus amblyrhynchos* |
| *Odonus niger* | *Paramonacanthus sulcatus* | *Sphoeroides nephelus* | *Carcharhinus amboinensis* |
| ***Pseudobalistes*** | ***Pervagor*** | *Sphoeroides pachygaster* | *Carcharhinus borneensis* |
| *Pseudobalistes flavimarginatus* | *Pervagor aspricaudus* | *Sphoeroides parvus* | *Carcharhinus brachyurus* |
| *Pseudobalistes fuscus* | *Pervagor janthinosoma* | *Sphoeroides sechurae* | *Carcharhinus brevipinna* |
| *Pseudobalistes naufragium* | *Pervagor melanocephalus* | *Sphoeroides spengleri* | *Carcharhinus cautus* |
| ***Rhinecanthus*** | *Pervagor nigrolineatus* | *Sphoeroides testudineus* | *Carcharhinus dussumieri* |
| *Rhinecanthus aculeatus* | *Pervagor randalli* | ***Takifugu*** | *Carcharhinus falciformis* |
| *Rhinecanthus rectangulus* | *Pervagor spilosoma* | *Takifugu porphyreus* | *Carcharhinus fitzroyensis* |
| *Rhinecanthus verrucosus* | ***Rudarius*** | *Takifugu rubripes* | *Carcharhinus galapagensis* |
| ***Sufflamen*** | *Rudarius minutus* | *Takifugu vermicularis* | *Carcharhinus hemiodon* |
| *Sufflamen bursa* | ***Stephanolepis*** | ***Torquigener*** | *Carcharhinus isodon* |
| *Sufflamen chrysopterum* | *Stephanolepis cirrhifer* | *Torquigener perlevis* | *Carcharhinus leucas* |
| *Sufflamen fraenatum* | *Stephanolepis diaspros* | **Triacanthidae** | *Carcharhinus limbatus* |
| *Sufflamen verres* | *Stephanolepis hispidus* | ***Triacanthus*** | *Carcharhinus longimanus* |
| ***Xanthichthys*** | *Stephanolepis setifer* | *Triacanthus biaculeatus* | *Carcharhinus macloti* |
| *Xanthichthys auromarginatus* | **Ostraciidae** | *Triacanthus nieuhofii* | *Carcharhinus melanopterus* |
| *Xanthichthys caeruleolineatus* | ***Acanthostracion*** | **Triacanthodidae** | *Carcharhinus obscurus* |
| *Xanthichthys mento* | *Acanthostracion guineensis* | ***Hollardia*** | *Carcharhinus perezi* |
| *Xanthichthys ringens* | *Acanthostracion polygonius* | *Hollardia hollardi* | *Carcharhinus plumbeus* |
| **Diodontidae** | *Acanthostracion quadricornis* | *Hollardia meadi* | *Carcharhinus porosus* |
| ***Chilomycterus*** | ***Lactophrys*** | ***Parahollardia*** | *Carcharhinus sealei* |
| *Chilomycterus schoepfii* | *Lactophrys bicaudalis* | *Parahollardia lineata* | *Carcharhinus signatus* |
| ***Cyclichthys*** | *Lactophrys trigonus* | **Zeiformes** | *Carcharhinus sorrah* |
| *Cyclichthys orbicularis* | *Lactophrys triqueter* | **Grammicolepididae** | *Carcharhinus tilstoni* |
| ***Diodon*** | ***Lactoria*** | ***Grammicolepis*** | ***Galeocerdo*** |
| *Diodon eydouxii* | *Lactoria cornuta* | *Grammicolepis brachiusculus* | *Galeocerdo cuvier* |
| *Diodon holocanthus* | *Lactoria diaphana* | ***Xenolepidichthys*** | ***Glyphis*** |
| *Diodon hystrix* | *Lactoria fornasini* | *Xenolepidichthys dalgleishi* | *Glyphis gangeticus* |
| *Diodon liturosus* | ***Ostracion*** | **Oreosomatidae** | ***Isogomphodon*** |
| **Molidae** | *Ostracion cubicus* | ***Allocyttus*** | *Isogomphodon oxyrhynchus* |
| ***Masturus*** | *Ostracion meleagris* | *Allocyttus guineensis* | ***Lamiopsis*** |
| *Masturus lanceolatus* | *Ostracion solorensis* | *Allocyttus niger* | *Lamiopsis temminckii* |
| ***Mola*** | ***Rhynchostracion*** | *Allocyttus verrucosus* | ***Loxodon*** |
| *Mola mola* | *Rhynchostracion nasus* | ***Neocyttus*** | *Loxodon macrorhinus* |
| ***Ranzania*** | ***Tetrosomus*** | *Neocyttus helgae* | ***Nasolamia*** |
| *Ranzania laevis* | *Tetrosomus concatenatus* | *Neocyttus rhomboidalis* | *Nasolamia velox* |
| **Monacanthidae** | **Tetraodontidae** | ***Pseudocyttus*** | ***Negaprion*** |
| ***Acanthaluteres*** | ***Arothron*** | *Pseudocyttus maculatus* | *Negaprion acutidens* |
| *Acanthaluteres brownii* | *Arothron hispidus* | **Zeidae** | *Negaprion brevirostris* |
| *Acanthaluteres spilomelanurus* | *Arothron immaculatus* | ***Zenopsis*** | ***Prionace*** |
| *Acanthaluteres vittiger* | *Arothron manilensis* | *Zenopsis conchifer* | *Prionace glauca* |
| ***Acreichthys*** | *Arothron mappa* | *Zenopsis nebulosa* | ***Rhizoprionodon*** |
| *Acreichthys tomentosus* | *Arothron meleagris* | ***Zeus*** | *Rhizoprionodon acutus* |
| ***Aluterus*** | *Arothron nigropunctatus* | *Zeus capensis* | *Rhizoprionodon lalandii* |
| *Aluterus heudelotii* | *Arothron stellatus* | *Zeus faber* | *Rhizoprionodon longurio* |
| *Aluterus monoceros* | ***Canthigaster*** | **Zeniontidae** | *Rhizoprionodon oligolinx* |
| *Aluterus schoepfii* | *Canthigaster amboinensis* | ***Zenion*** | *Rhizoprionodon porosus* |
| *Aluterus scriptus* | *Canthigaster bennetti* | *Zenion hololepis* | *Rhizoprionodon taylori* |
| ***Amanses*** | *Canthigaster compressa* | **Cephalaspidomorphi** | *Rhizoprionodon terraenovae* |
| *Amanses scopas* | *Canthigaster coronata* | **Petromyzontiformes** | ***Scoliodon*** |
| ***Brachaluteres*** | *Canthigaster epilampra* | **Mordaciidae** | *Scoliodon laticaudus* |
| *Brachaluteres taylori* | *Canthigaster figueiredoi* | ***Mordacia*** | ***Triaenodon*** |
| ***Cantherhines*** | *Canthigaster jactator* | *Mordacia mordax* | *Triaenodon obesus* |
| *Cantherhines dumerilii* | *Canthigaster janthinoptera* | **Petromyzontidae** | **Hemigaleidae** |
| *Cantherhines fronticinctus* | *Canthigaster leoparda* | ***Entosphenus*** | ***Chaenogaleus*** |
| *Cantherhines macrocerus* | *Canthigaster papua* | *Entosphenus tridentatus* | *Chaenogaleus macrostoma* |
| *Cantherhines pardalis* | *Canthigaster punctatissima* | ***Lampetra*** | ***Hemigaleus*** |
| *Cantherhines pullus* | *Canthigaster rostrata* | *Lampetra ayresii* | *Hemigaleus microstoma* |
| ***Meuschenia*** | *Canthigaster solandri* | *Lampetra fluviatilis* | ***Hemipristis*** |
| *Meuschenia scaber* | *Canthigaster valentini* | ***Lethenteron*** | *Hemipristis elongata* |
| ***Monacanthus*** | ***Chelonodon*** | *Lethenteron camtschaticum* | ***Paragaleus*** |
| *Monacanthus ciliatus* | *Chelonodon patoca* | ***Petromyzon*** | *Paragaleus pectoralis* |
| *Monacanthus tuckeri* | ***Ephippion*** | *Petromyzon marinus* | *Paragaleus tengi* |
| ***Nelusetta*** | *Ephippion guttifer* | **Elasmobranchii** | **Leptochariidae** |
| *Nelusetta ayraud* | ***Lagocephalus*** | **Carcharhiniformes** | ***Leptocharias*** |
| ***Oxymonacanthus*** | *Lagocephalus laevigatus* | **Carcharhinidae** | *Leptocharias smithii* |
| *Oxymonacanthus longirostris* | ***Sphoeroides*** | ***Carcharhinus*** | **Proscylliidae** |
| ***Paraluteres*** | *Sphoeroides annulatus* | *Carcharhinus acronotus* | ***Eridacnis*** |
| *Paraluteres prionurus* | *Sphoeroides dorsalis* | *Carcharhinus albimarginatus* | *Eridacnis radcliffei* |

**Table S1 (continued).**

| *Eridacnis sinuans* | *Schroederichthys maculatus* | *Heterodontus zebra* | *Pteroplatytrygon violacea* |
| --- | --- | --- | --- |
| ***Proscyllium*** | ***Scyliorhinus*** | **Hexanchiformes** | ***Taeniura*** |
| *Proscyllium habereri* | *Scyliorhinus besnardi* | **Chlamydoselachidae** | *Taeniura grabata* |
| **Pseudotriakidae** | *Scyliorhinus boa* | ***Chlamydoselachus*** | *Taeniura lymma* |
| ***Pseudotriakis*** | *Scyliorhinus canicula* | *Chlamydoselachus anguineus* | ***Taeniurops*** |
| *Pseudotriakis microdon* | *Scyliorhinus capensis* | **Hexanchidae** | *Taeniurops meyeni* |
| **Scyliorhinidae** | *Scyliorhinus cervigoni* | ***Heptranchias*** | ***Urogymnus*** |
| ***Apristurus*** | *Scyliorhinus garmani* | *Heptranchias perlo* | *Urogymnus granulatus* |
| *Apristurus brunneus* | *Scyliorhinus haeckelii* | ***Hexanchus*** | **Gymnuridae** |
| *Apristurus canutus* | *Scyliorhinus hesperius* | *Hexanchus griseus* | ***Gymnura*** |
| *Apristurus herklotsi* | *Scyliorhinus meadi* | *Hexanchus nakamurai* | *Gymnura altavela* |
| *Apristurus indicus* | *Scyliorhinus retifer* | ***Notorynchus*** | *Gymnura australis* |
| *Apristurus kampae* | *Scyliorhinus stellaris* | *Notorynchus cepedianus* | *Gymnura marmorata* |
| *Apristurus laurussonii* | *Scyliorhinus torrei* | **Lamniformes** | **Hexatrygonidae** |
| *Apristurus longicephalus* | **Sphyrnidae** | **Alopiidae** | ***Hexatrygon*** |
| *Apristurus macrorhynchus* | ***Eusphyra*** | ***Alopias*** | *Hexatrygon bickelli* |
| *Apristurus manis* | *Eusphyra blochii* | *Alopias pelagicus* | **Myliobatidae** |
| *Apristurus microps* | ***Sphyrna*** | *Alopias superciliosus* | ***Aetomylaeus*** |
| *Apristurus parvipinnis* | *Sphyrna corona* | *Alopias vulpinus* | *Aetomylaeus bovinus* |
| *Apristurus platyrhynchus* | *Sphyrna couardi* | **Cetorhinidae** | *Aetomylaeus maculatus* |
| *Apristurus profundorum* | *Sphyrna lewini* | ***Cetorhinus*** | *Aetomylaeus nichofii* |
| *Apristurus riveri* | *Sphyrna mokarran* | *Cetorhinus maximus* | *Aetomylaeus vespertilio* |
| *Apristurus saldanha* | *Sphyrna tiburo* | **Lamnidae** | ***Mobula*** |
| ***Asymbolus*** | *Sphyrna tudes* | ***Carcharodon*** | *Mobula alfredi* |
| *Asymbolus analis* | *Sphyrna zygaena* | *Carcharodon carcharias* | *Mobula birostris* |
| *Asymbolus vincenti* | **Triakidae** | ***Isurus*** | *Mobula eregoodootenkee* |
| ***Atelomycterus*** | ***Furgaleus*** | *Isurus oxyrinchus* | *Mobula hypostoma* |
| *Atelomycterus fasciatus* | *Furgaleus macki* | *Isurus paucus* | *Mobula japanica* |
| *Atelomycterus macleayi* | ***Galeorhinus*** | ***Lamna*** | *Mobula mobular* |
| ***Bythaelurus*** | *Galeorhinus galeus* | *Lamna ditropis* | *Mobula thurstoni* |
| *Bythaelurus canescens* | ***Hemitriakis*** | *Lamna nasus* | ***Myliobatis*** |
| *Bythaelurus hispidus* | *Hemitriakis japanica* | **Megachasmidae** | *Myliobatis aquila* |
| *Bythaelurus lutarius* | *Hemitriakis leucoperiptera* | ***Megachasma*** | *Myliobatis australis* |
| ***Cephaloscyllium*** | ***Hypogaleus*** | *Megachasma pelagios* | *Myliobatis californica* |
| *Cephaloscyllium fasciatum* | *Hypogaleus hyugaensis* | **Mitsukurinidae** | *Myliobatis freminvillei* |
| *Cephaloscyllium isabellum* | ***Iago*** | ***Mitsukurina*** | *Myliobatis goodei* |
| *Cephaloscyllium laticeps* | *Iago garricki* | *Mitsukurina owstoni* | *Myliobatis peruvianus* |
| *Cephaloscyllium sufflans* | *Iago omanensis* | **Odontaspididae** | ***Rhinoptera*** |
| *Cephaloscyllium ventriosum* | ***Mustelus*** | ***Carcharias*** | *Rhinoptera bonasus* |
| ***Cephalurus*** | *Mustelus antarcticus* | *Carcharias taurus* | *Rhinoptera marginata* |
| *Cephalurus cephalus* | *Mustelus asterias* | ***Odontaspis*** | **Plesiobatidae** |
| ***Figaro*** | *Mustelus californicus* | *Odontaspis ferox* | ***Plesiobatis*** |
| *Figaro boardmani* | *Mustelus canis* | *Odontaspis noronhai* | *Plesiobatis daviesi* |
| ***Galeus*** | *Mustelus dorsalis* | **Pseudocarchariidae** | **Urolophidae** |
| *Galeus arae* | *Mustelus fasciatus* | ***Pseudocarcharias*** | ***Trygonoptera*** |
| *Galeus eastmani* | *Mustelus griseus* | *Pseudocarcharias kamoharai* | *Trygonoptera ovalis* |
| *Galeus gracilis* | *Mustelus henlei* | **Myliobatiformes** | *Trygonoptera testacea* |
| *Galeus melastomus* | *Mustelus higmani* | **Aetobatidae** | ***Urolophus*** |
| *Galeus murinus* | *Mustelus lenticulatus* | ***Aetobatus*** | *Urolophus bucculentus* |
| *Galeus nipponensis* | *Mustelus lunulatus* | *Aetobatus narinari* | *Urolophus cruciatus* |
| *Galeus piperatus* | *Mustelus manazo* | **Dasyatidae** | *Urolophus expansus* |
| *Galeus polli* | *Mustelus mento* | ***Dasyatis*** | *Urolophus flavomosaicus* |
| *Galeus sauteri* | *Mustelus mustelus* | *Dasyatis brevis* | *Urolophus halleri* |
| ***Halaelurus*** | *Mustelus norrisi* | *Dasyatis hypostigma* | *Urolophus neocaledoniensis* |
| *Halaelurus boesemani* | *Mustelus palumbes* | *Dasyatis pastinaca* | *Urolophus paucimaculatus* |
| *Halaelurus buergeri* | *Mustelus punctulatus* | *Dasyatis thetidis* | *Urolophus sufflavus* |
| *Halaelurus lineatus* | *Mustelus schmitti* | *Dasyatis tortonesei* | *Urolophus viridis* |
| *Halaelurus natalensis* | *Mustelus whitneyi* | ***Himantura*** | *Urolophus westraliensis* |
| ***Haploblepharus*** | ***Triakis*** | *Himantura uarnak* | **Urotrygonidae** |
| *Haploblepharus edwardsii* | *Triakis megalopterus* | *Himantura undulata* | ***Urobatis*** |
| *Haploblepharus fuscus* | *Triakis scyllium* | ***Hypanus*** | *Urobatis concentricus* |
| ***Holohalaelurus*** | *Triakis semifasciata* | *Hypanus americanus* | *Urobatis jamaicensis* |
| *Holohalaelurus punctatus* | **Heterodontiformes** | *Hypanus longus* | ***Urotrygon*** |
| *Holohalaelurus regani* | **Heterodontidae** | ***Neotrygon*** | *Urotrygon chilensis* |
| ***Parmaturus*** | ***Heterodontus*** | *Neotrygon annotata* | *Urotrygon munda* |
| *Parmaturus xaniurus* | *Heterodontus francisci* | *Neotrygon kuhlii* | *Urotrygon nana* |
| ***Poroderma*** | *Heterodontus galeatus* | *Neotrygon leylandi* | *Urotrygon rogersi* |
| *Poroderma africanum* | *Heterodontus japonicus* | ***Pastinachus*** | **Orectolobiformes** |
| *Poroderma pantherinum* | *Heterodontus mexicanus* | *Pastinachus sephen* | **Brachaeluridae** |
| ***Schroederichthys*** | *Heterodontus portusjacksoni* | ***Pateobatis*** | ***Brachaelurus*** |
| *Schroederichthys bivius* | *Heterodontus quoyi* | *Pateobatis fai* | *Brachaelurus colcloughi* |
| *Schroederichthys chilensis* | *Heterodontus ramalheira* | ***Pteroplatytrygon*** | *Brachaelurus waddi* |

**Table S1 (continued).**

| **Ginglymostomatidae** | *Bathyraja meridionalis* | *Raja equatorialis* | ***Deania*** |
| --- | --- | --- | --- |
| ***Ginglymostoma*** | *Bathyraja murrayi* | *Raja herwigi* | *Deania calcea* |
| *Ginglymostoma cirratum* | *Bathyraja richardsoni* | *Raja microocellata* | *Deania hystricosa* |
| ***Nebrius*** | *Bathyraja scaphiops* | *Raja miraletus* | *Deania profundorum* |
| *Nebrius ferrugineus* | *Bathyraja spinicauda* | *Raja montagui* | *Deania quadrispinosa* |
| **Hemiscylliidae** | *Bathyraja trachura* | *Raja radula* | **Dalatiidae** |
| ***Chiloscyllium*** | ***Irolita*** | *Raja rouxi* | ***Dalatias*** |
| *Chiloscyllium arabicum* | *Irolita waitii* | *Raja straeleni* | *Dalatias licha* |
| *Chiloscyllium griseum* | ***Pavoraja*** | *Raja texana* | ***Euprotomicrus*** |
| *Chiloscyllium hasseltii* | *Pavoraja alleni* | *Raja undulata* | *Euprotomicrus bispinatus* |
| *Chiloscyllium indicum* | *Pavoraja nitida* | ***Rajella*** | ***Isistius*** |
| *Chiloscyllium plagiosum* | ***Psammobatis*** | *Rajella bathyphila* | *Isistius brasiliensis* |
| *Chiloscyllium punctatum* | *Psammobatis lentiginosa* | *Rajella bigelowi* | *Isistius plutodus* |
| ***Hemiscyllium*** | ***Sympterygia*** | *Rajella caudaspinosa* | ***Squaliolus*** |
| *Hemiscyllium freycineti* | *Sympterygia acuta* | *Rajella dissimilis* | *Squaliolus laticaudus* |
| *Hemiscyllium ocellatum* | *Sympterygia bonapartii* | *Rajella fyllae* | **Echinorhinidae** |
| *Hemiscyllium trispeculare* | *Sympterygia brevicaudata* | *Rajella lintea* | ***Echinorhinus*** |
| **Orectolobidae** | **Gurgesiellidae** | ***Rostroraja*** | *Echinorhinus brucus* |
| ***Eucrossorhinus*** | ***Cruriraja*** | *Rostroraja alba* | *Echinorhinus cookei* |
| *Eucrossorhinus dasypogon* | *Cruriraja parcomaculata* | ***Spiniraja*** | **Etmopteridae** |
| ***Orectolobus*** | ***Gurgesiella*** | *Spiniraja whitleyi* | ***Aculeola*** |
| *Orectolobus japonicus* | *Gurgesiella dorsalifera* | ***Zearaja*** | *Aculeola nigra* |
| *Orectolobus maculatus* | **Rajidae** | *Zearaja chilensis* | ***Centroscyllium*** |
| *Orectolobus ornatus* | ***Amblyraja*** | **Rhinopristiformes** | *Centroscyllium fabricii* |
| *Orectolobus wardi* | *Amblyraja georgiana* | **Glaucostegidae** | *Centroscyllium granulatum* |
| ***Sutorectus*** | *Amblyraja hyperborea* | ***Glaucostegus*** | *Centroscyllium kamoharai* |
| *Sutorectus tentaculatus* | *Amblyraja jenseni* | *Glaucostegus granulatus* | *Centroscyllium nigrum* |
| **Parascylliidae** | *Amblyraja radiata* | *Glaucostegus typus* | *Centroscyllium ornatum* |
| ***Cirrhoscyllium*** | *Amblyraja taaf* | **Pristidae** | ***Etmopterus*** |
| *Cirrhoscyllium expolitum* | ***Beringraja*** | ***Anoxypristis*** | *Etmopterus benchleyi* |
| *Cirrhoscyllium japonicum* | *Beringraja inornata* | *Anoxypristis cuspidata* | *Etmopterus brachyurus* |
| ***Parascyllium*** | *Beringraja rhina* | ***Pristis*** | *Etmopterus gracilispinis* |
| *Parascyllium collare* | *Beringraja stellulata* | *Pristis microdon* | *Etmopterus granulosus* |
| *Parascyllium ferrugineum* | ***Breviraja*** | *Pristis pectinata* | *Etmopterus hillianus* |
| *Parascyllium variolatum* | *Breviraja claramaculata* | *Pristis perotteti* | *Etmopterus lucifer* |
| **Rhincodontidae** | *Breviraja marklei* | *Pristis pristis* | *Etmopterus molleri* |
| ***Rhincodon*** | *Breviraja nigriventralis* | *Pristis zijsron* | *Etmopterus polli* |
| *Rhincodon typus* | ***Dentiraja*** | **Rhinidae** | *Etmopterus princeps* |
| **Stegostomatidae** | *Dentiraja lemprieri* | ***Rhina*** | *Etmopterus pusillus* |
| ***Stegostoma*** | ***Dipturus*** | *Rhina ancylostoma* | *Etmopterus schultzi* |
| *Stegostoma fasciatum* | *Dipturus batis* | ***Rhynchobatus*** | *Etmopterus sentosus* |
| **Pristiophoriformes** | *Dipturus campbelli* | *Rhynchobatus djiddensis* | *Etmopterus spinax* |
| **Pristiophoridae** | *Dipturus doutrei* | *Rhynchobatus luebberti* | *Etmopterus virens* |
| ***Pliotrema*** | *Dipturus gudgeri* | **Rhinobatidae** | **Oxynotidae** |
| *Pliotrema warreni* | *Dipturus innominatus* | ***Acroteriobatus*** | ***Oxynotus*** |
| ***Pristiophorus*** | *Dipturus laevis* | *Acroteriobatus blochii* | *Oxynotus bruniensis* |
| *Pristiophorus cirratus* | *Dipturus leptocauda* | ***Aptychotrema*** | *Oxynotus caribbaeus* |
| *Pristiophorus japonicus* | *Dipturus nidarosiensis* | *Aptychotrema rostrata* | *Oxynotus centrina* |
| *Pristiophorus nudipinnis* | *Dipturus oxyrinchus* | *Aptychotrema vincentiana* | **Somniosidae** |
| *Pristiophorus schroederi* | *Dipturus pullopunctatus* | ***Platyrhinoidis*** | ***Centroscymnus*** |
| **Rajiformes** | ***Leucoraja*** | *Platyrhinoidis triseriata* | *Centroscymnus coelolepis* |
| **Anacanthobatidae** | *Leucoraja circularis* | ***Pseudobatos*** | *Centroscymnus crepidater* |
| ***Indobatis*** | *Leucoraja erinacea* | *Pseudobatos lentiginosus* | *Centroscymnus owstonii* |
| *Indobatis ori* | *Leucoraja fullonica* | ***Rhinobatos*** | *Centroscymnus plunketi* |
| ***Schroederobatis*** | *Leucoraja garmani* | *Rhinobatos albomaculatus* | ***Scymnodalatias*** |
| *Schroederobatis americana* | *Leucoraja leucosticta* | *Rhinobatos irvinei* | *Scymnodalatias albicauda* |
| ***Springeria*** | *Leucoraja melitensis* | *Rhinobatos rhinobatos* | ***Scymnodon*** |
| *Springeria folirostris* | *Leucoraja naevus* | ***Trygonorrhina*** | *Scymnodon ringens* |
| **Arhynchobatidae** | *Leucoraja ocellata* | *Trygonorrhina fasciata* | ***Somniosus*** |
| ***Atlantoraja*** | ***Malacoraja*** | ***Zanobatus*** | *Somniosus microcephalus* |
| *Atlantoraja castelnaui* | *Malacoraja senta* | *Zanobatus schoenleinii* | *Somniosus pacificus* |
| *Atlantoraja cyclophora* | *Malacoraja spinacidermis* | ***Zapteryx*** | *Somniosus rostratus* |
| *Atlantoraja platana* | ***Neoraja*** | *Zapteryx exasperata* | ***Zameus*** |
| ***Bathyraja*** | *Neoraja africana* | **Squaliformes** | *Zameus squamulosus* |
| *Bathyraja abyssicola* | *Neoraja caerulea* | **Centrophoridae** | **Squalidae** |
| *Bathyraja brachyurops* | *Neoraja carolinensis* | ***Centrophorus*** | ***Cirrhigaleus*** |
| *Bathyraja eatonii* | *Neoraja stehmanni* | *Centrophorus granulosus* | *Cirrhigaleus asper* |
| *Bathyraja griseocauda* | ***Raja*** | *Centrophorus harrissoni* | *Cirrhigaleus barbifer* |
| *Bathyraja interrupta* | *Raja asterias* | *Centrophorus lusitanicus* | ***Squalus*** |
| *Bathyraja irrasa* | *Raja brachyura* | *Centrophorus moluccensis* | *Squalus acanthias* |
| *Bathyraja maccaini* | *Raja clavata* | *Centrophorus squamosus* | *Squalus blainville* |
| *Bathyraja maculata* | *Raja eglanteria* | *Centrophorus uyato* | *Squalus cubensis* |

**Table S1 (continued).**

| *Squalus japonicus* | **Holocephali** | **Myxini** | **Sarcopterygii** |
| --- | --- | --- | --- |
| *Squalus megalops* | **Chimaeriformes** | **Myxiniformes** | **Coelacanthiformes** |
| *Squalus mitsukurii* | **Callorhinchidae** | **Myxinidae** | **Latimeriidae** |
| **Squatiniformes** | ***Callorhinchus*** | ***Eptatretus*** | ***Latimeria*** |
| **Squatinidae** | *Callorhinchus capensis* | *Eptatretus deani* | *Latimeria chalumnae* |
| ***Squatina*** | *Callorhinchus milii* | *Eptatretus hexatrema* |  |
| *Squatina aculeata* | **Chimaeridae** | *Eptatretus polytrema* |  |
| *Squatina africana* | ***Chimaera*** | *Eptatretus stoutii* |  |
| *Squatina argentina* | *Chimaera argiloba* | ***Myxine*** |  |
| *Squatina australis* | *Chimaera jordani* | *Myxine australis* |  |
| *Squatina californica* | *Chimaera monstrosa* | *Myxine glutinosa* |  |
| *Squatina guggenheim* | *Chimaera opalescens* | *Myxine ios* |  |
| *Squatina japonica* | ***Hydrolagus*** |  |  |
| *Squatina nebulosa* | *Hydrolagus affinis* |  |  |
| *Squatina oculata* | *Hydrolagus alberti* |  |  |
| *Squatina squatina* | *Hydrolagus colliei* |  |  |
| *Squatina tergocellata* | *Hydrolagus lemures* |  |  |
| **Torpediniformes** | *Hydrolagus melanophasma* |  |  |
| **Hypnidae** | *Hydrolagus mirabilis* |  |  |
| ***Hypnos*** | *Hydrolagus novaezealandiae* |  |  |
| *Hypnos monopterygius* | *Hydrolagus ogilbyi* |  |  |
| **Narcinidae** | **Rhinochimaeridae** |  |  |
| ***Benthobatis*** | ***Harriotta*** |  |  |
| *Benthobatis kreffti* | *Harriotta haeckeli* |  |  |
| ***Diplobatis*** | *Harriotta raleighana* |  |  |
| *Diplobatis ommata* | ***Rhinochimaera*** |  |  |
| ***Narcine*** | *Rhinochimaera atlantica* |  |  |
| *Narcine brasiliensis* | *Rhinochimaera pacifica* |  |  |
| *Narcine entemedor* |  |  |  |
| *Narcine vermiculata* |  |  |  |
| ***Narcinops*** |  |  |  |
| *Narcinops tasmaniensis* |  |  |  |
| *Narcinops westraliensis* |  |  |  |
| **Platyrhinidae** |  |  |  |
| ***Platyrhina*** |  |  |  |
| *Platyrhina sinensis* |  |  |  |
| **Torpedinidae** |  |  |  |
| ***Torpedo*** |  |  |  |
| *Torpedo mackayana* |  |  |  |
| *Torpedo marmorata* |  |  |  |
| *Torpedo torpedo* |  |  |  |

**Table S2:** Species richness, average phylogenetic diversity (AvPD), sum of the higher taxonomic levels (STL), and sum of the higher taxonomic levels divided by the number of species (STL/spp) of all fish in 5-degree latitude bands in depth zones. – indicates not analysed.

| **Latitude (°)** | **Whole water column** | | | |  | **Surface zone (0 -200 m)** | | | |  | **Middle zone (201 - 1000 m)** | | | |  | **Deep zone (1001 - 6000 m)** | | | |
| --- | --- | --- | --- | --- | --- | --- | --- | --- | --- | --- | --- | --- | --- | --- | --- | --- | --- | --- | --- |
|  | **Species richness** | **AvPD** | **STL** | **STL/spp** |  | **Species richness** | **AvPD** | **STL** | **STL/spp** |  | **Species richness** | **AvPD** | **STL** | **STL/spp** |  | **Species richness** | **AvPD** | **STL** | **STL/spp** |
| **-75** | 72 | 46.1 | 190 | 2.6 |  | 8 | – | – | – |  | 50 | 41.8 | 130 | 2.6 |  | 14 | 60.0 | 81 | 5.8 |
| **-70** | 95 | 45.8 | 252 | 2.7 |  | 13 | 46.0 | 55 | 4.2 |  | 61 | 39.3 | 159 | 2.6 |  | 21 | 60.0 | 120 | 5.7 |
| **-65** | 120 | 46.8 | 325 | 2.7 |  | 16 | 48.0 | 73 | 4.6 |  | 74 | 41.8 | 196 | 2.6 |  | 30 | 59.3 | 153 | 5.1 |
| **-60** | 177 | 47.1 | 511 | 2.9 |  | 24 | 56.5 | 122 | 5.1 |  | 97 | 46.3 | 331 | 3.4 |  | 56 | 52.7 | 238 | 4.3 |
| **-55** | 265 | 46.2 | 776 | 2.9 |  | 59 | 60.8 | 313 | 5.3 |  | 135 | 48.2 | 489 | 3.6 |  | 71 | 51.6 | 287 | 4.0 |
| **-50** | 396 | 44.5 | 1078 | 2.7 |  | 112 | 54.6 | 527 | 4.7 |  | 193 | 47.1 | 636 | 3.3 |  | 91 | 51.1 | 352 | 3.9 |
| **-45** | 581 | 41.9 | 1481 | 2.5 |  | 231 | 51.5 | 867 | 3.8 |  | 246 | 44.6 | 732 | 3.0 |  | 104 | 48.5 | 379 | 3.6 |
| **-40** | 828 | 39.3 | 1864 | 2.3 |  | 402 | 44.9 | 1189 | 3.0 |  | 303 | 42.5 | 824 | 2.7 |  | 123 | 47.9 | 441 | 3.6 |
| **-35** | 1462 | 36.0 | 2571 | 1.8 |  | 908 | 38.2 | 1781 | 2.0 |  | 403 | 40.4 | 1011 | 2.5 |  | 151 | 47.4 | 508 | 3.4 |
| **-30** | 1997 | 33.7 | 2942 | 1.5 |  | 1406 | 33.6 | 2109 | 1.5 |  | 441 | 40.3 | 1071 | 2.4 |  | 150 | 47.8 | 517 | 3.4 |
| **-25** | 2543 | 32.5 | 3206 | 1.3 |  | 1931 | 31.7 | 2351 | 1.2 |  | 460 | 39.6 | 1084 | 2.4 |  | 152 | 47.2 | 519 | 3.4 |
| **-20** | 2777 | 31.2 | 3277 | 1.2 |  | 2129 | 30.1 | 2387 | 1.1 |  | 482 | 38.6 | 1078 | 2.2 |  | 166 | 46.1 | 541 | 3.3 |
| **-15** | 2914 | 30.6 | 3300 | 1.1 |  | 2264 | 29.5 | 2431 | 1.1 |  | 487 | 38.0 | 1067 | 2.2 |  | 163 | 45.6 | 531 | 3.3 |
| **-10** | 3090 | 30.2 | 3355 | 1.1 |  | 2419 | 29.2 | 2482 | 1.0 |  | 502 | 37.3 | 1066 | 2.1 |  | 169 | 45.2 | 544 | 3.2 |
| **-5** | 3147 | 29.9 | 3319 | 1.1 |  | 2467 | 28.8 | 2449 | 1.0 |  | 507 | 37.2 | 1054 | 2.1 |  | 173 | 45.1 | 547 | 3.2 |
| **0** | 3188 | 29.7 | 3312 | 1.0 |  | 2511 | 28.6 | 2452 | 1.0 |  | 501 | 37.1 | 1047 | 2.1 |  | 176 | 44.4 | 533 | 3.0 |
| **5** | 3299 | 29.6 | 3372 | 1.0 |  | 2595 | 28.5 | 2478 | 1.0 |  | 525 | 37.1 | 1085 | 2.1 |  | 179 | 43.8 | 534 | 3.0 |
| **10** | 3461 | 29.5 | 3466 | 1.0 |  | 2714 | 28.4 | 2564 | 0.9 |  | 558 | 36.7 | 1110 | 2.0 |  | 189 | 43.3 | 554 | 2.9 |
| **15** | 3480 | 29.4 | 3499 | 1.0 |  | 2715 | 28.6 | 2591 | 1.0 |  | 571 | 36.4 | 1125 | 2.0 |  | 194 | 43.2 | 563 | 2.9 |
| **20** | 3532 | 29.6 | 3601 | 1.0 |  | 2719 | 28.8 | 2665 | 1.0 |  | 606 | 36.3 | 1193 | 2.0 |  | 207 | 42.7 | 605 | 2.9 |
| **25** | 3533 | 29.8 | 3674 | 1.0 |  | 2679 | 29.2 | 2698 | 1.0 |  | 638 | 36.2 | 1234 | 1.9 |  | 216 | 43.0 | 620 | 2.9 |
| **30** | 3397 | 30.2 | 3688 | 1.1 |  | 2517 | 29.7 | 2653 | 1.1 |  | 666 | 36.2 | 1297 | 1.9 |  | 214 | 42.8 | 615 | 2.9 |
| **35** | 2341 | 33.2 | 3240 | 1.4 |  | 1527 | 33.9 | 2249 | 1.5 |  | 607 | 37.4 | 1246 | 2.1 |  | 207 | 43.8 | 594 | 2.9 |
| **40** | 1656 | 35.2 | 2756 | 1.7 |  | 952 | 36.7 | 1795 | 1.9 |  | 525 | 38.8 | 1193 | 2.3 |  | 179 | 44.9 | 566 | 3.2 |
| **45** | 1291 | 36.8 | 2377 | 1.8 |  | 711 | 38.0 | 1519 | 2.1 |  | 425 | 40.7 | 1059 | 2.5 |  | 155 | 46.2 | 516 | 3.3 |
| **50** | 897 | 38.1 | 1803 | 2.0 |  | 423 | 39.9 | 1023 | 2.4 |  | 340 | 42.2 | 914 | 2.7 |  | 134 | 46.4 | 451 | 3.4 |
| **55** | 750 | 39.2 | 1596 | 2.1 |  | 345 | 40.8 | 881 | 2.6 |  | 288 | 42.8 | 815 | 2.8 |  | 117 | 49.3 | 417 | 3.6 |
| **60** | 633 | 40.4 | 1429 | 2.3 |  | 292 | 41.6 | 764 | 2.6 |  | 253 | 43.4 | 750 | 3.0 |  | 88 | 51.4 | 363 | 4.1 |
| **65** | 434 | 43.9 | 1158 | 2.7 |  | 176 | 44.8 | 541 | 3.1 |  | 187 | 47.8 | 652 | 3.5 |  | 71 | 57.1 | 343 | 4.8 |
| **70** | 216 | 49.7 | 713 | 3.3 |  | 98 | 47.3 | 337 | 3.4 |  | 97 | 54.4 | 444 | 4.6 |  | 21 | 76.3 | 154 | 7.3 |
| **75** | 113 | 50.9 | 413 | 3.7 |  | 48 | 50.2 | 195 | 4.1 |  | 56 | 57.0 | 279 | 5.0 |  | 9 | – | – | – |

**Table S3:** Species richness, average phylogenetic diversity (AvPD), sum of the higher taxonomic levels (STL), and sum of the higher taxonomic levels divided by the number of species (STL/spp) of bony fish in 5-degree latitude bands in depth zones. – indicates not analysed.

| **Latitude (°)** | **Whole water column** | | | |  | **Surface zone (0 -200 m)** | | | |  | **Middle zone (201 - 1000 m)** | | | |  | **Deep zone (1001 - 6000 m)** | | | |
| --- | --- | --- | --- | --- | --- | --- | --- | --- | --- | --- | --- | --- | --- | --- | --- | --- | --- | --- | --- |
|  | **Species richness** | **AvPD** | **STL** | **STL/spp** |  | **Species richness** | **AvPD** | **STL** | **STL/spp** |  | **Species richness** | **AvPD** | **STL** | **STL/spp** |  | **Species richness** | **AvPD** | **STL** | **STL/spp** |
| **-75** | 69 | 22.6 | 167 | 2.4 |  | 7 | – | – | – |  | 48 | 24.0 | 116 | 2.4 |  | 14 | 44.7 | 77 | 5.5 |
| **-70** | 91 | 22.5 | 233 | 2.6 |  | 12 | 25.2 | 41 | 3.4 |  | 58 | 21.8 | 145 | 2.5 |  | 21 | 44.9 | 120 | 5.7 |
| **-65** | 115 | 24.4 | 292 | 2.5 |  | 14 | 28.1 | 45 | 3.2 |  | 71 | 24.8 | 182 | 2.6 |  | 30 | 43.0 | 153 | 5.1 |
| **-60** | 161 | 25.2 | 415 | 2.6 |  | 21 | 33.3 | 81 | 3.9 |  | 86 | 28.7 | 260 | 3.0 |  | 54 | 34.7 | 215 | 4.0 |
| **-55** | 217 | 26.0 | 599 | 2.8 |  | 46 | 39.8 | 212 | 4.6 |  | 106 | 29.9 | 357 | 3.4 |  | 65 | 33.0 | 247 | 3.8 |
| **-50** | 328 | 24.9 | 842 | 2.6 |  | 91 | 35.1 | 390 | 4.3 |  | 155 | 28.5 | 474 | 3.1 |  | 82 | 32.2 | 284 | 3.5 |
| **-45** | 480 | 23.9 | 1185 | 2.5 |  | 192 | 32.9 | 689 | 3.6 |  | 194 | 26.0 | 546 | 2.8 |  | 94 | 29.7 | 314 | 3.3 |
| **-40** | 672 | 22.0 | 1479 | 2.2 |  | 325 | 26.9 | 914 | 2.8 |  | 238 | 24.9 | 628 | 2.6 |  | 109 | 28.6 | 352 | 3.2 |
| **-35** | 1238 | 19.1 | 2091 | 1.7 |  | 786 | 20.7 | 1442 | 1.8 |  | 317 | 23.2 | 766 | 2.4 |  | 135 | 27.6 | 404 | 3.0 |
| **-30** | 1741 | 16.5 | 2441 | 1.4 |  | 1267 | 16.5 | 1763 | 1.4 |  | 341 | 23.0 | 811 | 2.4 |  | 133 | 28.0 | 408 | 3.1 |
| **-25** | 2293 | 15.3 | 2698 | 1.2 |  | 1788 | 15.0 | 2006 | 1.1 |  | 370 | 22.2 | 830 | 2.2 |  | 135 | 27.4 | 410 | 3.0 |
| **-20** | 2523 | 14.2 | 2790 | 1.1 |  | 1981 | 13.8 | 2057 | 1.0 |  | 393 | 21.2 | 837 | 2.1 |  | 149 | 26.5 | 432 | 2.9 |
| **-15** | 2672 | 13.9 | 2841 | 1.1 |  | 2121 | 13.4 | 2123 | 1.0 |  | 405 | 20.7 | 839 | 2.1 |  | 146 | 26.1 | 422 | 2.9 |
| **-10** | 2843 | 13.6 | 2905 | 1.0 |  | 2270 | 13.2 | 2182 | 1.0 |  | 421 | 20.2 | 845 | 2.0 |  | 152 | 26.0 | 440 | 2.9 |
| **-5** | 2904 | 13.4 | 2874 | 1.0 |  | 2319 | 12.9 | 2154 | 0.9 |  | 430 | 20.0 | 840 | 2.0 |  | 155 | 26.0 | 441 | 2.8 |
| **0** | 2949 | 13.2 | 2876 | 1.0 |  | 2363 | 12.7 | 2151 | 0.9 |  | 430 | 19.9 | 851 | 2.0 |  | 156 | 25.3 | 427 | 2.7 |
| **5** | 3055 | 13.1 | 2930 | 1.0 |  | 2446 | 12.7 | 2176 | 0.9 |  | 451 | 19.8 | 882 | 2.0 |  | 158 | 24.9 | 426 | 2.7 |
| **10** | 3196 | 13.0 | 3009 | 0.9 |  | 2556 | 12.6 | 2249 | 0.9 |  | 475 | 19.7 | 903 | 1.9 |  | 165 | 24.7 | 444 | 2.7 |
| **15** | 3202 | 13.1 | 3042 | 1.0 |  | 2550 | 12.7 | 2276 | 0.9 |  | 486 | 19.6 | 922 | 1.9 |  | 166 | 24.9 | 451 | 2.7 |
| **20** | 3243 | 13.2 | 3119 | 1.0 |  | 2550 | 12.9 | 2348 | 0.9 |  | 515 | 19.3 | 960 | 1.9 |  | 178 | 24.4 | 479 | 2.7 |
| **25** | 3245 | 13.4 | 3185 | 1.0 |  | 2516 | 13.2 | 2386 | 0.9 |  | 543 | 19.0 | 981 | 1.8 |  | 186 | 24.5 | 492 | 2.6 |
| **30** | 3122 | 13.8 | 3204 | 1.0 |  | 2366 | 13.6 | 2357 | 1.0 |  | 571 | 19.0 | 1038 | 1.8 |  | 185 | 24.2 | 487 | 2.6 |
| **35** | 2103 | 16.1 | 2782 | 1.3 |  | 1403 | 16.8 | 1958 | 1.4 |  | 523 | 20.0 | 996 | 1.9 |  | 177 | 24.8 | 470 | 2.7 |
| **40** | 1453 | 17.8 | 2327 | 1.6 |  | 849 | 19.4 | 1520 | 1.8 |  | 449 | 21.2 | 958 | 2.1 |  | 155 | 26.0 | 448 | 2.9 |
| **45** | 1139 | 18.8 | 2006 | 1.8 |  | 640 | 20.6 | 1290 | 2.0 |  | 367 | 22.5 | 837 | 2.3 |  | 132 | 27.0 | 398 | 3.0 |
| **50** | 791 | 19.7 | 1501 | 1.9 |  | 389 | 21.7 | 861 | 2.2 |  | 291 | 23.8 | 713 | 2.5 |  | 111 | 27.3 | 344 | 3.1 |
| **55** | 659 | 20.5 | 1322 | 2.0 |  | 318 | 22.2 | 730 | 2.3 |  | 246 | 24.4 | 644 | 2.6 |  | 95 | 30.4 | 310 | 3.3 |
| **60** | 552 | 21.3 | 1175 | 2.1 |  | 271 | 22.9 | 642 | 2.4 |  | 212 | 25.0 | 581 | 2.7 |  | 69 | 32.5 | 263 | 3.8 |
| **65** | 377 | 24.1 | 941 | 2.5 |  | 165 | 25.8 | 453 | 2.7 |  | 155 | 28.8 | 490 | 3.2 |  | 57 | 37.1 | 249 | 4.4 |
| **70** | 186 | 27.1 | 554 | 3.0 |  | 93 | 28.0 | 285 | 3.1 |  | 79 | 34.2 | 320 | 4.1 |  | 14 | 63.0 | 105 | 7.5 |
| **75** | 99 | 29.7 | 329 | 3.3 |  | 45 | 30.9 | 163 | 3.6 |  | 48 | 37.7 | 214 | 4.5 |  | 6 | – | – | – |

**Table S4:** Species richness, average phylogenetic diversity (AvPD), sum of the higher taxonomic levels (STL), and sum of the higher taxonomic levels divided by the number of species (STL/spp) of cartilaginous fish in 5-degree latitude bands in depth zones. – indicates not analysed.

| **Latitude (°)** | **Whole water column** | | | |  | **Surface zone (0 -200 m)** | | | |  | **Middle zone (201 - 1000 m)** | | | |  | **Deep zone (1001 - 6000 m)** | | | |
| --- | --- | --- | --- | --- | --- | --- | --- | --- | --- | --- | --- | --- | --- | --- | --- | --- | --- | --- | --- |
|  | **Species richness** | **AvPD** | **STL** | **STL/spp** |  | **Species richness** | **AvPD** | **STL** | **STL/spp** |  | **Species richness** | **AvPD** | **STL** | **STL/spp** |  | **Species richness** | **AvPD** | **STL** | **STL/spp** |
| **-75** | 3 | – | – | – |  | 1 | – | – | – |  | 2 | – | – | – |  | 0 | – | – | – |
| **-70** | 4 | – | – | – |  | 1 | – | – | – |  | 3 | – | – | – |  | 0 | – | – | – |
| **-65** | 4 | – | – | – |  | 1 | – | – | – |  | 3 | – | – | – |  | 0 | – | – | – |
| **-60** | 15 | 43.0 | 82 | 5.5 |  | 2 | – | – | – |  | 11 | 45.0 | 67 | 6.1 |  | 2 | – | – | – |
| **-55** | 47 | 30.0 | 155 | 3.3 |  | 12 | 52.1 | 75 | 6.3 |  | 29 | 35.3 | 124 | 4.3 |  | 6 | – | – | – |
| **-50** | 63 | 27.7 | 193 | 3.1 |  | 19 | 41.3 | 97 | 5.1 |  | 35 | 34.2 | 140 | 4.0 |  | 7 | – | – | – |
| **-45** | 93 | 26.3 | 244 | 2.6 |  | 37 | 38.3 | 136 | 3.7 |  | 46 | 31.3 | 159 | 3.5 |  | 7 | – | – | – |
| **-40** | 145 | 22.8 | 326 | 2.2 |  | 74 | 27.9 | 217 | 2.9 |  | 57 | 27.0 | 176 | 3.1 |  | 10 | 47.3 | 70 | 7.0 |
| **-35** | 210 | 20.0 | 405 | 1.9 |  | 117 | 23.1 | 281 | 2.4 |  | 77 | 24.6 | 209 | 2.7 |  | 12 | 50.6 | 81 | 6.8 |
| **-30** | 244 | 19.9 | 442 | 1.8 |  | 136 | 23.0 | 306 | 2.3 |  | 91 | 23.3 | 218 | 2.4 |  | 13 | 47.6 | 86 | 6.6 |
| **-25** | 240 | 19.6 | 447 | 1.9 |  | 141 | 22.4 | 311 | 2.2 |  | 82 | 23.5 | 212 | 2.6 |  | 13 | 47.6 | 86 | 6.6 |
| **-20** | 245 | 19.2 | 444 | 1.8 |  | 147 | 21.5 | 312 | 2.1 |  | 81 | 23.2 | 201 | 2.5 |  | 13 | 47.6 | 86 | 6.6 |
| **-15** | 235 | 18.6 | 418 | 1.8 |  | 143 | 20.3 | 302 | 2.1 |  | 75 | 22.6 | 187 | 2.5 |  | 13 | 47.6 | 86 | 6.6 |
| **-10** | 241 | 18.0 | 405 | 1.7 |  | 149 | 19.7 | 290 | 1.9 |  | 75 | 22.3 | 185 | 2.5 |  | 14 | 45.2 | 81 | 5.8 |
| **-5** | 237 | 18.1 | 402 | 1.7 |  | 148 | 19.9 | 287 | 1.9 |  | 71 | 22.7 | 178 | 2.5 |  | 15 | 43.6 | 83 | 5.5 |
| **0** | 234 | 18.4 | 407 | 1.7 |  | 148 | 19.9 | 293 | 2.0 |  | 66 | 23.7 | 174 | 2.6 |  | 17 | 43.6 | 83 | 4.9 |
| **5** | 239 | 18.3 | 415 | 1.7 |  | 149 | 19.8 | 294 | 2.0 |  | 69 | 23.9 | 183 | 2.7 |  | 18 | 40.4 | 85 | 4.7 |
| **10** | 259 | 17.9 | 428 | 1.7 |  | 158 | 19.8 | 307 | 1.9 |  | 77 | 22.4 | 187 | 2.4 |  | 20 | 35.0 | 85 | 4.3 |
| **15** | 272 | 17.6 | 431 | 1.6 |  | 165 | 19.7 | 310 | 1.9 |  | 79 | 21.7 | 183 | 2.3 |  | 24 | 33.0 | 87 | 3.6 |
| **20** | 281 | 17.6 | 446 | 1.6 |  | 169 | 19.6 | 318 | 1.9 |  | 84 | 21.5 | 199 | 2.4 |  | 24 | 33.0 | 87 | 3.6 |
| **25** | 276 | 17.6 | 437 | 1.6 |  | 163 | 19.5 | 311 | 1.9 |  | 85 | 22.2 | 198 | 2.3 |  | 23 | 31.9 | 87 | 3.8 |
| **30** | 260 | 17.7 | 425 | 1.6 |  | 151 | 19.6 | 290 | 1.9 |  | 82 | 22.6 | 198 | 2.4 |  | 21 | 31.9 | 87 | 4.1 |
| **35** | 222 | 19.5 | 396 | 1.8 |  | 122 | 22.5 | 274 | 2.2 |  | 72 | 24.5 | 189 | 2.6 |  | 23 | 35.7 | 87 | 3.8 |
| **40** | 186 | 19.9 | 359 | 1.9 |  | 100 | 23.6 | 246 | 2.5 |  | 64 | 25.3 | 174 | 2.7 |  | 17 | 34.3 | 81 | 4.8 |
| **45** | 136 | 20.9 | 297 | 2.2 |  | 68 | 25.0 | 193 | 2.8 |  | 47 | 28.7 | 161 | 3.4 |  | 17 | 35.8 | 83 | 4.9 |
| **50** | 89 | 24.2 | 228 | 2.6 |  | 31 | 36.0 | 125 | 4.0 |  | 37 | 30.5 | 146 | 3.9 |  | 16 | 35.7 | 74 | 4.6 |
| **55** | 75 | 25.3 | 205 | 2.7 |  | 24 | 42.2 | 119 | 5.0 |  | 31 | 29.3 | 112 | 3.6 |  | 15 | 35.7 | 74 | 4.9 |
| **60** | 68 | 25.7 | 185 | 2.7 |  | 18 | 43.8 | 94 | 5.2 |  | 33 | 28.7 | 112 | 3.4 |  | 14 | 38.7 | 69 | 4.9 |
| **65** | 47 | 29.0 | 150 | 3.2 |  | 9 | – | – | – |  | 25 | 32.2 | 99 | 4.0 |  | 11 | 48.7 | 70 | 6.4 |
| **70** | 26 | 39.4 | 111 | 4.3 |  | 4 | – | – | – |  | 15 | 42.6 | 74 | 4.9 |  | 7 | – | – | – |
| **75** | 13 | 40.6 | 70 | 5.4 |  | 3 | – | – | – |  | 7 | – | – | – |  | 3 | – | – | – |

**Table S5:** Species richness, average phylogenetic diversity (AvPD), sum of the higher taxonomic levels (STL), and sum of the higher taxonomic levels divided by the number of species (STL/spp) of all, bony and cartilaginous fish in 100 m depth bands from 0 m to 3500 m. – indicates not analysed.

| **Depth band (m)** | **All fish** | | | |  | **Bony fish** | | | |  | **Cartilaginous fish** | | | | |
| --- | --- | --- | --- | --- | --- | --- | --- | --- | --- | --- | --- | --- | --- | --- | --- |
|  | **Specires richness** | **AvPD** | **STL** | **STL/spp** |  | **Specires richness** | **AvPD** | **STL** | **STL/spp** |  | **Specires richness** | **AvPD** | **STL** | **STL/spp** |  |
| 0 - 100 | 4224 | 12.75 | 3857 | 0.91 |  | 3920 | 12.43 | 3388 | 0.86 |  | 294 | 16.03 | 413 | 1.40 |  |
| 101 - 200 | 1153 | 17.76 | 1918 | 1.66 |  | 999 | 17.23 | 1583 | 1.58 |  | 143 | 19.35 | 272 | 1.90 |  |
| 201 - 300 | 904 | 18.59 | 1617 | 1.79 |  | 777 | 17.82 | 1300 | 1.67 |  | 118 | 21.02 | 254 | 2.15 |  |
| 301 - 400 | 736 | 19.10 | 1373 | 1.87 |  | 621 | 18.57 | 1109 | 1.79 |  | 106 | 20.25 | 216 | 2.04 |  |
| 401 - 500 | 636 | 19.75 | 1248 | 1.96 |  | 537 | 19.02 | 995 | 1.85 |  | 89 | 21.65 | 200 | 2.25 |  |
| 501 - 600 | 517 | 20.93 | 1106 | 2.14 |  | 432 | 20.09 | 870 | 2.01 |  | 76 | 22.54 | 181 | 2.38 |  |
| 601 - 700 | 444 | 21.40 | 981 | 2.21 |  | 372 | 20.50 | 772 | 2.08 |  | 62 | 23.66 | 158 | 2.55 |  |
| 701 - 800 | 401 | 21.56 | 896 | 2.23 |  | 341 | 20.72 | 719 | 2.11 |  | 50 | 23.47 | 126 | 2.52 |  |
| 801 - 900 | 360 | 22.11 | 834 | 2.32 |  | 309 | 20.84 | 657 | 2.13 |  | 41 | 26.83 | 124 | 3.02 |  |
| 901 - 1000 | 278 | 24.15 | 729 | 2.62 |  | 235 | 22.67 | 564 | 2.40 |  | 33 | 29.29 | 112 | 3.39 |  |
| 1001 - 1100 | 242 | 25.54 | 685 | 2.83 |  | 209 | 24.34 | 554 | 2.65 |  | 26 | 30.77 | 94 | 3.62 |  |
| 1101 - 1200 | 206 | 26.57 | 615 | 2.99 |  | 175 | 25.26 | 488 | 2.79 |  | 25 | 31.20 | 92 | 3.68 |  |
| 1201 - 1300 | 163 | 27.81 | 517 | 3.17 |  | 142 | 26.71 | 427 | 3.01 |  | 19 | 33.33 | 76 | 4.00 |  |
| 1301 - 1400 | 131 | 29.31 | 445 | 3.40 |  | 112 | 28.45 | 366 | 3.27 |  | 17 | 32.16 | 65 | 3.82 |  |
| 1401 - 1500 | 138 | 27.29 | 427 | 3.09 |  | 120 | 26.17 | 351 | 2.93 |  | 16 | 32.50 | 62 | 3.88 |  |
| 1501 - 1600 | 129 | 27.86 | 410 | 3.18 |  | 114 | 26.43 | 338 | 2.96 |  | 13 | 33.85 | 53 | 4.08 |  |
| 1601 - 1700 | 112 | 29.64 | 386 | 3.45 |  | 100 | 27.73 | 316 | 3.16 |  | 10 | 40.67 | 51 | 5.10 |  |
| 1701 - 1800 | 100 | 31.13 | 367 | 3.67 |  | 88 | 29.17 | 297 | 3.38 |  | 10 | 40.67 | 51 | 5.10 |  |
| 1801 - 1900 | 92 | 31.30 | 340 | 3.70 |  | 84 | 28.65 | 277 | 3.30 |  | 7 | – | – | – |  |
| 1901 - 2000 | 82 | 31.38 | 304 | 3.71 |  | 76 | 28.68 | 251 | 3.30 |  | 5 | – | – | – |  |
| 2001 - 2100 | 77 | 31.34 | 285 | 3.70 |  | 72 | 29.44 | 246 | 3.42 |  | 5 | – | – | – |  |
| 2101 - 2200 | 63 | 30.90 | 229 | 3.63 |  | 62 | 29.78 | 215 | 3.47 |  | 1 | – | – | – |  |
| 2201 - 2300 | 59 | 32.09 | 225 | 3.81 |  | 58 | 30.92 | 211 | 3.64 |  | 1 | – | – | – |  |
| 2301 - 2400 | 36 | 39.07 | 175 | 4.86 |  | 36 | 39.07 | 175 | 4.86 |  | 0 | – | – | – |  |
| 2401 - 2500 | 32 | 42.50 | 172 | 5.38 |  | 32 | 42.50 | 172 | 5.38 |  | 0 | – | – | – |  |
| 2501 - 2600 | 23 | 48.41 | 144 | 6.26 |  | 23 | 48.41 | 144 | 6.26 |  | 0 | – | – | – |  |
| 2601 - 2700 | 13 | 53.33 | 98 | 7.00 |  | 13 | 53.33 | 98 | 7.00 |  | 0 | – | – | – |  |
| 2701 - 2800 | 14 | 56.67 | 105 | 7.50 |  | 14 | 56.67 | 105 | 7.50 |  | 0 | – | – | – |  |
| 2801 - 2900 | 15 | 51.11 | 100 | 6.67 |  | 15 | 51.11 | 100 | 6.67 |  | 0 | – | – | – |  |
| 2901 - 3000 | 14 | 50.00 | 91 | 6.50 |  | 14 | 50.00 | 91 | 6.50 |  | 0 | – | – | – |  |
| 3001 - 3100 | 14 | 50.95 | 93 | 6.64 |  | 14 | 50.95 | 93 | 6.64 |  | 0 | – | – | – |  |
| 3101 - 3200 | 12 | 46.67 | 72 | 6.00 |  | 12 | 46.67 | 72 | 6.00 |  | 0 | – | – | – |  |
| 3201 - 3300 | 10 | 48.67 | 63 | 6.30 |  | 10 | 48.67 | 63 | 6.30 |  | 0 | – | – | – |  |
| 3301 - 3400 | 8 | – | – | – |  | 8 | – | – | – |  | 0 | – | – | – |  |
| 3401 - 3500 | 6 | – | – | – |  | 6 | – | – | – |  | 0 | – | – | – |  |


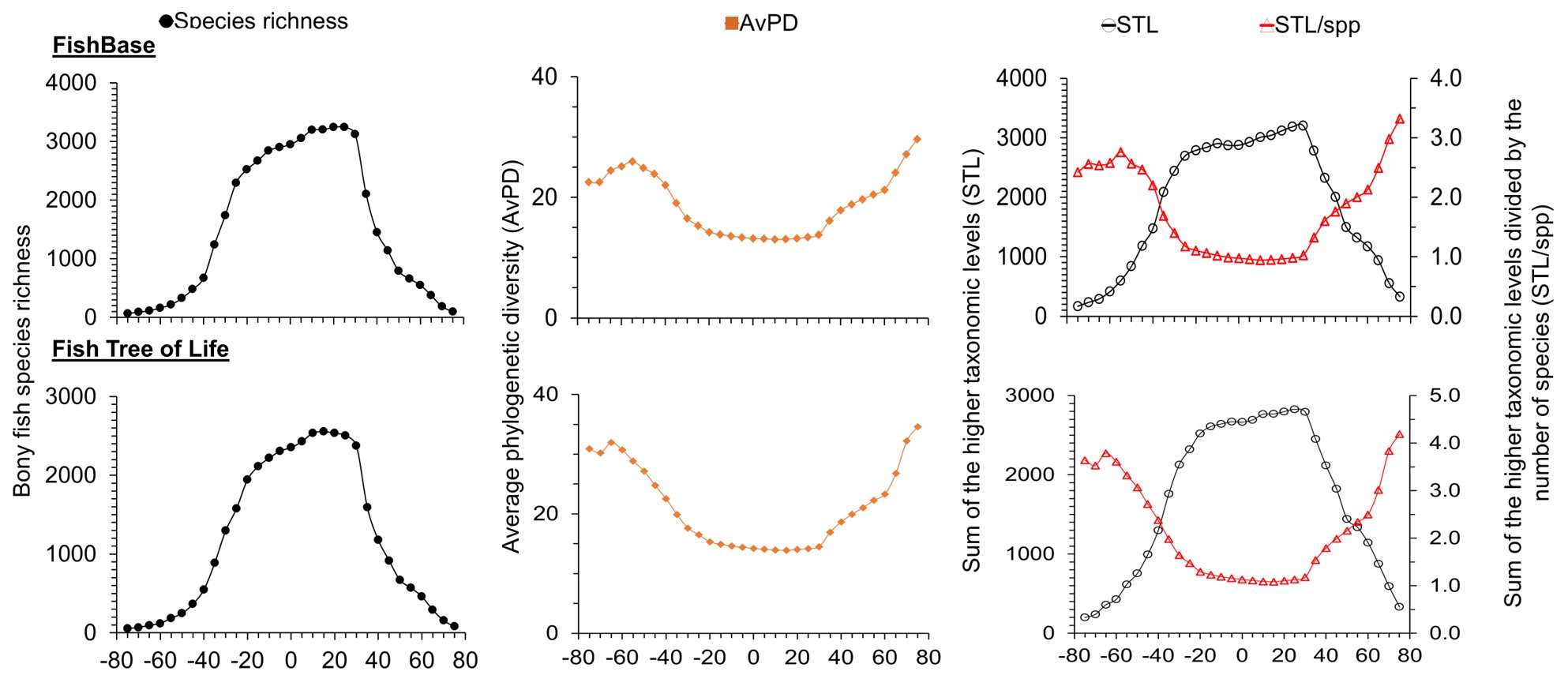


**Figure S1**. For bony fish taxonomy from FishBase and Fish Tree of Life, latitudinal gradients of species richness, average phylogenetic diversity (AvPD), the sum of the higher taxonomic levels (STL) and the sum of the higher taxonomic levels divided by the number of species (STL/spp) between 75°S and 75°N in the whole water column.


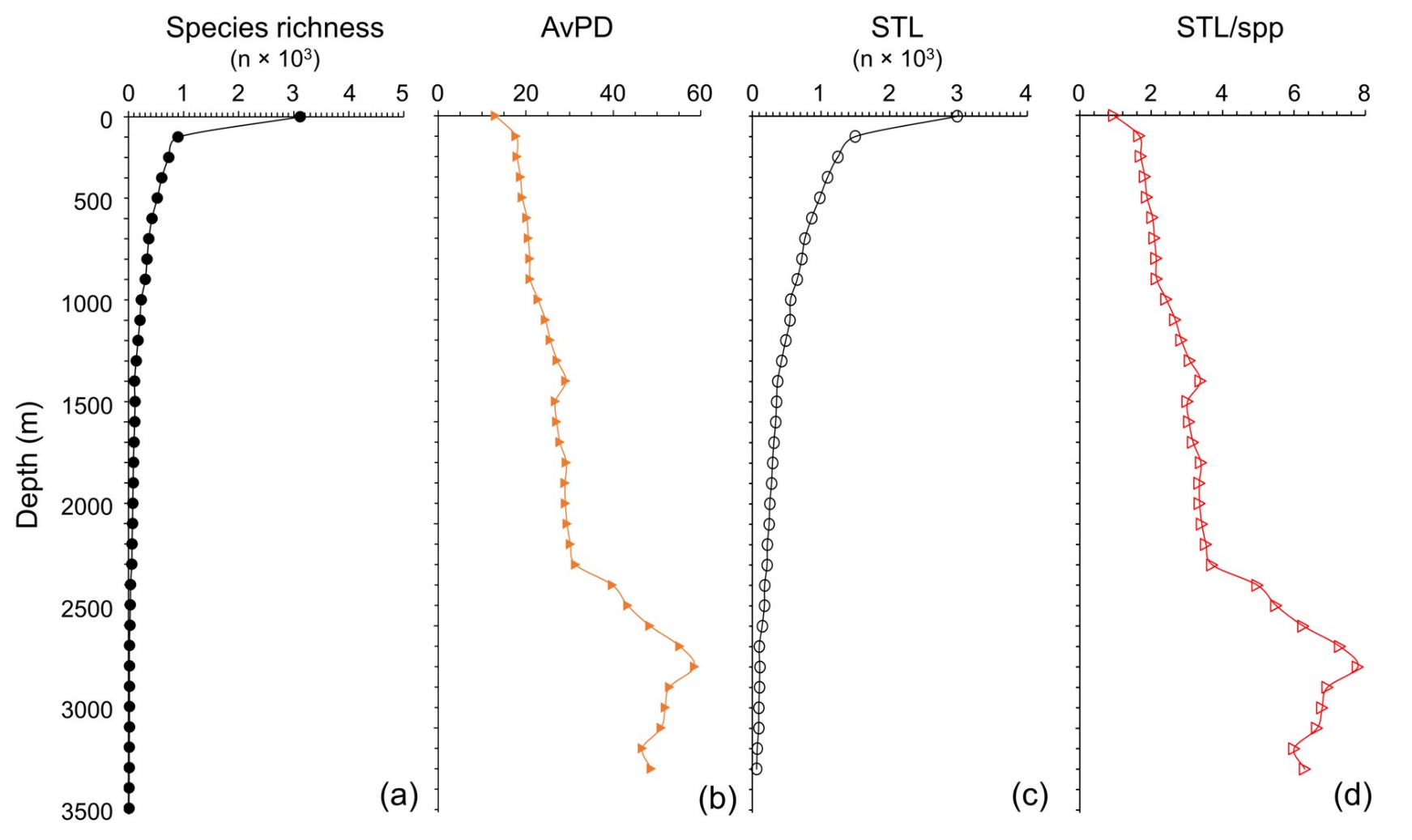


**Figure S2:** For bony fish taxonomy from Fish Tree of Life, gradients of (a) species richness, (b) average phylogenetic diversity (AvPD), (c) the sum of the higher taxonomic levels (STL), and (d) the sum of the higher taxonomic levels divided by the number of species (STL/spp) in 100 m depth bands from 0 m to 3500 m.


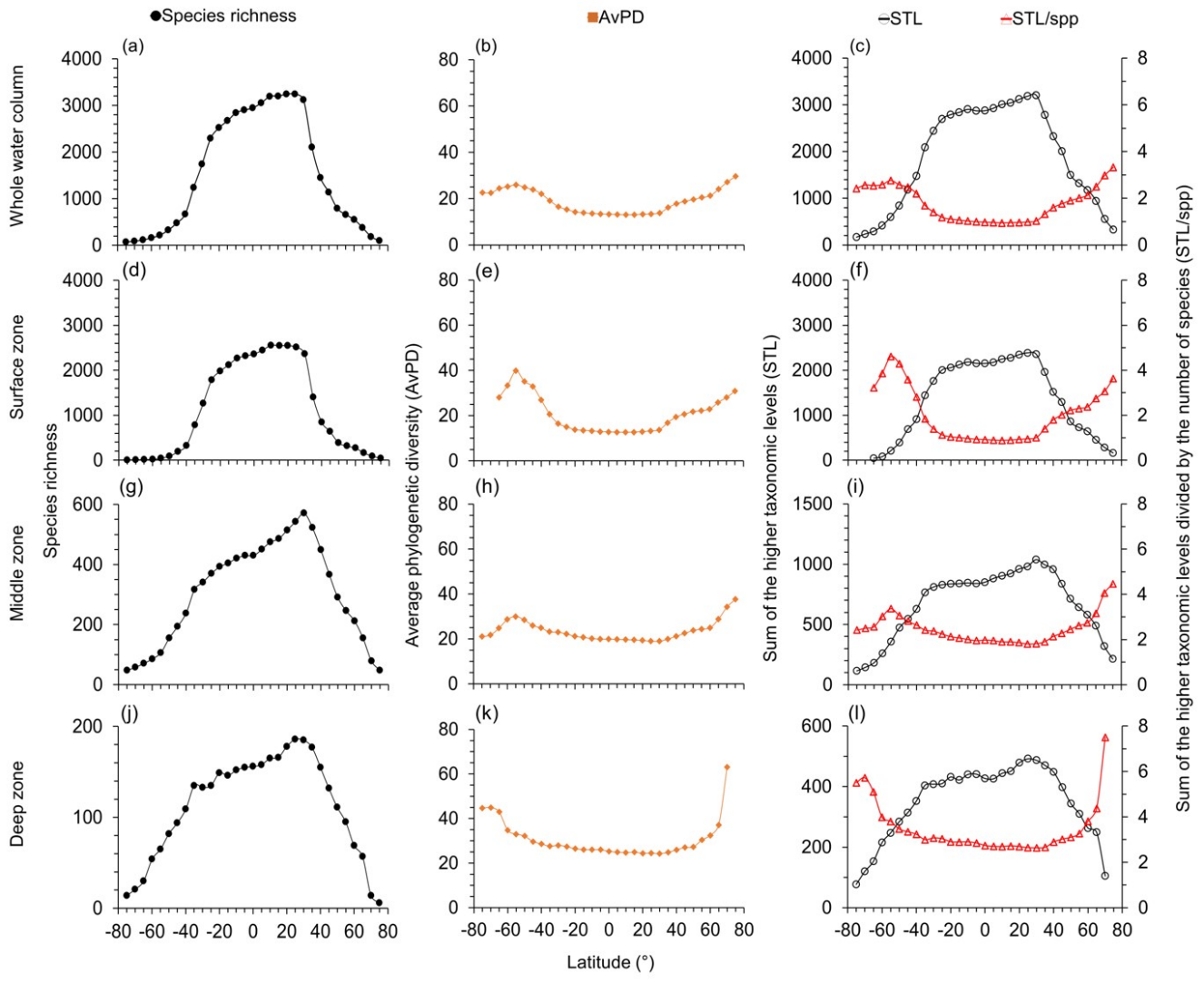


**Figure S3:** For bony fish, latitudinal gradients of species richness, average phylogenetic diversity (AvPD), the sum of the higher taxonomic levels (STL) and the sum of the higher taxonomic levels divided by the number of species (STL/spp) between 75°S and 75°N in the whole water column, surface (0 m – 200 m), middle (201 m – 1000 m), and deep (1001 m – 6000 m) zone. Note axes scales vary.


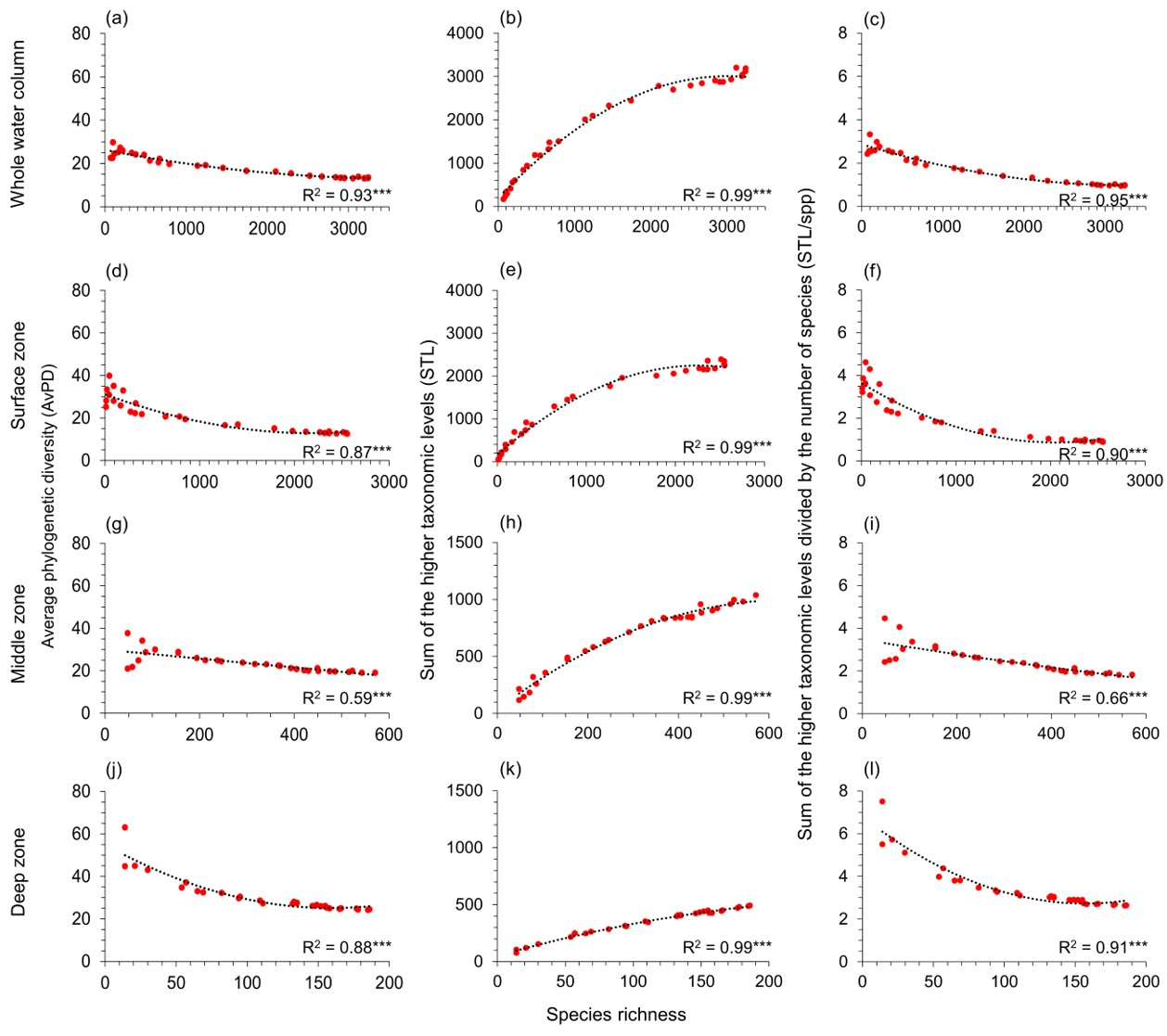


**Figure S4:** For bony fish, polynomial regression of species richness, average phylogenetic diversity (AvPD, left graphs), the sum of the higher taxonomic levels (STL, center graphs), and the sum of the higher taxonomic levels divided by the number of species (STL/spp, right graphs) in 5-degree latitude bands in the whole water column, surface (0 m – 200 m), middle (201 m – 1000 m), and deep (1001 m – 6000 m) zone. *** indicates *p*-value < 0.001. Note axes scales vary.


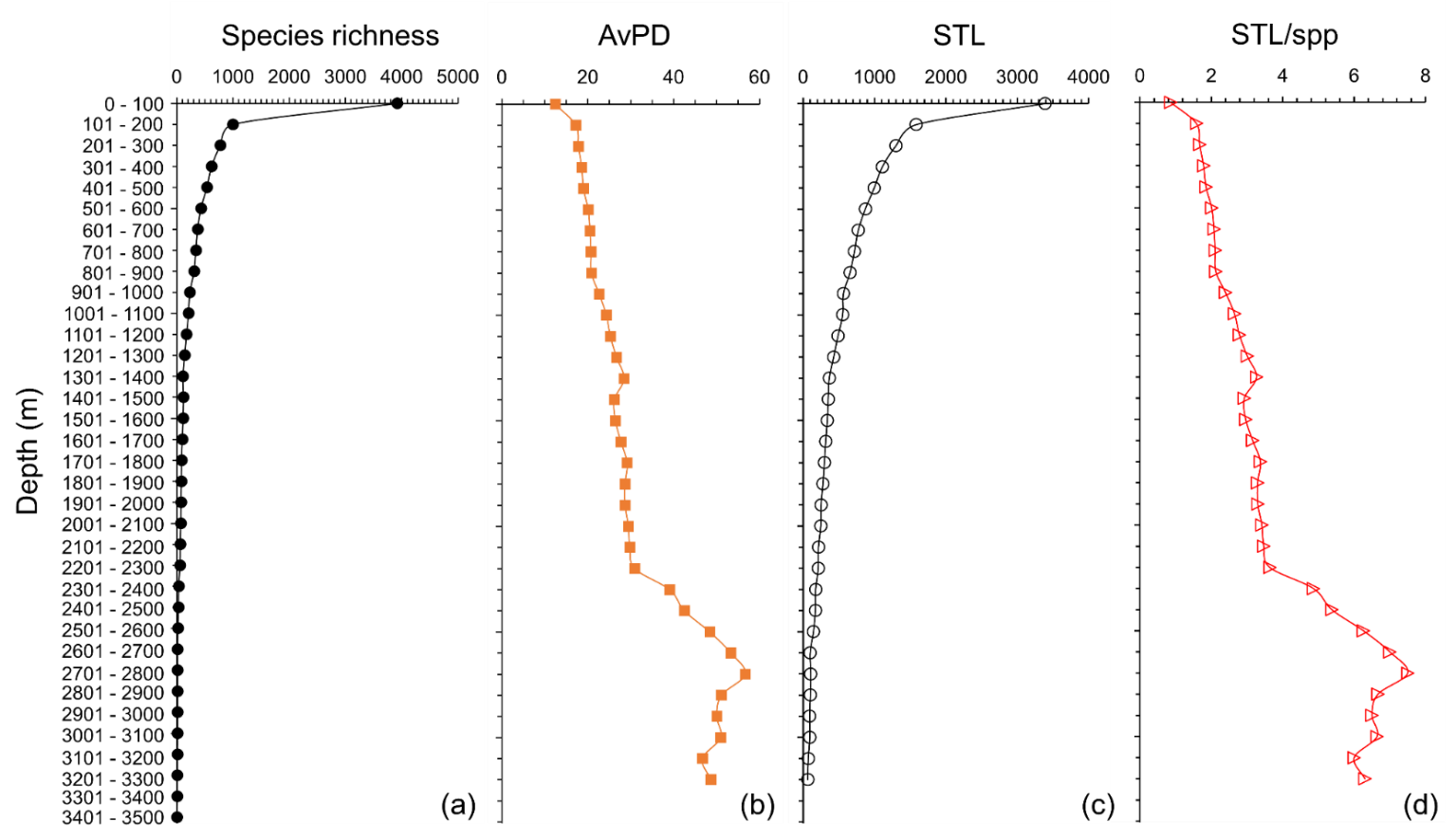


**Figure S5:** For bony fish, gradients of (a) species richness, (b) average phylogenetic diversity (AvPD), (c) the sum of the higher taxonomic levels (STL), and (d) the sum of the higher taxonomic levels divided by the number of species (STL/spp) in 100 m depth bands from 0 m to 3500 m.
